# Metal‐Free, Mild, and Sustainable Synthesis of Bio‐Based Polyesters via EDC/DMAP‐Mediated Polycondensation: Structure–Property Relationships and Thermal Stability

**DOI:** 10.1002/cssc.70907

**Published:** 2026-07-21

**Authors:** Lucía Pedraza, Virginia Arnáiz, Mercedes Santiago‐Calvo, Javier Guerra, Enol López, Asunción Barbero

**Affiliations:** ^1^ Department of Organic Chemistry Faculty of Science Campus Miguel Delibes University of Valladolid Valladolid Spain; ^2^ Division of Transport and Energy Foundation for Transport and Energy Research and Development (CIDAUT) Parque Tecnológico de Boecillo Valladolid Spain; ^3^ Department of Organic Chemistry School of Engineering (EII) University of Valladolid Valladolid Spain

**Keywords:** 2,5‐furandicarboxylic acid‐based polyesters, bio‐based polyesters, metal‐free synthesis, succinic‐acid‐based polyesters, sustainable polymers

## Abstract

In this work, we have developed a mild, metal‐free solution polycondensation protocol mediated by EDC and DMAP that enables the synthesis of bio‐based polyesters from thermally labile monomers while overcoming key limitations of high‐temperature melt polycondensation. We have prepared FDCA‐ and succinic acid‐based homopolyesters, revealing distinct structure–property relationships between rigid furanic and flexible aliphatic systems. In addition, this methodology was extended to heteropolyesters via one‐pot and sequential strategies, enabling compositional control. Structure–property relationships reveal that FDCA‐based furanic polyesters exhibit higher *T*_g_ values and predominantly amorphous character, whereas succinic‐acid‐based analogs show higher *T*_onset_ values and selective crystallinity. This library of polymers establishes EDC/DMAP‐mediated polycondensation as a versatile platform for sustainable materials design beyond the limits of conventional melt processing.

## Graphical abstract

We developed a mild, metal‐free EDC/DMAP‐mediated polycondensation platform for bio‐based polyesters from FDCA (2,5‐furandicarboxylic acid) and succinic acid, enabling access to homopolyesters from unsaturated and aliphatic diols as well as heteropolyesters prepared by one‐pot or sequential routes. This approach overcomes the limitations of conventional melt‐polymerization and broadens the toolbox for sustainable polyester design.

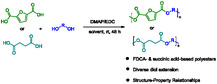

## 1 Introduction

The environmental burden of plastic waste is severe: only a small fraction of plastics are recycled at end‐of‐life, while most are incinerated or landfilled, contributing to pollution and marine contamination [1, 4]. These challenges have accelerated the transition toward circular economy principles, particularly within the packaging industry, which accounts for approximately 41% of global plastic consumption and is closely aligned with UN Sustainable Development Goal 12. While poly(ethylene terephthalate) (PET) remains the packaging industry standard due to its cost‐effectiveness and mechanical robustness, its fossil‐derived nature and resistance to biodegradation contribute to “white pollution.” Renewable bioplastics offer a strategic response, with 2,5‐furandicarboxylic acid (FDCA)—a biomass‐derived aromatic diacid—serving as a sustainable terephthalic acid surrogate. FDCA polymerization with ethylene glycol yields poly(ethylene 2,5‐furanoate) (PEF), which combines a lower carbon footprint with superior gas barrier properties compared with PET [2, 5, 9]. Beyond PEF, aliphatic furanoates like poly(butylene 2,5‐furanoate) (PBF) have been extensively studied as poly(butylene terephthalate) replacements [3, 4, 10, 11]. However, PBF exhibits limitations, including a modest glass‐transition temperature (*T*_g_), slow crystallization kinetics, and poor biodegradability. Property tuning through comonomer incorporation, such as succinic acid (SA) [12, 14] or rigid carbohydrate‐derived diols like isosorbide has been explored [15], with isosorbide, notably enhancing hardness in coatings.

However, achieving optimal thermal performance requires precise monomer design. While traditional approaches vary the glycol chain length—often at the expense of thermal stability—a more elegant strategy incorporates rigid aromatic diols or unsaturation into the diol backbone, improving thermal properties while preserving biodegradation potential as demonstrated in furan polyesters [1] and unsaturated polyurethanes [16]. Yet, the reaction of these unsaturated diols poses synthetic challenges. Conventional high‐temperature melt polytransesterification (>200 °C, Ti catalysts) is suitable for saturated glycols but fails with heat‐labile unsaturated diols, often producing discolored polymers through hydrolysis, pyrolysis and bond cleavage—particularly in systems containing unsaturated diols or 1,2‐propanediol [1, 2, 4]. Beyond thermal instability, metallic catalysts leave environmentally problematic residues, complicating applications requiring high purity [7, 9, 17, 20]. In our previous work, some unsaturated furan‐based polyesters also degraded under Ti‐catalyzed melt conditions [1]. These limitations highlight the need for alternative synthetic strategies that operate under mild conditions and avoid metal catalysts. In this context, carbodiimide‐mediated polycondensation using EDC and DMAP offers an attractive solution, enabling ester formation under mild conditions while preserving monomer functionality and avoiding metal residues [1, 11, 21, 22]. Although previous studies have addressed process optimization or thermal behavior, a systematic correlation between monomer structure, synthetic route, and final polymer performance remains underexplored.

Building on this background, EDC/DMAP‐mediated polycondensation was selected in the present work as a metal‐free alternative to conventional Ti‐catalyzed melt polycondensation, as it facilitates polyester synthesis under mild conditions while avoiding thermal degradation of labile monomers [1]. GBL was selected as the reaction solvent because it combines high polarity and low toxicity, while providing strong solvating ability and compatibility with mild polycondensation conditions. Although its relatively high boiling point may pose challenges for solvent recovery, this characteristic is also associated with low volatility, thereby reducing inhalation exposure risks during processing [23, 24]. By combining rigid and flexible diacid units with structurally diverse diols, we aim to clarify how monomer identity and synthetic route jointly influence polymer formation, composition, and thermal performance. In particular, we have compared one‐pot and sequential approaches for heteropolyester synthesis and established structure–property relationships across the resulting polyester library, thereby providing a mild, metal‐free platform for the rational design of sustainable polyesters.

## 2 Results and Discussion

### 2.1 FDCA‐Based Homopolyesters

**SCHEME 1:**
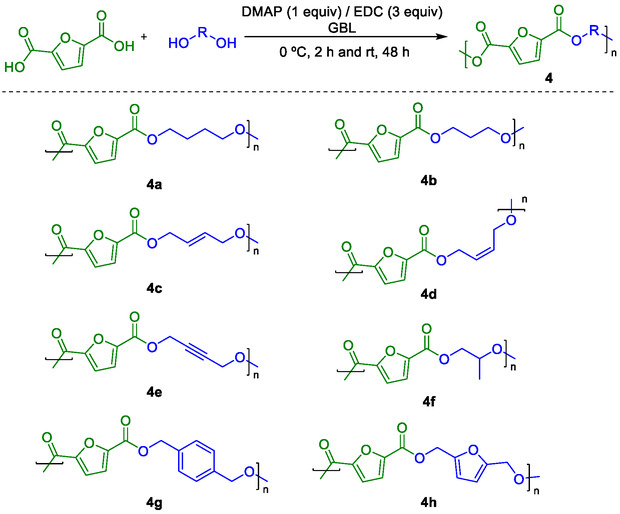
Synthesis of FDCA‐based polyesters.

The synthesis was carried out by combining FDCA (1.0 equiv) and the diol monomer (1.0 equiv) with DMAP (1.0 equiv) in a nitrogen‐purged flask, followed by the dropwise addition of EDC (3.0 equiv) dissolved in GBL (Scheme 1). This mild two‐stage protocol, consisting of initial activation at 0 °C for 2 h followed by polycondensation at room temperature for 48 h, minimizes the thermal degradation and discoloration associated with high‐temperature melt polycondensation [1]. The mechanistic pathway of this polycondensation has been described previously [11]. Unlike melt methods—which can trigger hydrolytic and pyrolytic degradation and bond cleavage in thermally labile monomers like unsaturated diols (C=C, C≡C) or 1,2‐propanediol—this methodology preserved the chemical integrity of the monomers. This greener methodology enabled the synthesis of a diverse library of metal‐free FDCA‐based homopolyesters (**4a–h**) that are difficult to access by thermal routes. Unsaturated diols such as *cis*‐ and *trans*‐2‐butene‐1,4‐diol, as well as the rigid alkyne‐containing 2‐butyne‐1,4‐diol, were fully compatible, yielding polymers **4c**–**4e**. Steric branching (**4f**), chain‐lenght variations (**4b**) and increased backbone rigidity (**4g**) pose no problems with this methodology. Notably, the incorporation of furyl units in both the diacid and the diol preserved complete structural integrity under these mild conditions (**4h**). GBL served as the reaction solvent, providing adequate solubility for the monomers and growing polymer chains during polycondensation. Polymer isolation was achieved by precipitation into water/methanol, followed by filtration and through washing to remove urea byproducts. ^1^H NMR confirmed polymer structures through characteristic furan singlet (δ 7.3–7.5 ppm) across all **4a–h**, evidencing complete monomer incorporation without degradation products.

### 2.2 Succinic Acid‐Based Homopolyesters

**SCHEME 2:**
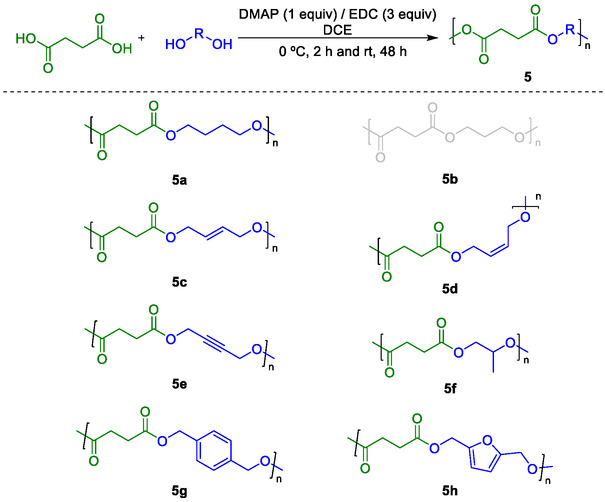
Synthesis of succinic acid‐based polyesters.

Building on the FDCA success, the same EDC/DMAP protocol—initially using GBL—was applied to synthesize aliphatic succinic acid‐based homopolyesters (**5a, c–h**, Scheme 2), creating a complementary flexible family contrasting the rigid furanics. While most systems were successfully obtained across the same structural variations (alkene/alkyne unsaturation, steric branching, homologation, diol rigidity), the branched 1,2‐propanediol‐derived system **5b** could not be isolated due to solubility issues. For the remaining materials, the flexible aliphatic character led to poor precipitation from GBL solutions and substantial filtration losses regardless of the nonsolvent (water/methanol) used. Fortunately, from 1,2‐dichloroethane (DCE) solutions, quantitative recovery of **5a, c–h** was achieved, highlighting the processing differences between rigid furanic polymers (precipitated efficiently from GBL) and more soluble succinic polymers (requiring DCE). ^1^H NMR confirmed the expected structures, with characteristic succinic CH_2_ singlets at δ 2.5–2.6 ppm across the series.

### 2.3 Heteropolyesters: One‐Pot Versus Sequential Strategies

**SCHEME 3:**
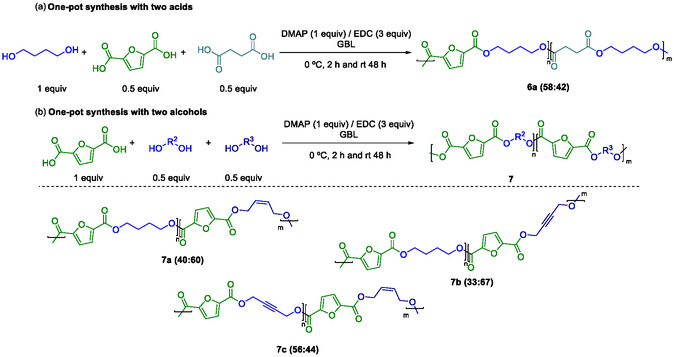
One‐pot synthesis of heteropolyesters. Copolymers **6a** and **7a–7c** are random copolymers; the sequence shown is schematic and does not represent block microstructure. Monomer ratios indicated in brackets correspond to values determined through ^1^H NMR analysis.

**SCHEME 4:**
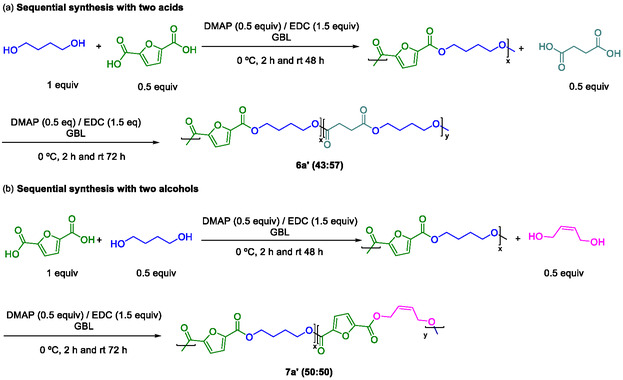
Sequential synthesis of heteropolyesters. Copolymers **6a′** and **7a′** are random copolymers; the sequence shown is schematic and does not represent block microstructure. Monomer ratios indicated in brackets correspond to values determined through ^1^H NMR analysis.

Building on the homopolyester results, we further explored the versatility of the EDC/DMAP system through copolymerization strategies to generate heteropolyesters with increased structural diversity. All heteropolyester syntheses followed the established mild EDC/DMAP solution polycondensation route through two complementary synthetic approaches: either the one‐pot (Scheme 3) or the sequential method (Scheme 4). The one‐pot method offers operational simplicity through the simultaneous addition of the monomers: two diacids with one diol (Scheme 3a) or one diacid with two different diols (Scheme 3b) in the presence of EDC and DMAP. Following the standard activation at 0 °C for 2 h to control carboxyl group reactivity, the mixture underwent polycondensation at room temperature for 48 h to promote chain growth. Heteropolyesters **6a** and **7a–7c** were recovered by precipitation in water or methanol, filtered, washed to remove urea byproducts, and dried, yielding random copolymers whose final monomer incorporation ratios were quantified through ^1^H NMR integration.

In contrast, the sequential method was designed to provide greater architectural control and was executed in two distinct stages (Scheme 4). Although it requires a longer overall total reaction time, this is an inherent consequence of its two‐step design, which involves two activation/polycondensation cycles. The first stage involved the reaction of a monomer pair (FDCA with 1,4‐butanediol, Scheme 4a) for 48 h using a slight excess of the diol. Succinic acid was then introduced in the second stage together with fresh EDC and DMAP, followed by additional activation at 0 °C for 2 and 72 h at room temperature, generating heteropolyester **6a**′ (43:57), which differed from the one‐pot **6a** (58:42). The sequential synthesis was also applied to one‐diacid/two‐diol systems, yielding **7a**′ (50:50) in contrast to the analogous one‐pot polymer **7a** (40:60), demonstrating compositional control across copolymer architectures.

### 2.4 Molecular Characterization

The chemical structures of all synthesized homo‐ and heteropolyesters were confirmed by ^1^H NMR spectroscopy (DMSO‐d_6_). MALDI‐TOF MS analysis of the oligomeric products revealed the expected repeat units characteristic of solution polycondensation, with detected species in the 1000–1700 Da range. ^1^H NMR spectroscopy (DMSO‐d_6_) confirmed complete monomer incorporation in all homo‐ and heteropolyesters. FDCA‐based homopolyesters (**4a–h**) displayed the characteristic furan ring singlet at δ 7.3–7.5 ppm, while succinic acid‐derived homopolyesters (**5a–h**) showed the diagnostic succinic CH_2_ singlet at δ 2.5–2.6 ppm. These spectral signatures confirmed the expected monomer incorporation across both families, with no evidence of degradation products or side reactions. Regarding heteropolyester derivatives, two distinct copolymer systems highlighted the synthetic control achieved by the one‐pot and sequential methodologies (Table 1). Copolymer system **6** (FDCA + SA + 1,4‐butanediol) demonstrates clear synthetic control. One‐pot synthesis of **6a** yielded a FDCA:SA ratio of 58:42, consistent with random incorporation under simultaneous monomer addition. In contrast, sequential synthesis of **6a**′ achieved a markedly different 43:57 composition accompanied by split ^1^H NMR signals, consistent with a different local chemical environment relative to the one‐pot counterpart. Copolymer system **7** (FDCA + 1,4‐butanediol + 2‐butyne‐1,4‐diol) further highlights method‐dependent microstructure. The one‐pot approach (**7a**) incorporated the diols in a 40:60 1,4‐butanediol (BDO):*cis*‐butenediol (BED) ratio and precipitated cleanly from water, consistent with random copolymer formation. Sequential synthesis (**7a′**), however, delivered a 50:50 composition but required methanol precipitation, with split NMR signals confirming distinct chemical environments arising from the stepwise polymerization process.

**TABLE 1:** Compositional analysis of heteropolyesters by ^1^H NMR.

Copolymer	Method	Composition (^1^H NMR)	NMR features	Precipitation
**6a** **6a′** **7a** **7a′**	One‐pot Sequential One‐pot Sequential	FDCA:SA 58:42 FDCA:SA 43:57 BDO:BED 40:60 BDO:BED 50:50	Standard signals Split signals Standard signals Split signals	Water/methanol Water/methanol Water Methanol

### 2.5 Thermal Characterization (Differential Scanning Calorimetry [DSC] and Thermogravimetric Analysis [TGA]) and GPC Analysis

The thermal behavior of both homopolyester families was investigated using DSC and TGA. DSC measurements followed a standardized protocol, consisting of a first heating scan to erase the thermal history, controlled cooling, and second heating scan to capture the intrinsic thermal transitions. Chemical stability during analysis was verified through reproducible heating scans with maximum temperatures maintained below TGA‐determined degradation onset temperatures. Thermal degradation onset temperatures (*T*_onset_) were determined from TGA curves as the intersection of the baseline and tangent at the point of maximum mass‐loss slope (Table 2).

**TABLE 2:** Thermal properties of FDCA‐ and succinic‐acid‐based polyesters.

Compound	*T*_g_, °C	*T*_m_, °C	*T*_onset_, °C	*T*_onset_, °C	*M*_w_	Compound	*T*_g_, °C	*T*_m_, °C	*T*_onset_, °C	*M*_w_	
**4a** * **4b** **4c** **4d** * **4e** * **4f** **4g** **4h**	87.40 47.86 68.76 51.70 74.60 71.88 93.98 —	151.2 — — — — — — —	— 338.93 277.57 — — 248.36 315.84 249.3	151.2 — — — — — — —	1824 2040 1425 n.d. n.d. n.d. 2188 1620	**5a** **5c** **5d** **5e** **5f** **5g** **5h** **6a**	— −8.01 n.d. −7.02 −16.46 62.96 13.62 −5.92	85.31 95.06 n.d. 95.19 — 102.42 163.25 126.18	337.73 191.92 — 297.99 357.54 223.59 — —	1489 5140 n.d. 3394 n.d. 3682 8588 3315**	

* Previously published; n.d., not determined.

** The *M*_w_ of **6a’** obtained by the sequential method was 5720.

The thermal behavior summarized in Table 2 reflects two distinct structure–property patterns. DSC thermograms (Figures S1–S5) show changes in the thermal transitions of different FDCA‐based series (**4a–h**). FDCA‐based polyester with 1,4‐butanediol (**4a**) is a semi‐crystalline polymer that presents both *T*_g_ and melting temperature (*T*_m_) because of its linear, flexible chains packed in an ordered manner. However, when substituted with the 1,3‐propanediol (**4b**), *T*_g_ is drastically reduced due to a more flexible and irregular chain conformation, which facilitates molecular motion at much lower temperatures (exhibits the lowest *T*_g_) and prevents the chains from aligning orderly to form crystals (*T*_m_ disappears). Incorporating unsaturated diols (**4c–4e**) and steric branching (**4f**) results in a completely amorphous polymer, causing a decrease in *T*_g_ by geometric disorder and a drastic loss of molecular symmetry, which prevents efficient chain packing. A similar amorphous behavior is observed for the phenyl‐substituted polyester (**4g**), which shows the highest *T*_g_ of the series, because its cyclic structure imparts extreme stiffness that considerably restricts molecular rotation. No thermal transitions are observed in polymers containing furyl units (**4h**) within the DSC measurement range. In addition, TGA analysis shows that the FDCA‐based polyester series have high thermal stability, characterized by a *T*_onset_ above 248 °C, reaching 338 °C for the phenyl‐containing polymer.

The succinic acid‐based polyesters display a different balance of properties. As expected for a more flexible aliphatic backbone, they generally show lower *T*_g_ values, but they also exhibit higher *T*_onset_ values in several cases, measurable melting transitions. This indicates that the succinate unit can support partial crystallization while maintaining favorable thermal stability. DSC thermograms are reported in Figures S6–S11, Supporting Information for succinic acid‐based homopolyesters (**5a–h**). Aliphatic succinic acid‐based homopolyester with 1,4‐butanediol (**5a**) produces less dense crystals (*T*_m_ around 85 °C), while the inclusion of unsaturated diols (**5c** and **5e**) provides structural rigidity and symmetry, which enhances crystallization, resulting in more compact crystals (*T*_m_ rises to around 95 °C), whereas the flexible segments contribute to a highly mobile amorphous phase that responds at very low temperatures (*T*_g_ around −7 °C). On the contrary, the incorporation of rigid rings (**5g** and **5h**) increases both *T*_g_ and *T*_m_; **5g** provides significant steric hindrance, which markedly raises *T*_g_ to around 62 °C, while **5h** containing furyl units, thanks to its compact geometry and the polarity of its oxygen atom, maximizes intermolecular dipole–dipole interactions, achieving the most efficient crystalline packing with a maximum *T*_m_ of around 163 °C and a *T*_g_ of around 13 °C. Moreover, TGA results of the series of succinic‐acid‐based polyesters reveal a high degree of structural dependence, showing a wide range of thermal stability, with a *T*_onset_ occurring at 191 °C and reaching 357 °C in the most stable polymer (**5f**). Heteropolyester **6a** is particularly noteworthy because, compared with the corresponding FDCA homopolyester, it shows a lower *T*_g_ and a clear melting transition, reflecting the flexibilizing effect of succinic acid incorporation while retaining some rigidity from the FDCA units. Taken together, these results show that FDCA‐derived polyesters yield higher glass‐transition temperatures and predominantly amorphous structures, whereas succinic acid‐derived analogs display lower *T*_g_ values together with higher *T*_onset_ values, in some cases, semicrystalline behavior (see Table S1). Thus, FDCA‐based polyesters show a higher intrinsic thermal stability than the succinic series due to the rigidity of the furan ring.This complementarity underscores the advantage of the EDC/DMAP‐mediated, metal‐free polycondensation strategy as a mild platform for tuning polyester thermal properties through simple monomer selection. Gel permeation chromatography (GPC) analysis provided additional insight into the influence of monomer structure and synthetic route on the polyester library. Representative examples were analyzed to determine their average molecular weights and dispersity. Overall, the FDCA‐based homopolyesters showed moderate *M*_w_ values in the 1.4–2.2 kDa range for the samples analyzed, indicating efficient polymer formation under the mild conditions employed. In contrast, the succinic acid‐based polyesters generally reached slightly higher and more variable *M*_w_ values, extending up to 8.6 kDa in selected cases, which suggests that the more flexible aliphatic backbone can favor chain extension. Within the heteropolyester series, the sequential methodology afforded a polyester with higher *M*_w_ (**6a′**) than the corresponding one‐pot sample **6a**, further supporting the idea that stepwise monomer addition can enhance chain growth. The dispersity values were consistent with step‐growth polycondensation behavior, and the full GPC results are provided in the Supporting Information.

## 3 Conclusion

EDC/DMAP‐mediated solution polycondensation, carried out by low‐temperature activation followed by room‐temperature polymerization, provides a versatile metal‐free platform for bio‐based polyesters, enabling the synthesis of FDCA‐furanic (4) and succinic‐acid based (5) homopolyesters that are difficult to access via high‐temperature melt processes. The one‐pot methodology affords heteropolyesters 6a and 7a with reactivity‐controlled compositions, whereas the sequential protocol enables targeted stoichiometries. Structure–property analysis reveals that FDCA‐furanics show higher *T*_g_ values that scale with diol rigidity and predominant amorphous character, whereas succinic acid‐based analogs exhibit distinct thermal profiles and, in some cases, selective crystallinity. This rigid–flexible diacid contrast suggests different opportunities: amorphous furanic polyesters may be more suitable or structural applications, whereas the more flexible succinic polyesters may be better suited to film‐forming applications. Overall, these findings show that a unified mild, metal‐free process can tune polyester thermal behavior through simple monomer selection, while avoiding harsh thermal conditions and supporting a sustainable approach to polymer design.

## Funding

This work was supported by Junta de Castilla y León (VA074G24), and Ministerio de Ciencia e Innovación (EU/PRTR TED2021‐131705B‐C21 and FPU24/01603).

## Conflicts of Interest

The authors declare no conflicts of interest.

## Supporting information

Supplementary Material

## Data Availability

The data that supports the findings of this study are available in the supplementary material of this article.
