# Supplementary material for: Metal‐Free, Mild, and Sustainable Synthesis of Bio‐Based Polyesters via EDC/DMAP‐Mediated Polycondensation: Structure–Property Relationships and Thermal Stability
Source: ChemSusChem. 2026 Jul 21;19(14):e70907. doi: 10.1002/cssc.70907 (PMC13386188; doi:10.1002/cssc.70907)
Supplement: Supplementary file 1 — Supplementary Material [file CSSC-19-e70907-s001.pdf]

Supporting information for:

Metal-free, mild, and sustainable synthesis of bio-based polyesters via EDC/DMAP-mediated polycondensation: structure–property relationships and thermal stability

Lucía Pedraza,[a] Virginia Arnáiz,[a] Mercedes Santiago-Calvo, [b] Javier Guerra,[a] Enol López,*[c] Asunción Barbero*[a]

[a] L. Pedraza, V. Arnáiz, Prof. J. Guerra, Prof. A. Barbero. Department of Organic Chemistry, Faculty of Science. University of Valladolid (UVa), Campus Miguel Delibes, 47011, Valladolid, Spain. E-mail: asuncion.barbero@uva.es

[b] Dr. M. Santiago-Calvo. Fundation for Transport and Energy Research and Development (CIDAUT). Parque Tecnológico de Boecillo, 47151, Valladolid, Spain.

[c] Dr. E. López. Department of Organic Chemistry, School of Engineering (EII). University of Valladolid (UVa), 47002 Valladolid, Spain. E-mail: enol.lopez@uva.es

Contents

General information 3
Experimental Procedures 4
References 14
NMR spectra 15
DSC: Individual thermograms 44
Thermogravimetric analysis: individual data 50
MALDI-TOF: Individual spectra 55
GPC Chromatograms 58

General information

Unless otherwise noted, experiments were carried out with dry solvents under nitrogen atmosphere. Thin layer chromatography (TLC) was performed using aluminium backed plate, pre-coated with silica gel (0.20 mm, silica gel 60) with a fluorescent indicator (254 nm) from Macherey.

Nuclear Magnetic Resonance (NMR) Spectroscopy

NMR spectra were recorded at nuclear magnetic resonance service of the Laboratory of Instrumental Techniques (L.T.I., www.laboratoriotecnicasinstrumentales.es) University of Valladolid at Varian 400 MHz (1H, 399.85 MHz; 13C, 100.61 MHz), Varian 500 MHz (1H, 500.12 MHz; 13C, 100.61 MHz) spectrometers at room temperature (25 °C). Chemical shifts (δ) are reported in parts per million (ppm) relative to the residual solvent peaks recorded, rounded to the nearest 0.01 for 1H-NMR (reference: DMSO-d6 [1H: 2.50]).

Differential Scanning Calorimetry (DCS)

DSC measurements were carried out by performing a first heating, a cooling, and a second heating to eliminate the thermal history.

Thermogravimetric Analysis (TGA)

TGA tests were performed to evaluate the thermal degradation of the synthesized polymers. Thermograms were recorded in nitrogen atmosphere from 0 to 800 °C at a heating rate of 20 °C/min using a Mettler Toledo equipment, model TGA/SDTA851e.

MALDI-TOF Mass Spectrometry

Mass spectra were recorded by MALDI-TOF laser desorption/ionization using DHB or DCTB as matrices in positive ion mode.

Gel Permeation Chromatography (GPC)

GPC was performed on a Waters system equipped with a Waters 410 Refractive Index (RI) detector. The separations were carried out at a flow rate of 1.0 mL/min using HPLC grade chloroform ($CHCl_3$) as the mobile phase. Molecular weights (M_n and M_w) and dispersities ($Đ$) were determined relative to a cubic (3rd order) calibration curve (R2 = 0.999916) established with narrow polystyrene (PS) standards ranging from 162 to 1,000,000 g/mol. Polymer solutions were prepared at a concentration of approximately 2 mg/mL in the mobile phase, and an injection volume of 20.00 µL was utilized for each analysis.

Experimental Procedures

1. Synthesis of monomers

1.1. 2,5-Furandicarboxylic Acid (FDCA)

FDCA was purchased commercially and did not require any purification processes.

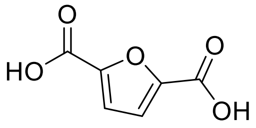

1

1.2. Synthesis of succinic acid (2)

In a 250 mL round-bottom flask, dimethyl succinate (4.18 mL, 34.0 mmol, 1.0 eq) was dissolved in a mixture of methanol (40 mL) and water (25 mL). NaOH (5.44 g, 136 mmol, 4.0 eq) was then added, and the reaction mixture was heated at 80-90 °C for 2 h under stirring. The progress of the reaction was monitored by thin-layer chromatography (TLC) using iodine as a staining agent. Upon completion, methanol was removed under reduced pressure, and the remaining aqueous solution was carefully acidified to neutral pH using concentrated HCl. The mixture was then extracted with diethyl ether to remove impurities, and the aqueous phase was concentrated under reduced pressure to afford a white precipitate. The resulting solid was washed with cold water and dried in an oven at 60 °C to afford the white product in 90 % yield. **^{1}H NMR** (500 MHz, DMSO-d_6) δ 12.14 (s, 2H), 2.41 (s, 4H). These data are in accordance with the literature. [1]

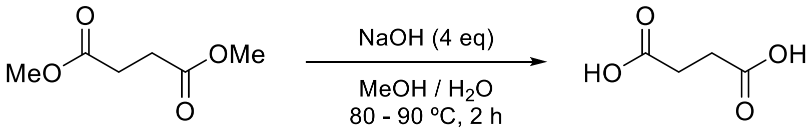

2

Scheme S1. Synthesis of **2.**

1.3. Synthesis of 1,4-phenylenedimethanol (3a)

In a 250 mL round-bottom flask, terephthalaldehyde (6.04 g, 45.0 mmol, 1.0 eq) was dissolved in methanol (MeOH, 45 mL). The solution was cooled to 0 °C using an ice bath, and $NaBH_4$ (1.19 g, 31.5 mmol, 0.7 eq) was added portionwise under continuous stirring. The reaction mixture was maintained at 0 °C for 1 h. The progress of the reaction was monitored by thin-layer chromatography (TLC). Upon completion, water (30 mL) was added, and the mixture was heated to 65 °C for 10 min. Methanol was then removed under reduced pressure, and the aqueous phase was acidified to pH 1 using concentrated hydrochloric acid, resulting in the formation of a precipitate. The product was extracted with ethyl acetate three times. The combined organic layers were dried over anhydrous $MgSO_4$ and filtered, and the solvent was removed under reduced pressure to afford **3a** as a white solid in 90 % yield and used without any further purification. **^{1}H NMR** (500 MHz, $CDCl_3$) δ 7.38 (d, *J* = 0.8 Hz, 4H), 4.72 (s, 4H), 1.65 (brs, 2H). **^{13}C NMR** (126 MHz, DMSO-d_6) δ 140.9 (C), 126.2 (CH), 62.8 (CH_2). These data are in accordance with the literature.[2]

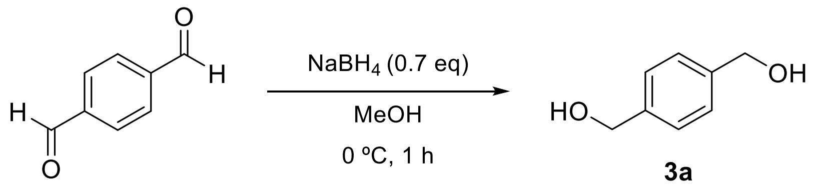

Scheme S2. Synthesis of **3a.**

1.4. Synthesis of furan-2,5-diyl dimethanol (3b)

In a 100 mL two-neck round-bottom flask, $LiAlH_4$ (0.76 g, 20.0 mmol, 5.0 eq) was introduced under nitrogen atmosphere, and anhydrous THF (25 mL) was added and cooled to 0 °C. A solution of FDCA (0.737 g, 4.0 mmol, 1.0 eq) in anhydrous THF (25 mL) was then added dropwise under stirring. The reaction mixture was maintained at 0 °C for 3 h and subsequently allowed to warm to room temperature and stirred for an additional 1 h. Upon completion, the mixture was carefully hydrolyzed at 0 °C by the slow addition of a saturated aqueous $NaHCO_3$ solution. The aqueous phase was acidified to pH 1 using 1 M HCl, and the product was extracted with ethyl acetate three times. The combined organic layers were dried over anhydrous $MgSO_4$, filtered, and the solvent was removed under reduced pressure to afford **3b** as a white solid in 51% yield. **^{1}H NMR** (500 MHz, DMSO-d_6) δ 6.18 (s, 2H, CH-furan), 5.15 (t, *J* = 5.7 Hz, 2H), 4.35 (d, *J* = 5.7 Hz, 4H). **^{13}C NMR** (126 MHz, DMSO-d_6) δ 154.7 (C), 107.4 (CH), 55.7 (CH_2). These data are in accordance with the literature.[1]

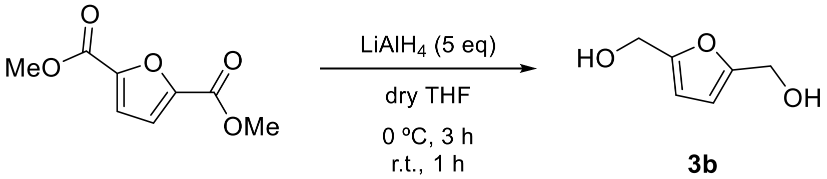

Scheme S3. Synthesis of **3b.**

1.5. Synthesis of trans-2-buten-1,4-diol (3c)

In a 100 mL round-bottom flask, $LiAlH_4$ (524 mg, 13.8 mmol, 1.2 eq) was dissolved in anhydrous THF (25 mL) under nitrogen atmosphere, and the mixture was cooled to 0 °C. A solution of 2-butyn-1,4-diol (990 mg, 11.5 mmol, 1.0 eq) in anhydrous THF (25 mL) was then added dropwise under stirring. After complete addition, the reaction mixture was heated to reflux and stirred for 2 h. The progress of the reaction was monitored by thin-layer chromatography (TLC). Upon completion, the reaction mixture was cooled to 0 °C and carefully hydrolyzed by sequential addition of water (1 mL), 15% aqueous NaOH solution (1 mL), and additional water (1 mL). The organic phase was dried over anhydrous $MgSO_4$, filtered, and the solvent was removed under reduced pressure. The crude product was purified by distillation (b.p. = 150 °C) to afford **3c** as a colorless oil in 93% yield. **^{1}H NMR** (500 MHz, DMSO-d_6) δ 5.67 (t, *J* = 2.6 Hz, 2H), 4.63 (t, *J* = 5.3 Hz, 2H), 3.92 (t, *J* = 3.7 Hz, 4H).**^{13}C NMR** (101 MHz, $CDCl_3$) δ 130.7 (CH), 63.1 (CH_2). These data are in accordance with the literature.[3]

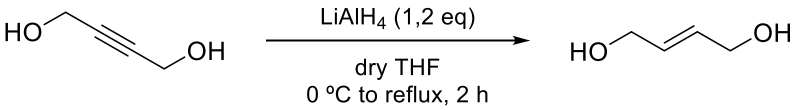

3c

Scheme S4. Synthesis of **3c.**

2. Synthesis of homopolymers 4 and 5

2.1. General Procedures

General procedure A: FDCA-based Homopolyesters

In a 100 mL three-neck round-bottom flask, FDCA (2.00 g, 12.8 mmol, 1.0 eq) and DMAP (1.56 g, 12.8 mmol, 1.0 eq) were introduced, and the system was equipped with a mechanical stirrer (130-245 rpm). The corresponding diol (12.8 mmol, 1.0 eq) was then added, followed by a solution of EDC (5.97 g, 38.4 mmol, 3.0 eq) in γ-butyrolactone (30 mL). The reaction vessel was sealed and purged with nitrogen to maintain an inert atmosphere. The resulting white suspension was stirred at 0 °C for 2 h and subsequently at room temperature for 48 h under constant stirring. Upon completion of the reaction, 100 mL of a suitable precipitation solvent (H_2O or MeOH) was added. The precipitated polymers were collected by filtration and dried in an oven at 65 °C to afford the products as white solids.

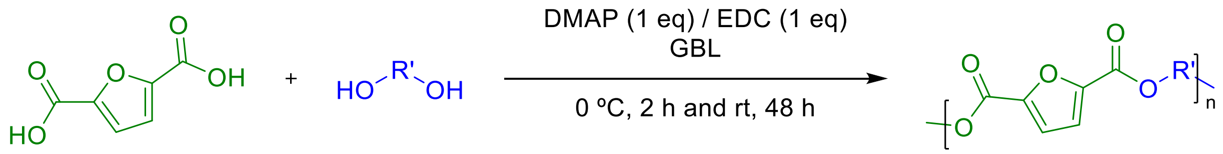

1 **3** **4a-4h**

Scheme S5. FDCA-based polymers (**4a–4h**)

General procedure B: Succinic acid-based Homopolyesters

In a 100 mL three-neck round-bottom flask, succinic acid (SA, 2.00 g, 16.9 mmol, 1.0 eq) and DMAP (2.07 g, 16.9 mmol, 1.0 eq) were introduced, and the system was equipped with a mechanical stirrer (130-245 rpm). The corresponding diol (16.9 mmol, 1.0 eq) was then added, followed by a solution of EDC (7.89 g, 50.8 mmol, 3.0 eq) in γ-butyrolactone (30 mL). The reaction vessel was sealed and purged with nitrogen to maintain an inert atmosphere. The resulting white suspension was stirred at 0 °C for 2 h and subsequently at room temperature for 48 h under constant stirring. Upon completion of the reaction, 100 mL of a suitable precipitation solvent (H_2O or MeOH) was added. The precipitated polymers were collected by filtration and dried in an oven at 65 °C to afford the products as white solids.

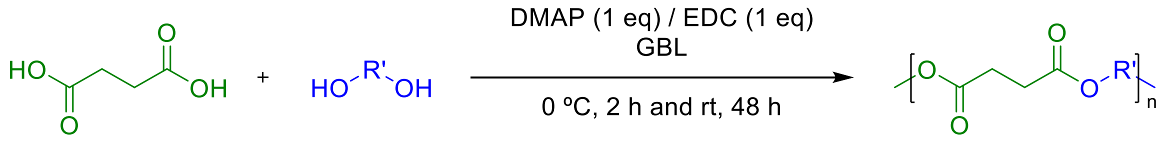

2 **3** **5a-5h**

Scheme S6. SA-based polymers (**5a–5h**).

2.2. Characterization of FDCA-Based Polymers (4a-4h)

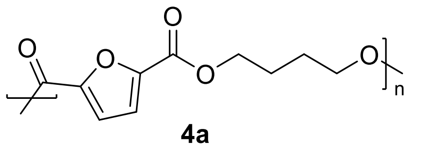

4a

Prepared according to General procedure A from FDCA and 1,4-butanediol to afford a white solid. **^{1}H NMR** (500 MHz, DMSO-*d_6*) δ 7.42-7.38 (m, 2H), 4.34 (bs, 4H), 1.81 (bs, 4H). These data are in accordance with the literature.[4] **GPC** ($CHCl_3$, PS standards): M_n = 943 g/mol, M_w = 1824 g/mol, $Đ$ = 1.93.

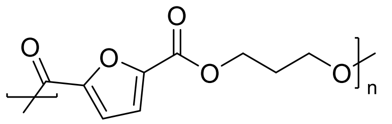

4b

Prepared according to General procedure A from FDCA and 1,3-propanediol to afford a white solid. **^{1}H NMR** (500 MHz, DMSO-*d_6*) δ 7.41-7.32 (m, 2H), 4.41 (bs, 4H), 2.15 (bs, 2H). These data are in accordance with the literature.[4] **GPC** ($CHCl_3$, PS standards): M_n = 984 g/mol, M_w = 2040 g/mol, $Đ$ = 2.07.

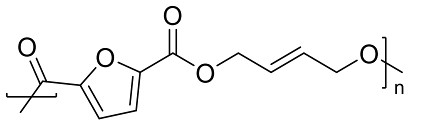

4c

Prepared according to General procedure A from FDCA and *trans*-2-buten-1,4-diol to afford a white solid. **^{1}H NMR** (500 MHz, DMSO-*d_6*) δ 7.49-7.45 (m, 2H), 6.05 (bs, 2H), 4.86 (bs, 4H). **GPC** ($CHCl_3$, PS standards): M_n = 647 g/mol, M_w = 1425 g/mol, $Đ$ = 2.20.

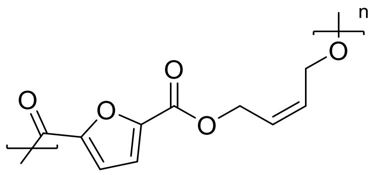

4d

Prepared according to General procedure A from FDCA and *cis*-2-buten-1,4-diol to afford a white solid. **^{1}H NMR** (500 MHz, DMSO-*d_6*) δ 7.44-7.41 (m, 2H), 5.92-5.88 (m, 2H), 5.00-4.97 (m, 4H). These data are in accordance with the literature.[5]

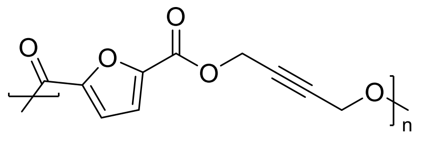

4e

Prepared according to General procedure A from FDCA and 2-butyn-1,4-diol to afford a white solid. **^{1}H NMR** (500 MHz, DMSO-d_6) δ 7.52-7.49 (m, 2H), 5.09 (bs, 4H). These data are in accordance with the literature.[5]

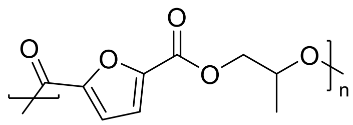

4f

Prepared according to General procedure A from FDCA and 1,2-propanediol to afford a white solid, after precipitation with H_2O. **^{1}H NMR** (500 MHz, DMSO-d_6) δ 7.38-7.29 (m, 2H), 5.36 (bs, 1H), 4.58-4.49 (m, 1H), 4.44-4.35 (m, 1H), 1.38-1.30 (m, 3H).

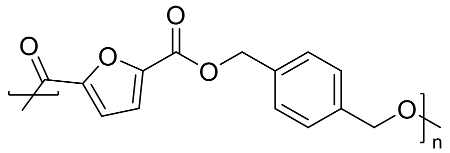

4g

Prepared according to General procedure A from FDCA and 1,4-phenylenedimethanol (**1a**) to afford a white solid. **^{1}H NMR** (500 MHz, DMSO-d_6) δ 7.49-7.45 (m, 6H), 5.38-5.31 (m, 4H). These data are in accordance with the literature.[4] **GPC** ($CHCl_3$, PS standards): M_n = 961 g/mol, M_w = 2188 g/mol, $Đ$ = 2.28.

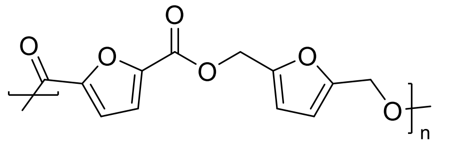

4h

Prepared according to General procedure A from FDCA and furan-2,5-diyl dimethanol (**1b**) to afford a white solid, after precipitation with H_2O. **^{1}H NMR** (500 MHz, DMSO-d_6) δ 7.43-7.40 (m, 2H), 6.65-6.61 (m, 2H), 5.32 (bs, 4H). These data are in accordance with the literature.[4] **GPC** ($CHCl_3$, PS standards): M_n = 742 g/mol, M_w = 1620 g/mol, $Đ$ = 2.18.

2.3. Characterization of SA-Based Polymers (5a-5h)

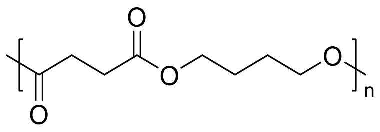

5a

Prepared according to General procedure B from SA and 1,4-butanediol to afford a white solid, after precipitation with H_2O. **^{1}H NMR** (500 MHz, DMSO-d_6) δ 4.04-3.99 (m, 4H), 2.55-2.53 (m, 4H), 1.61-1.58 (m, 4H). **GPC** ($CHCl_3$, PS standards): M_n = 1027 g/mol, M_w = 1489 g/mol, $Đ$ = 1.45.

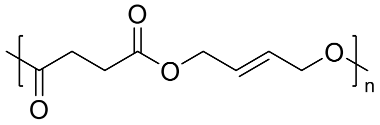

5c

Prepared according to General procedure B from SA and *trans*-2-buten-1,4-diol to afford a white solid. **^{1}H NMR** (500 MHz, DMSO-d_6) δ 5.83-5.82 (m, 2H), 4.56-4.54 (m, 4H), 2.59 (bs, 4H). **GPC** ($CHCl_3$, PS standards): M_n = 1799 g/mol, M_w = 5140 g/mol, $Đ$ = 2.86.

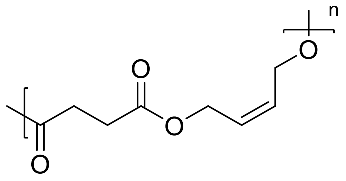

5d

Prepared according to General procedure B from SA and *cis*-2-buten-1,4-diol to afford a white solid, after precipitation with H_2O. **^{1}H NMR** (500 MHz, DMSO-d_6) δ 5.70-5.65 (m, 2H), 4.65 (d, *J*= 5.1 Hz, 4H), 2.57 (brs, 4H).

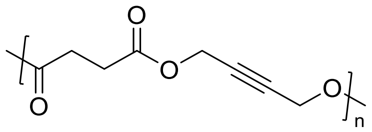

5e

Prepared according to General procedure B from SA and 2-butyn-1,4-diol to afford a white solid, after precipitation with H_2O. **^{1}H NMR** (500 MHz, DMSO-d_6) δ 4.77 (bs, 4H), 2.62-2.61 (m, 4H). **GPC** ($CHCl_3$, PS standards): M_n = 1740 g/mol, M_w = 3394 g/mol, $Đ$ = 1.95.

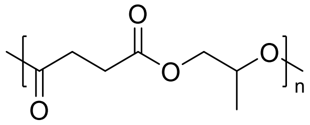

5f

Prepared according to General procedure B from SA and 1,2-propanediol to afford a white solid, after precipitation with H_2O. **^{1}H NMR** (500 MHz, DMSO-d_6) δ 5.01 (qt, *J* = 6.5, 4.1 Hz, 1H), 4.10–4.07 (m, 2H), 2.56 (s, 4H), 1.16 (d, *J* = 6.5 Hz, 3H).

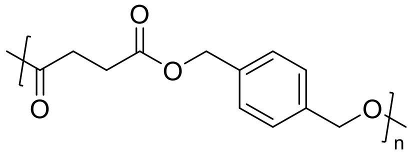

5g

Prepared according to General procedure B from SA and 1,4-phenylenedimethanol (**1a**) to afford a white solid, after precipitation with H_2O. **^{1}H NMR** (500 MHz, DMSO-d_6) δ 7.32 (bs, 4H), 5.09-5.05 (m, 4H), 2.63 (bs, 4H). **GPC** ($CHCl_3$, PS standards): M_n = 1753 g/mol, M_w = 3682 g/mol, *Đ* = 2.10.

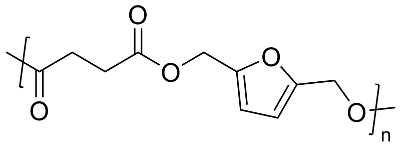

5h

Prepared according to General procedure B from SA and furan-2,5-diyl dimethanol (**1b**) to afford a white solid, after precipitation with H_2O. **^{1}H NMR** (500 MHz, DMSO-d_6) δ 6.50-6.49 (m, 2H), 5.03-5.01 (m, 4H), 2.60-2.58 (m, 4H). **GPC** ($CHCl_3$, PS standards): M_n = 3356 g/mol, M_w = 8588 g/mol, *Đ* = 2.56.

2.4. Synthesis of heteropolyesters 6 and 7

General Procedure C: One-pot method

In a 100 mL three-neck round-bottom flask, the three monomers were introduced in the desired stoichiometric ratio (diacid:diol1:diol2, 1.0:0.5:0.5 eq) or alternatively, (diol:diacid1:diacid2, 1.0:0.5:0.5 eq), together with DMAP (1.0 eq). The system was equipped with a mechanical stirrer (130–245 rpm), and a solution of EDC (3.0 eq) in γ-butyrolactone (30 mL) was added under nitrogen atmosphere. The resulting suspension was stirred at 0 °C for 2 h and subsequently at room temperature for 48 h. The heteropolymers were precipitated by addition of 100 mL of H_2O or MeOH, filtered, washed with water, and dried at 65 °C to afford white solids.

General Procedure D: Sequential method

In a 100 mL three-neck round-bottom flask, 1.0 eq of the diacid or diol and a second monomer (0.5 eq) were introduced together with DMAP (0.5 eq). A solution of EDC (1.5 eq) in γ-butyrolactone (30 mL) was added under nitrogen. The reaction mixture was stirred at 0 °C for 2 h and then at room temperature for 48 h. The third monomer (0.5 eq) was added along with additional EDC (1.5 eq) and DMAP (0.5 eq). The mixture was stirred at 0 °C for 2 h and then at room temperature for 72 h. The heteropolymers were precipitated by addition of 100 mL of H_2O or MeOH, filtered, washed with water, and dried at 65 °C to afford white solids.

2.5. Characterization of Heteropolyesters 6-7

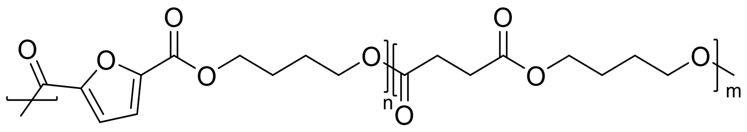

6a (FDCA:SA = 58:42, by NMR)

Prepared according to the General Procedure C from FDCA and SA using 1,4-butanediol to afford a white solid after precipitation with H_2O or MeOH. **^{1}H NMR** (500 MHz, DMSO-d_6) δ 7.39 (bs, 2H), 4.33 (bs, 2H), 4.31-4.25 (m, 2H), 4.08-4.03 (m, 2H), 4.03-3.97 (m, 2H), 2.54 (bs, 4H), 1.82 (bs, 2H), 1.75-1.70 (m, 2H), 1.70-1.64 (m, 2H), 1.61-1.56 (m, 2H). **GPC** ($CHCl_3$, PS standards): M_n = 1555 g/mol, M_w = 3315 g/mol, $Đ$ = 2.13.

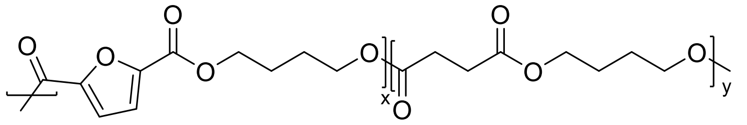

6a' (FDCA:SA = 43:57, by NMR)

Prepared according to General Procedure D from FDCA and SA (43:57 in NMR) with 1,4-butanediol to afford a white solid, after precipitation with H_2O or MeOH. **^{1}H NMR** (500 MHz, DMSO-d_6) δ 7.39 (bs, 2H), 4.36-4.32 (m, 2H), 4.32-4.27 (m, 2H), 4.08-4.03 (m, 2H), 4.03-3.98 (m, 2H), 2.54 (bs, 4H), 1.81 (bs, 2H), 1.73 (bs, 2H), 1.67 (bs, 2H), 1.59 (bs, 2H). **GPC** ($CHCl_3$, PS standards): M_n = 2629 g/mol, M_w = 5720 g/mol, $Đ$ = 2.18.

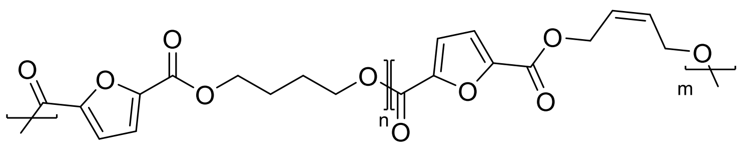

7a (FDCA-based copolymer; diol ratio = 40:60, by NMR)

Prepared according to the General Procedure C from FDCA using 1,4-butanediol and *cis*-2-buten-1,4-diol to afford a white solid after precipitation with H_2O. **^{1}H NMR** (500 MHz, DMSO-d_6) δ 7.46-7.36 (m, 4H), 5.90 (bs, 2H), 4.98 (bs, 4H), 4.37-4.27 (m, 4H), 1.81 (bs, 4H).

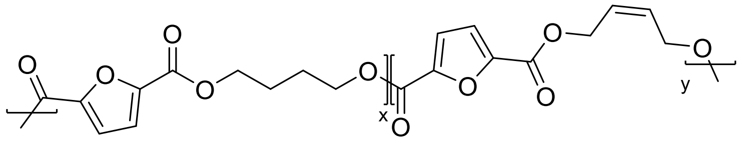

7a' (FDCA-based copolymer; diol ratio = 50:50, by NMR)

Prepared according to the General Procedure D from FDCA using 1,4-butanediol and *cis*-2-buten-1,4-diol to afford a white solid after precipitation with MeOH. **^{1}H NMR** (500 MHz, DMSO-d_6) δ 7.45-7.35 (m, 4H), 5.90 (bs, 2H), 4.98 (bs, 4H), 4.34 (bs, 4H), 1.81 (bs, 4H).

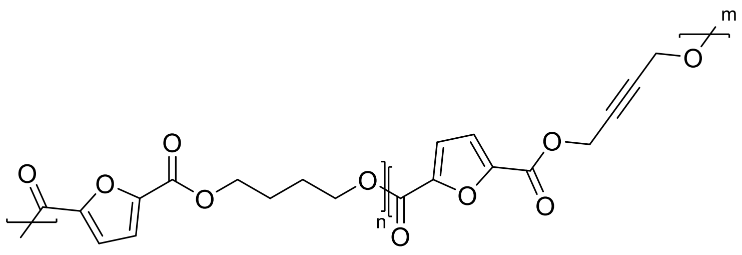

7b (FDCA-based copolymer; diol ratio = 33:67, by NMR)

Prepared according to the General Procedure C from FDCA using 1,4-butanediol and 2-butyn-1,4-diol to afford a white solid after precipitation with MeOH. [1]**H NMR** (500 MHz, DMSO-d_6) δ 7.52-7.37 (m, 4H), 5.10-5.05 (m, 4H), 4.34 (bs, 2H), 1.81 (bs, 2H).

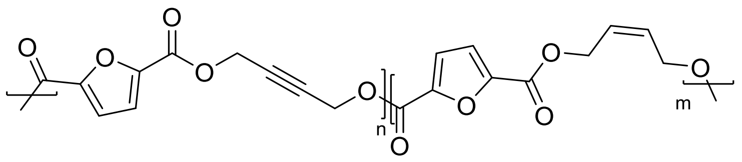

7c (FDCA-based copolymer; diol ratio = 56:44, by NMR)

Prepared according to the General Procedure C from FDCA using 2-butyn-1,4-diol and cis-2-buten-1,4-diol to afford a white solid after precipitation with MeOH. [1]**H NMR** (500 MHz, DMSO-d_6) δ 7.52-7.40 (m, 4H), 5.93-5.86 (m, 2H), 5.09 (bs, 4H), 4.99 (bs, 4H).

Table S1. Structure-properties relationship for FDCA and succinic acid-based Homopolyesters.

**Structural modification**	**Structure–property relationship**
**FDCA *vs* Succinic acid-based Homopolyester backbone**	The rigid aromatic furan ring in FDCA increases backbone stiffness, resulting in higher T_g values. On the contrary, the more flexible succinic acid backbone leads to lower T_g values, higher M_w under the same polymerization conditions, and have a Tm.
**Unsaturated diols**	The C≡C bond is more linear and rigid than the C=C bond, leading to higher T_g values for alkyne-containing polyesters.
***cis vs trans* alkenes**	The geometry of the double bond influences chain conformation and packing, which may account for differences in T_g between the *cis*- and *trans*-derived polymers (*e.g.* **4c** and **4d**).
**Branched aliphatic diols**	Alkyl branching introduces steric hindrance that disrupts chain packing and reduces crystallinity. This modification generally promotes a more amorphous character (e.g. **4f** and **5f**).
**Rigid aromatic/cyclic diols**	Incorporation of rigid diols markedly increases backbone stiffness, producing the highest T_g values within each polyester family while still offering satisfactory M_w values.
**Furan-containing diols**	Introduction of a furan ring preserves structural integrity and promotes intermolecular interactions, contributing to high T_m values (particularly for **5h**) while remaining compatible with efficient polymerization and relatively high M_w.

**Thermal stability (T_{onset})**	Thermal stability remains generally high across the polyester library, with no clear dependence on a single structural feature.

References

[1] M. S. Ortiz, J. G. Alvarado, F. Zambrano, R. Marquez, "Surfactants produced from carbohydrate derivatives: A review of the biobased building blocks used in their synthesis" *J Surfact & Detergents* **2022**, *25*, 147–183.

[2] S. W. Chaikin, W. G. Brown, "Reduction of Aldehydes, Ketones and Acid Chlorides by Sodium Borohydride" *J. Am. Chem. Soc.* **1949**, *71*, 122–125.

[3] Y. B. Kiran, H. Wakamatsu, Y. Natori, H. Takahata, Y. Yoshimura, "Design and synthesis of a nucleoside and a phosphonate analogue constructed on a branched-threo-tetrofuranose skeleton" *Tetrahedron Letters* **2013**, *54*, 3949–3952.

[4] M. Gomes, A. Gandini, A. J. D. Silvestre, B. Reis, "Synthesis and characterization of poly(2,5-furan dicarboxylate)s based on a variety of diols" *J. Polym. Sci. A Polym. Chem.* **2011**, *49*, 3759–3768.

[5] V. Arnáiz, L. Pedraza, C. Díez-Poza, M. Santiago-Calvo, E. López, A. Barbero, "Exploration of Structure–Property Relationships by Altering Glycol Backbone Unsaturation (Double/Triple Bonds) in Furan-Based Polyesters" *Eur J Org Chem* **2025**, *28*, e202500576.

NMR spectra

Comparative ^{1}H NMR Spectra between the crude reaction mixture and the washed polyester 4a

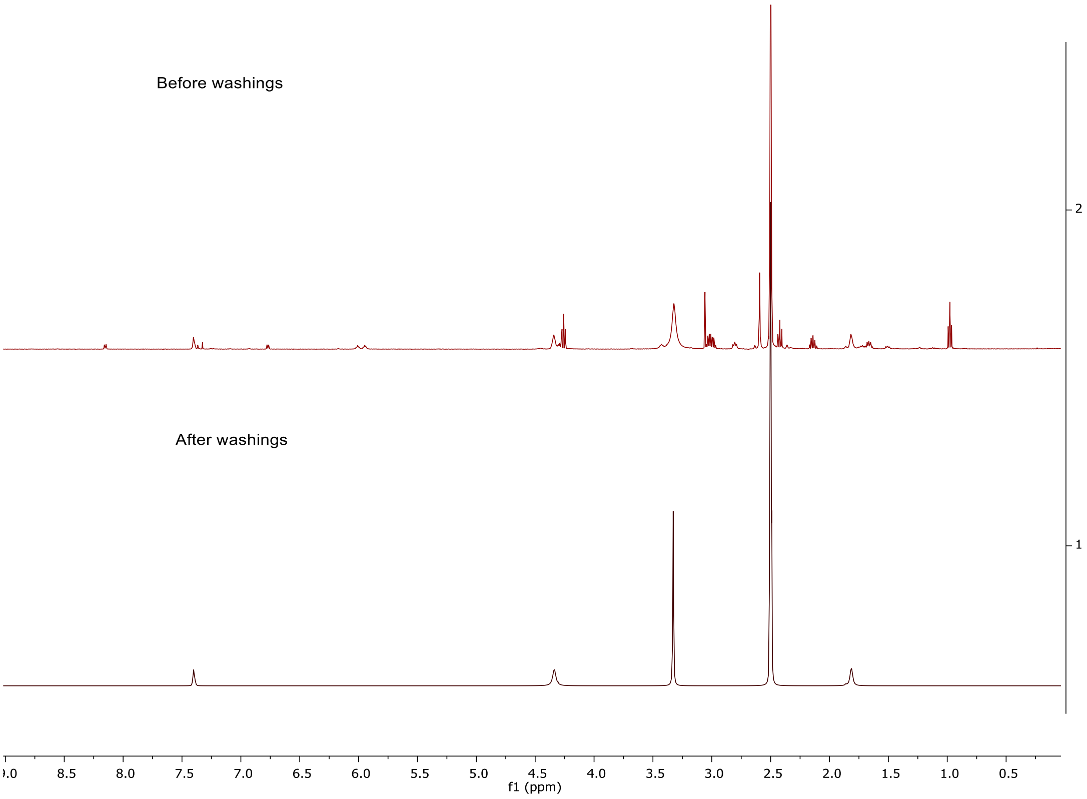

Comparative ^{1}H NMR Spectra between the crude reaction mixture and the washed polyester 5g

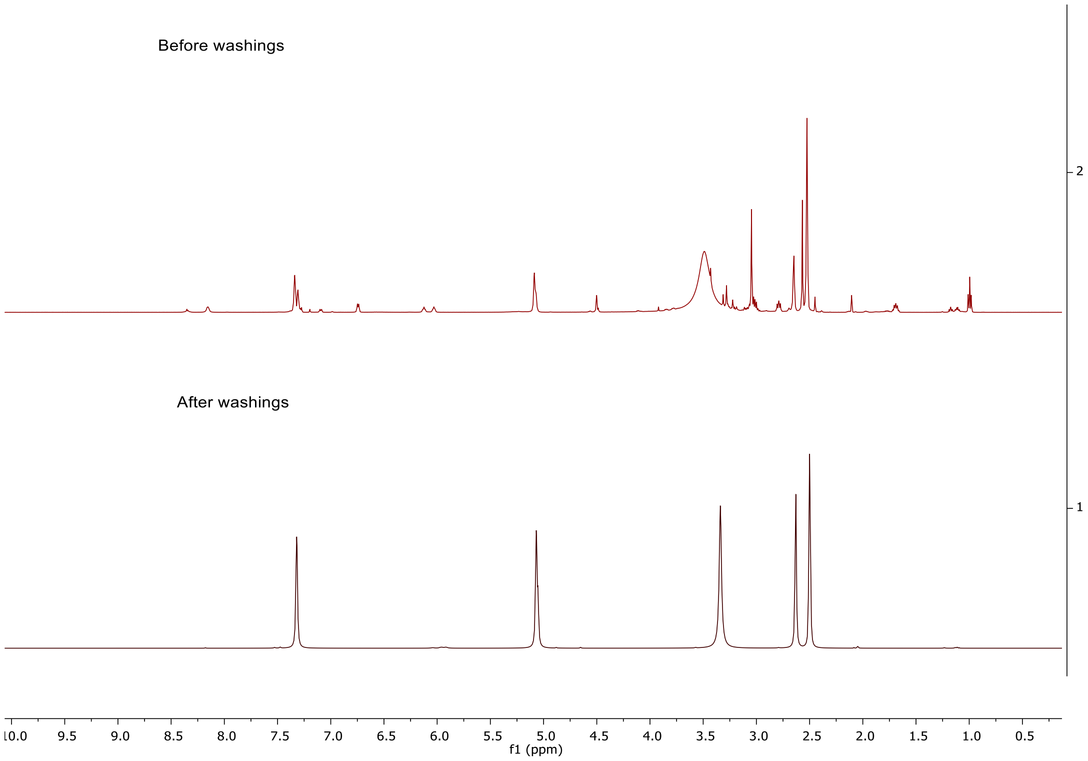

NMR of pure compounds 1-7

^{1}**H NMR** (500 MHz, DMSO-d_6)

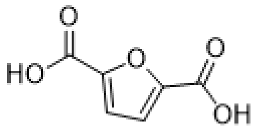

1

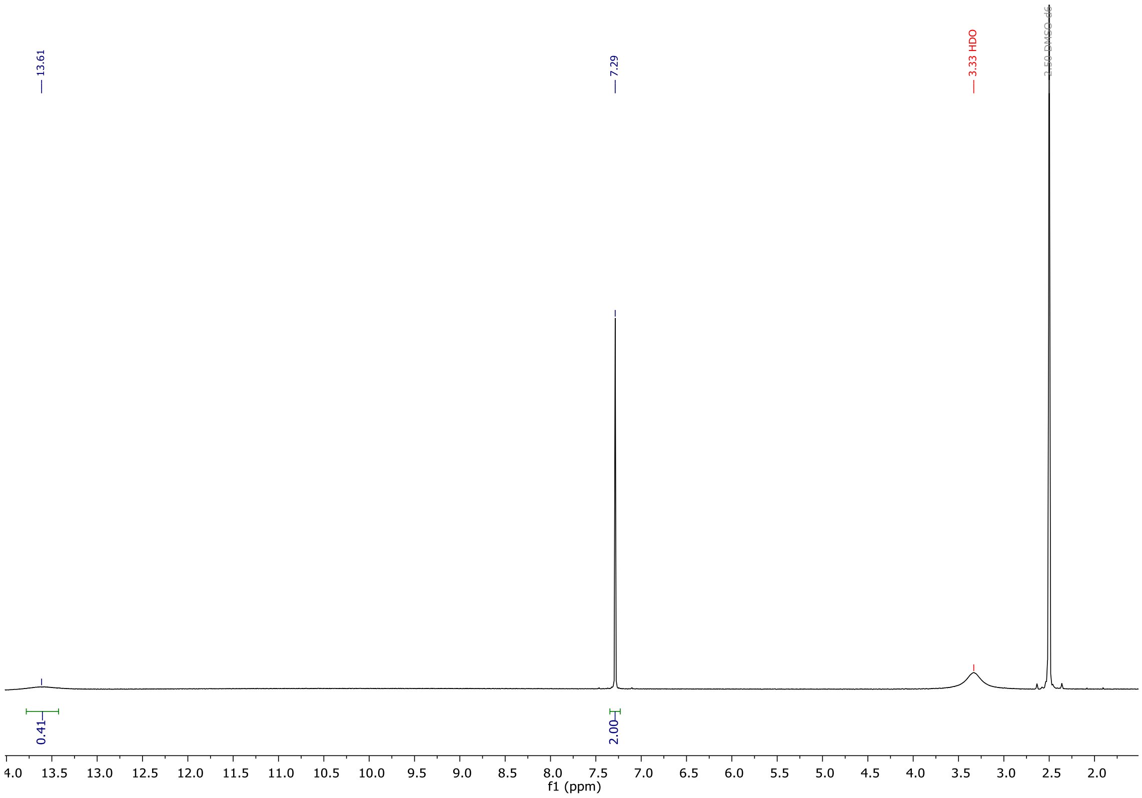
13.61
7.29
3.33 HDO
2.50 DMSO-d6
0.41
2.00
f1 (ppm)

^{1}H NMR (500 MHz, DMSO-d_6)

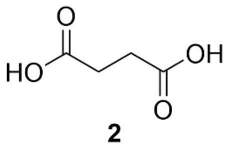

2

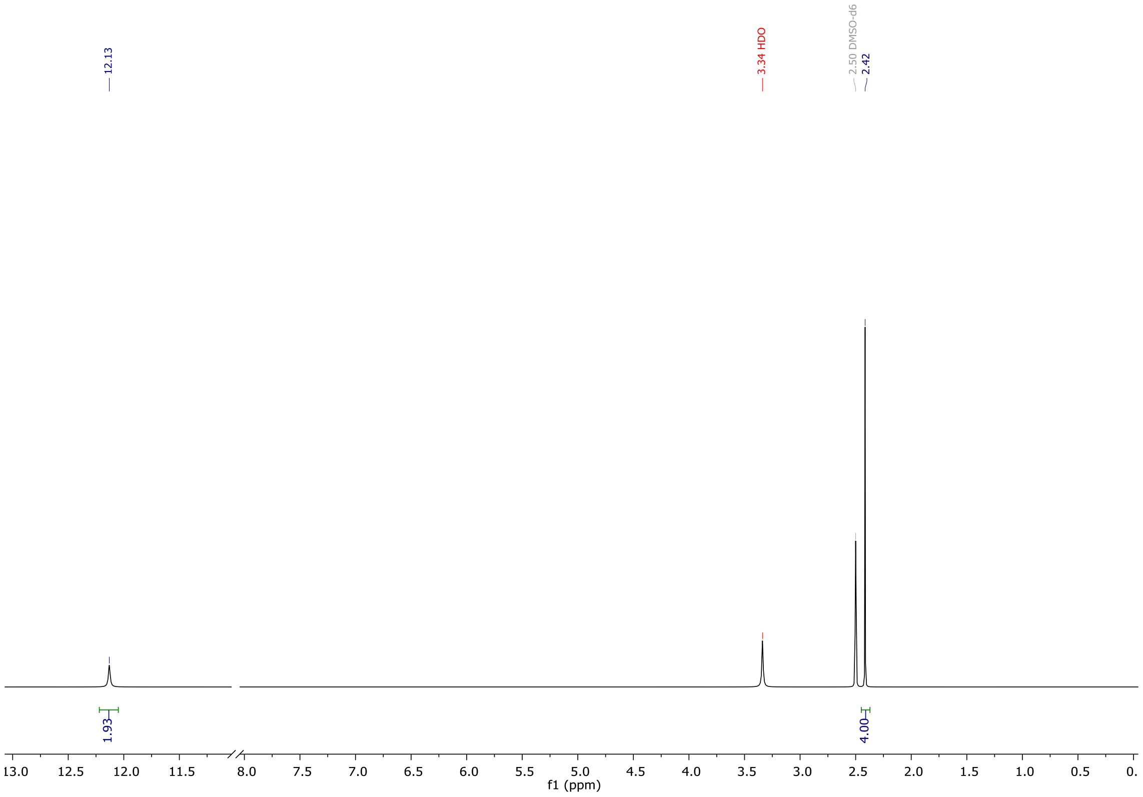

^{1}H NMR (500 MHz, $CDCl_3$)

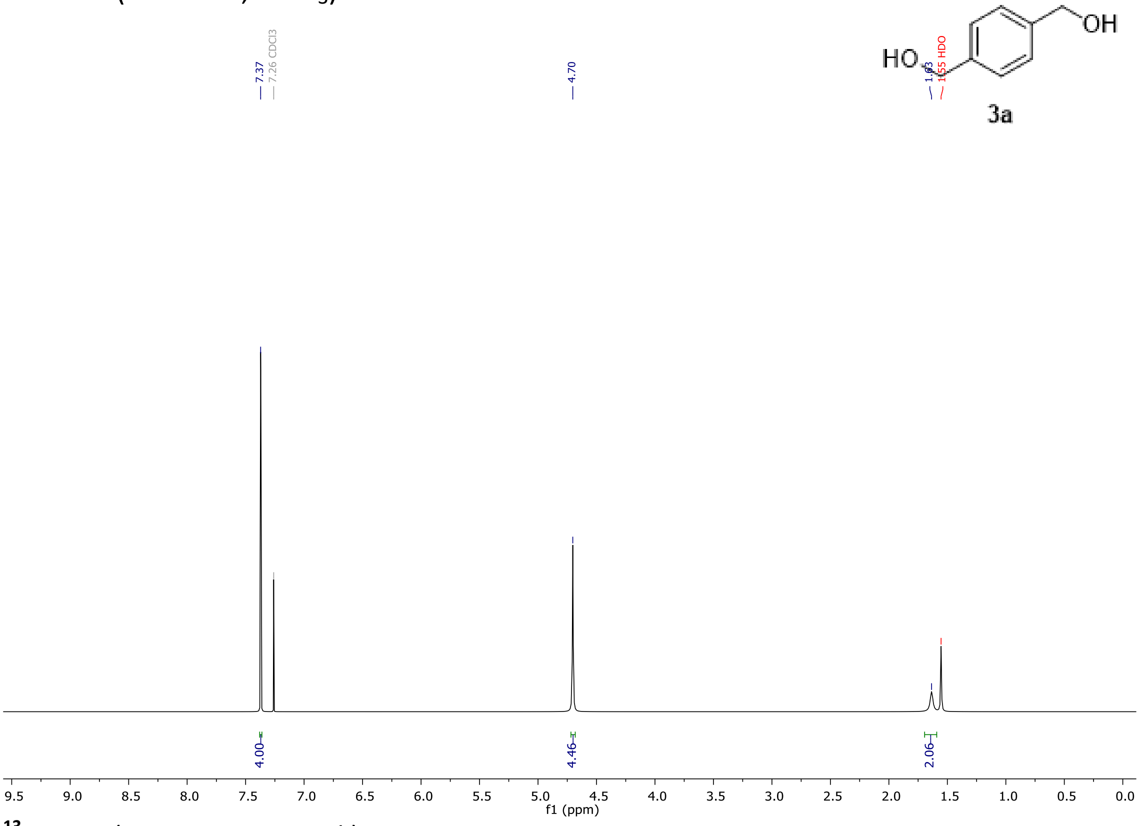

3a

^{13}C NMR (126 MHz, DMSO-d_6)

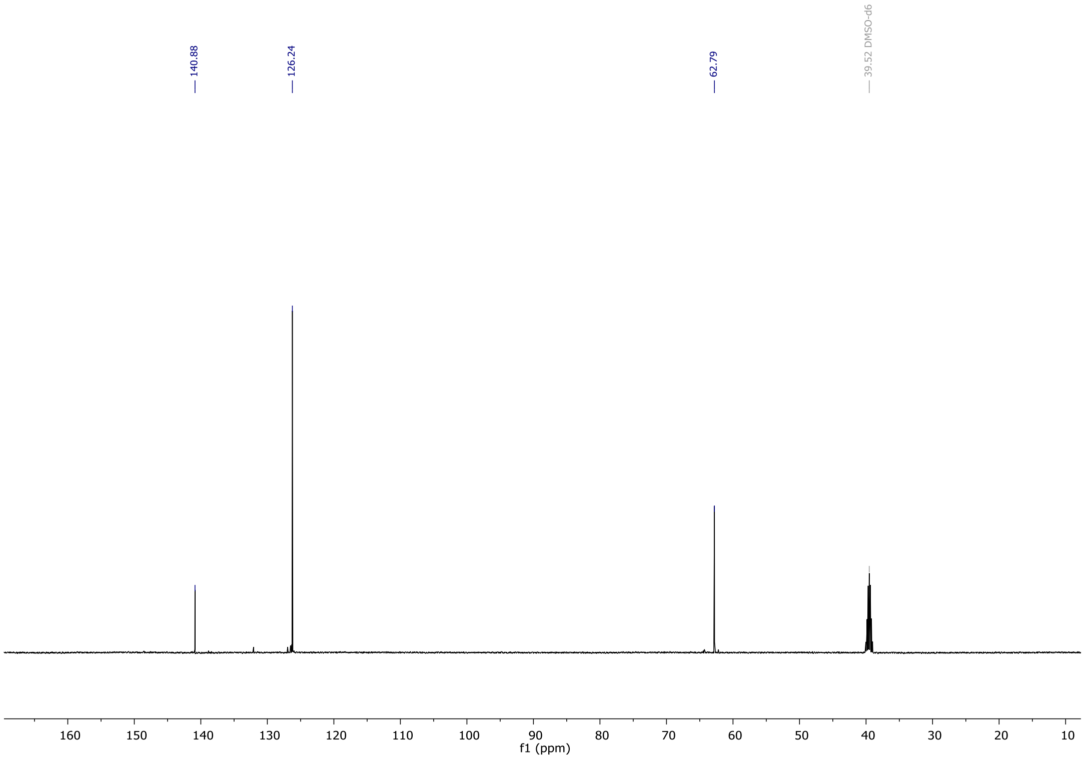

^{1}H NMR (500 MHz, DMSO-d_6)

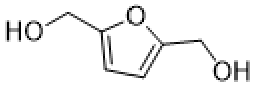

3b

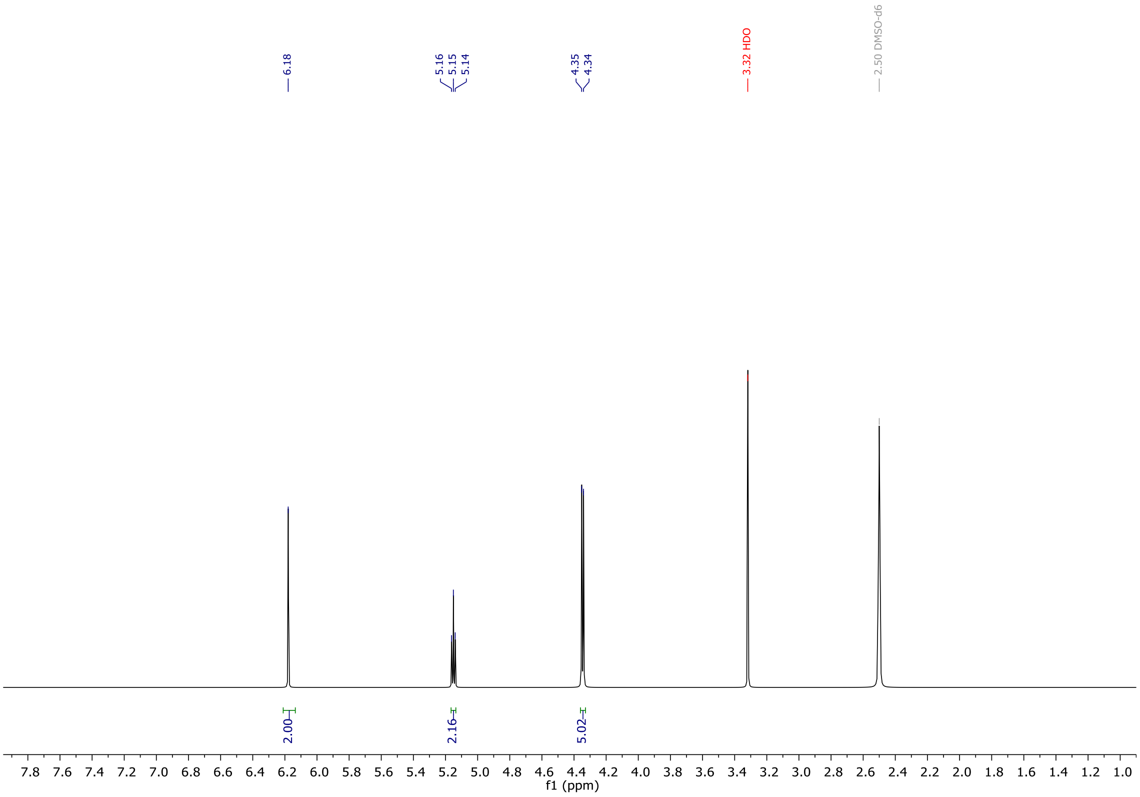

^{13}C NMR (126 MHz, DMSO-d_6)

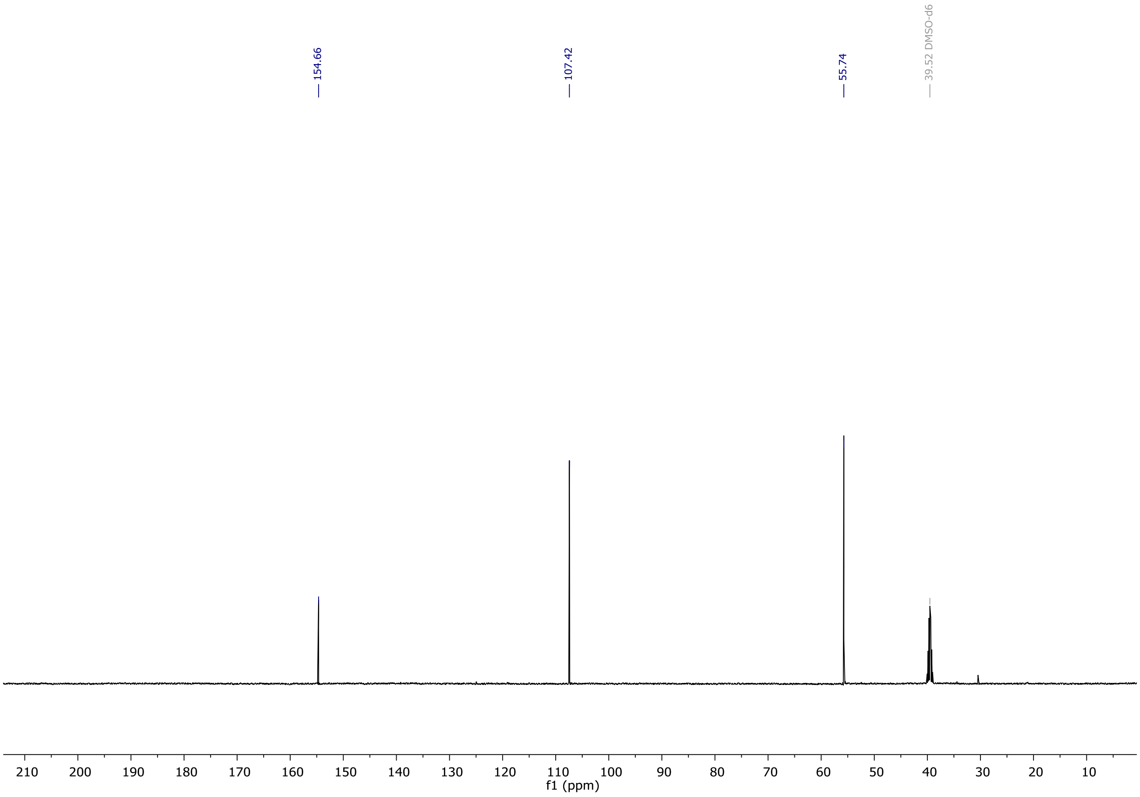

^{1}H NMR (500 MHz, DMSO-d_6)

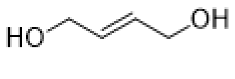

3c

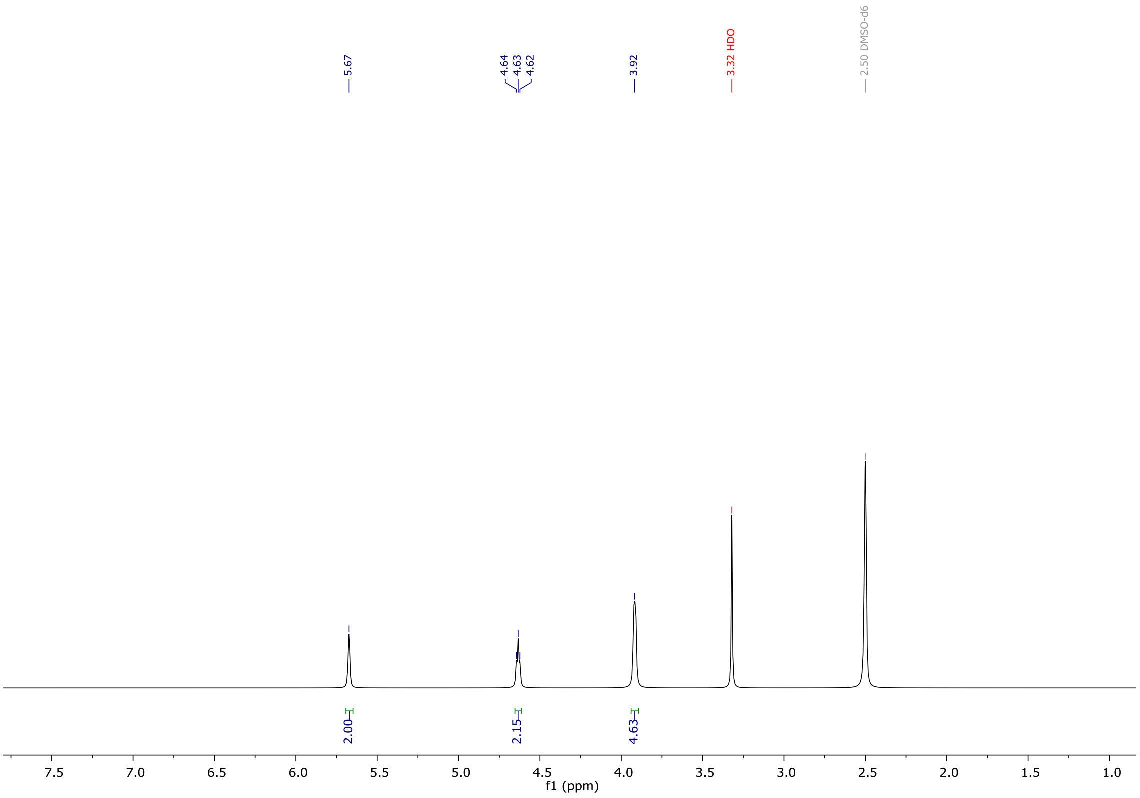

^{13}C NMR (126 MHz, $CDCl_3$)

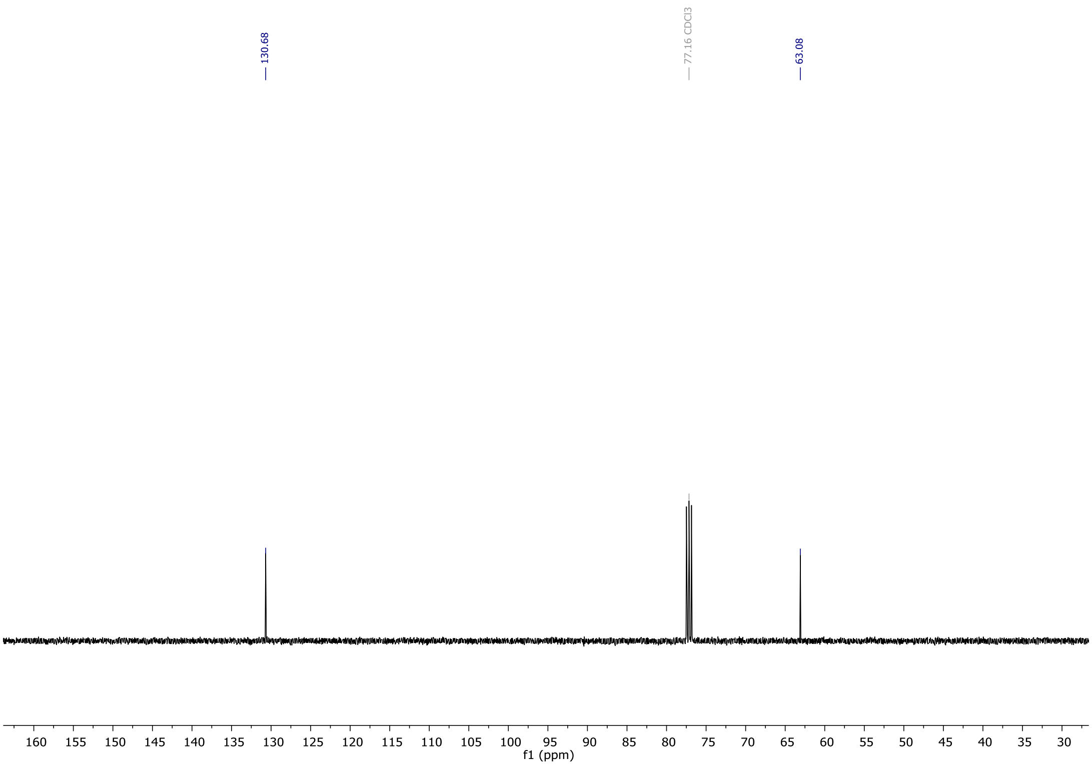

^{1}H NMR (500 MHz, DMSO-d_6)

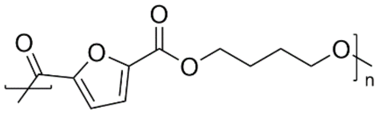

4a

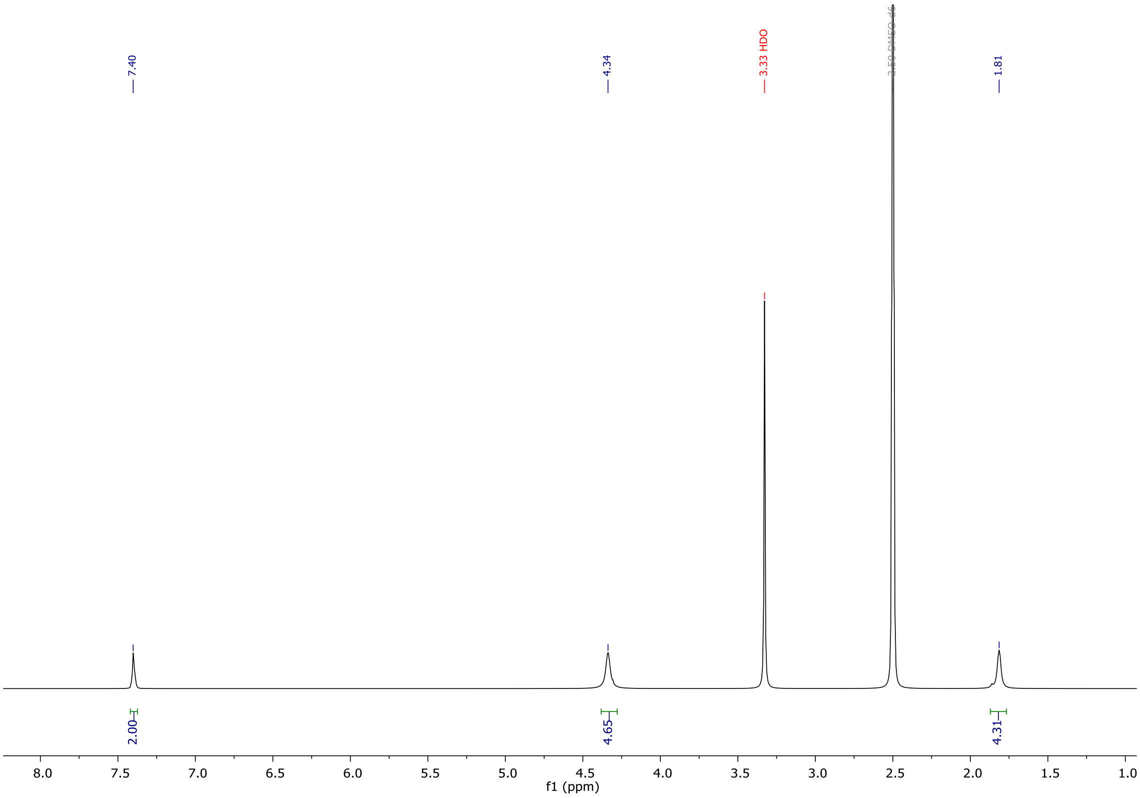

^{1}H NMR (500 MHz, DMSO-d_6)

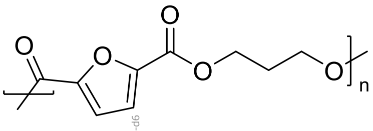

4b

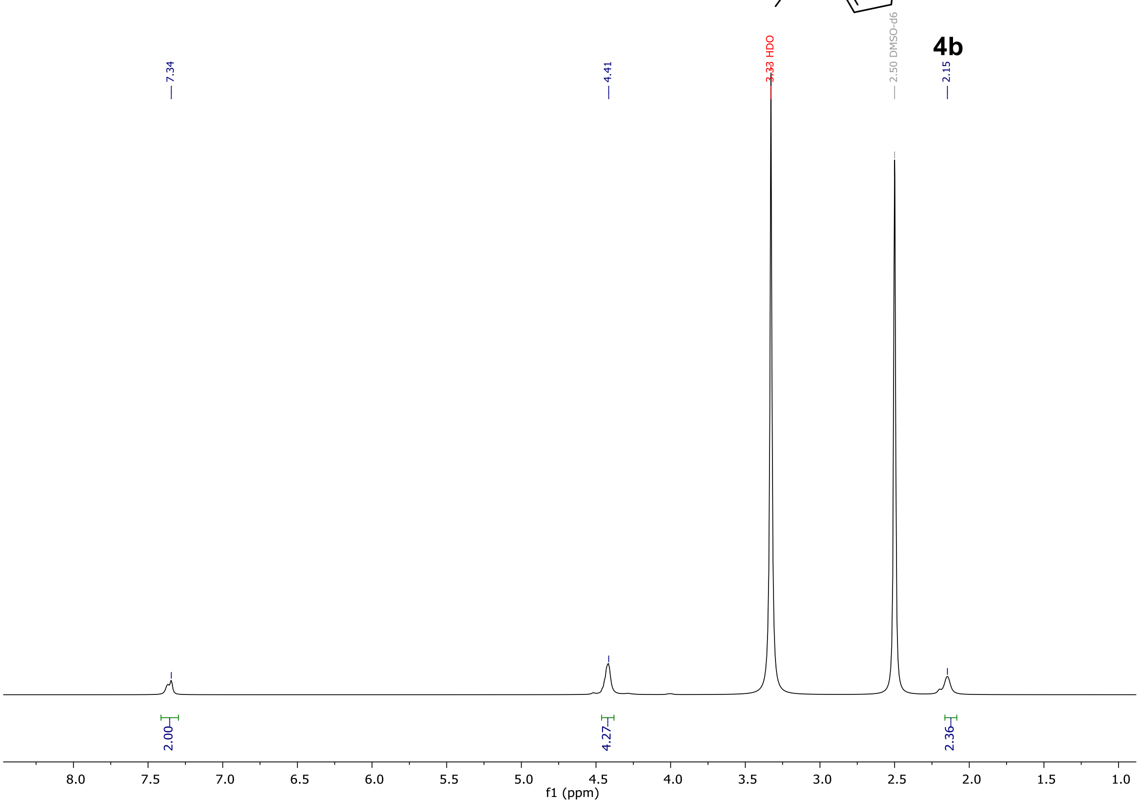

^{1}H NMR (500 MHz, DMSO-d_6)

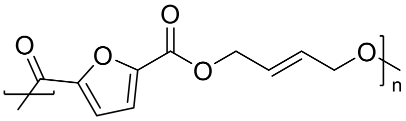

4c

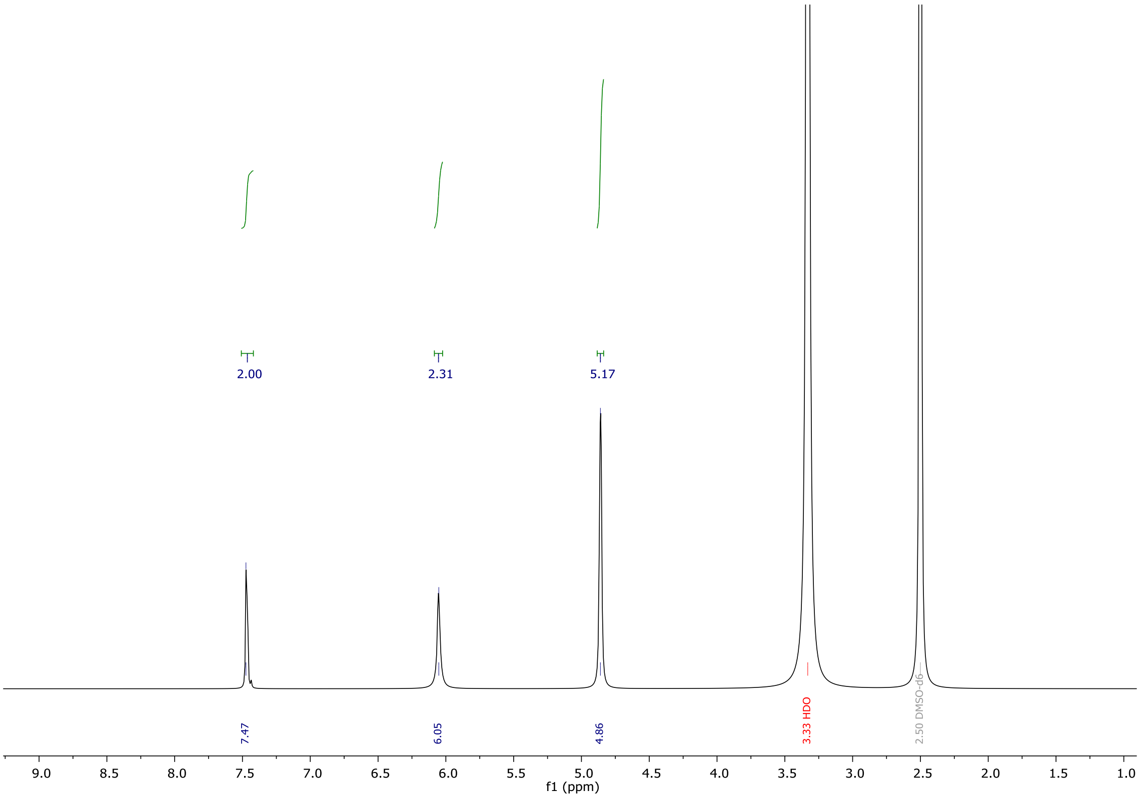

^{1}H NMR (500 MHz, DMSO-d_6)

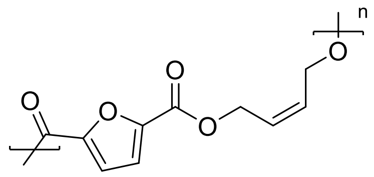

4d

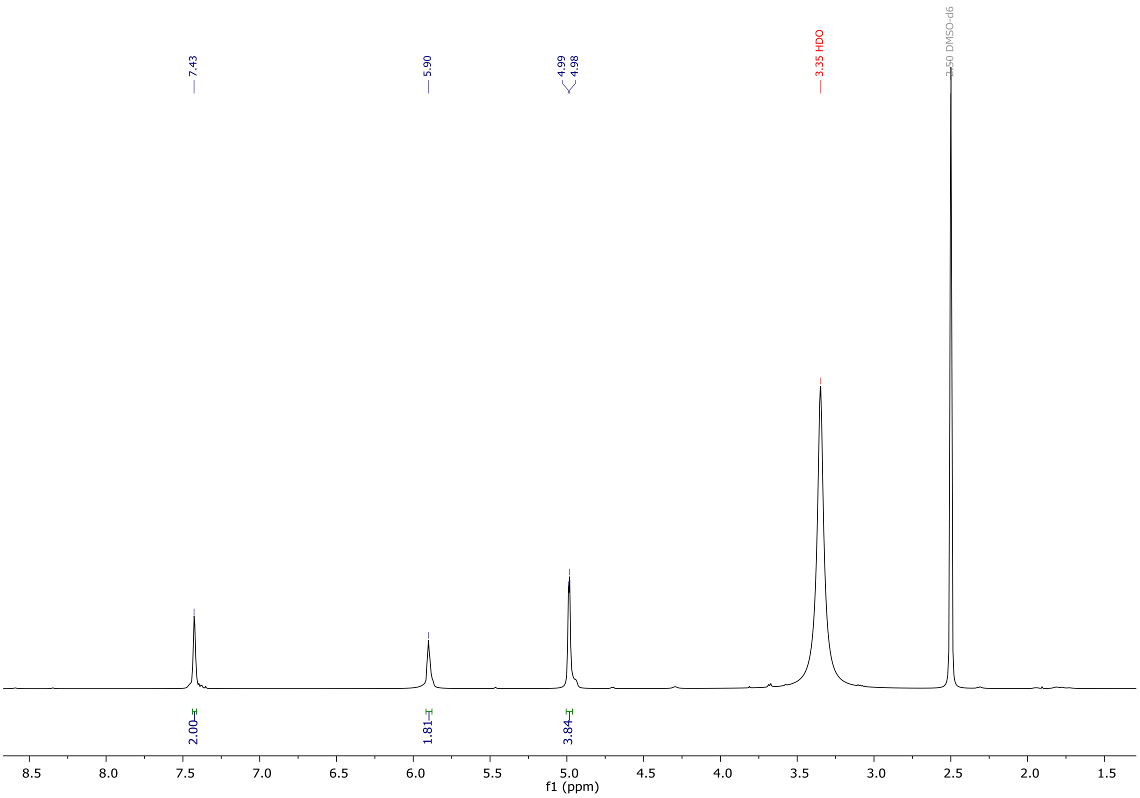

¹H NMR (500 MHz, DMSO-d_6)

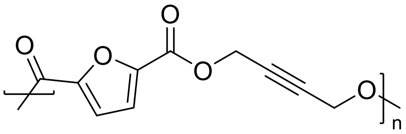

4e

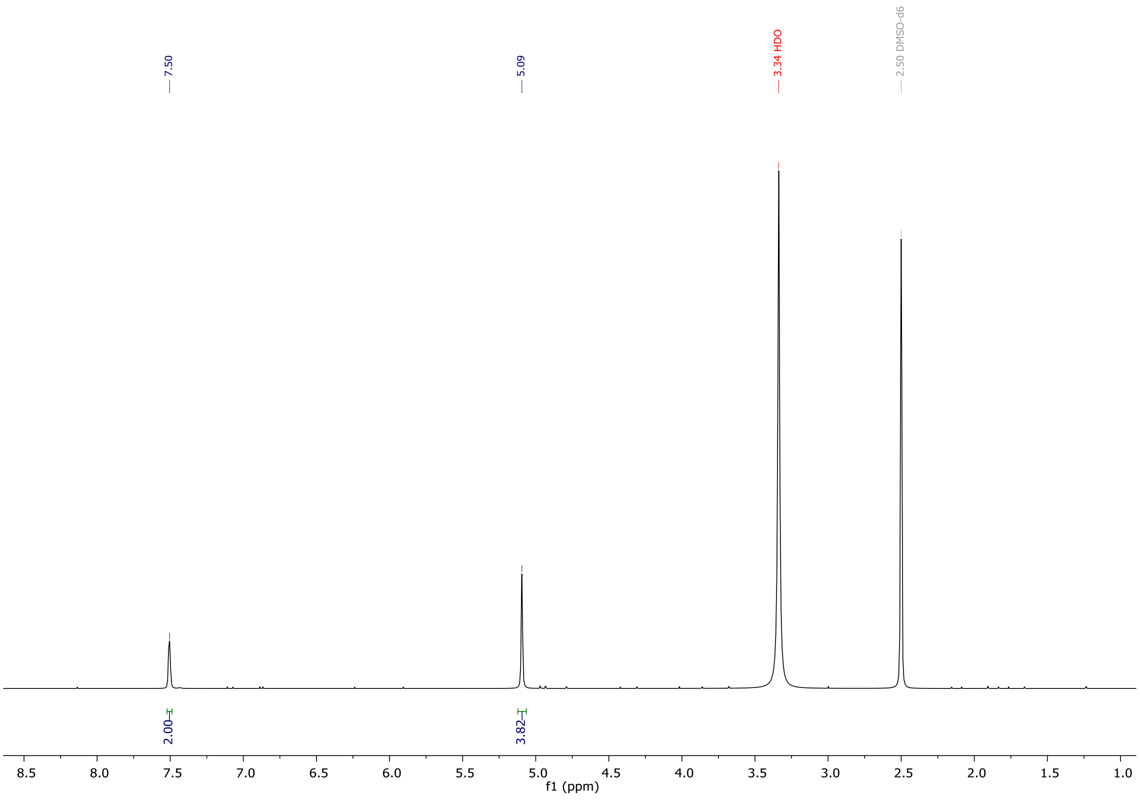

^{1}H NMR (500 MHz, DMSO-d_6)

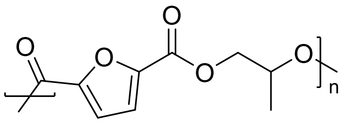

4f

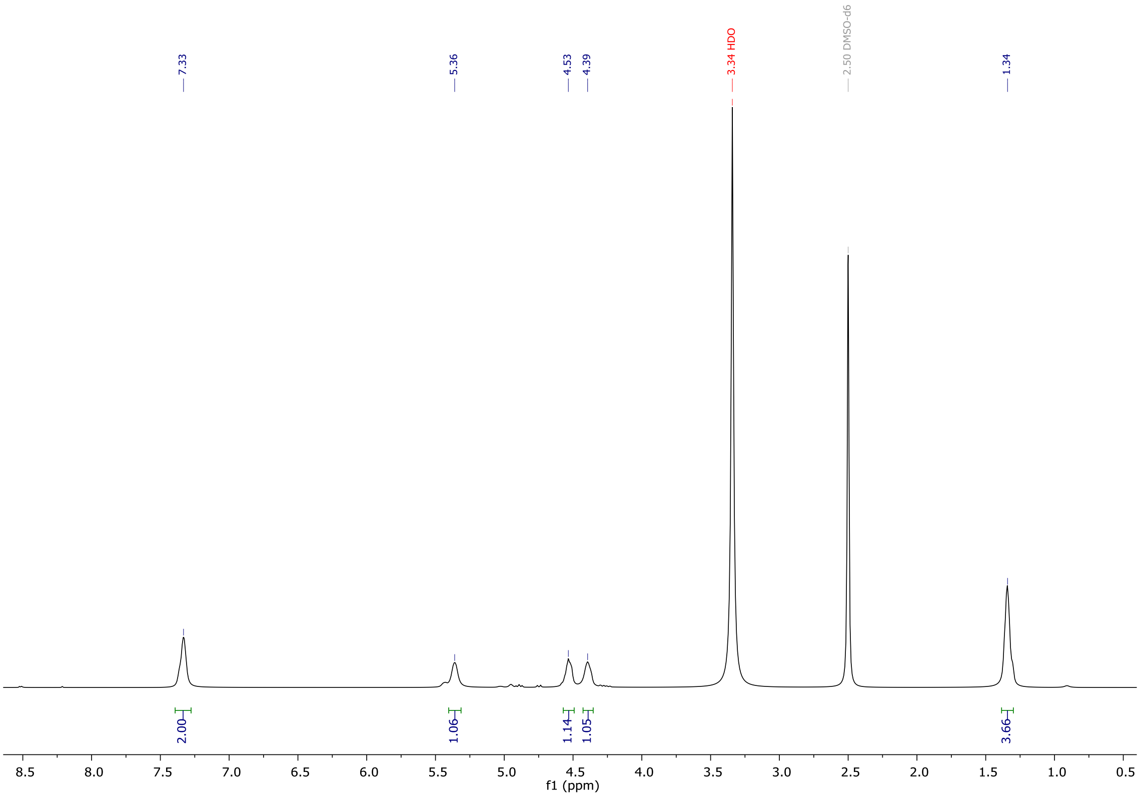

^{1}H NMR (500 MHz, DMSO-d_6)

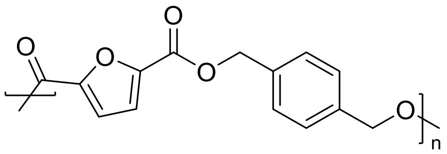

4g

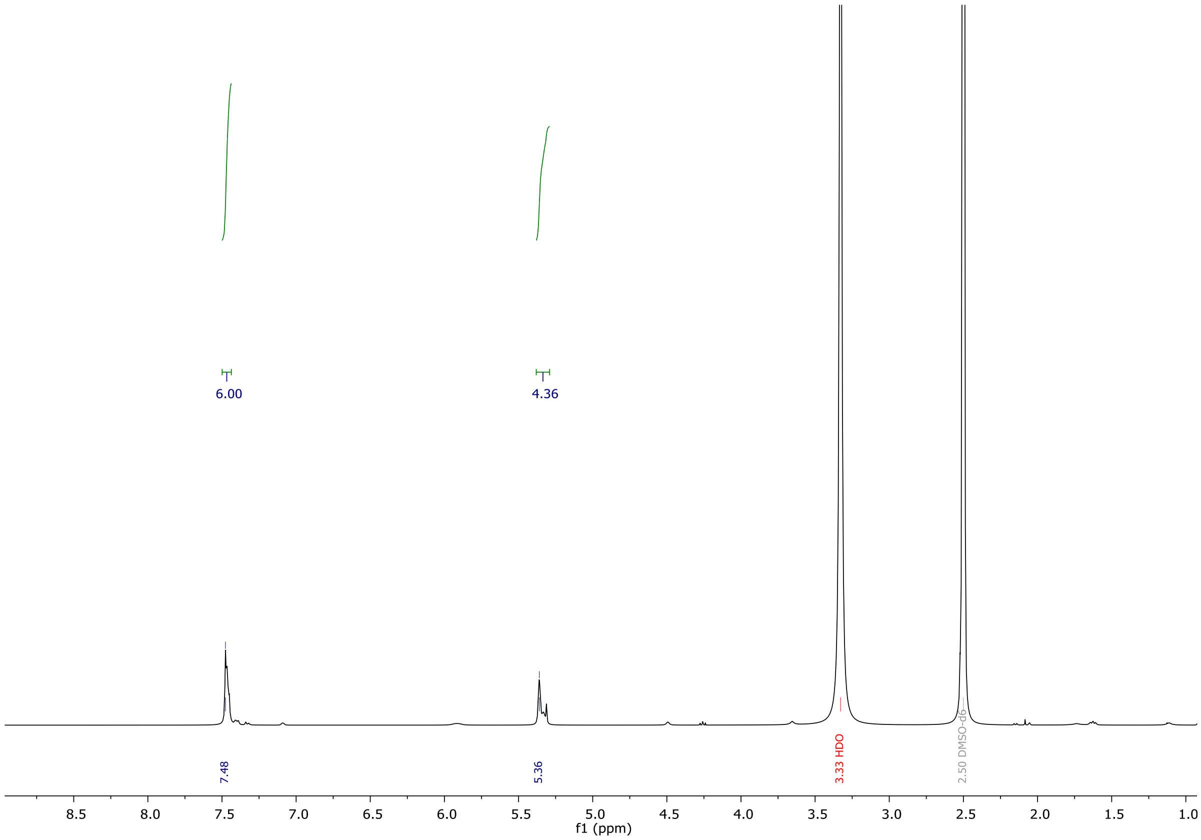

^{1}H NMR (500 MHz, DMSO-d_6)

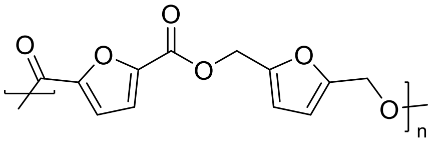

4h

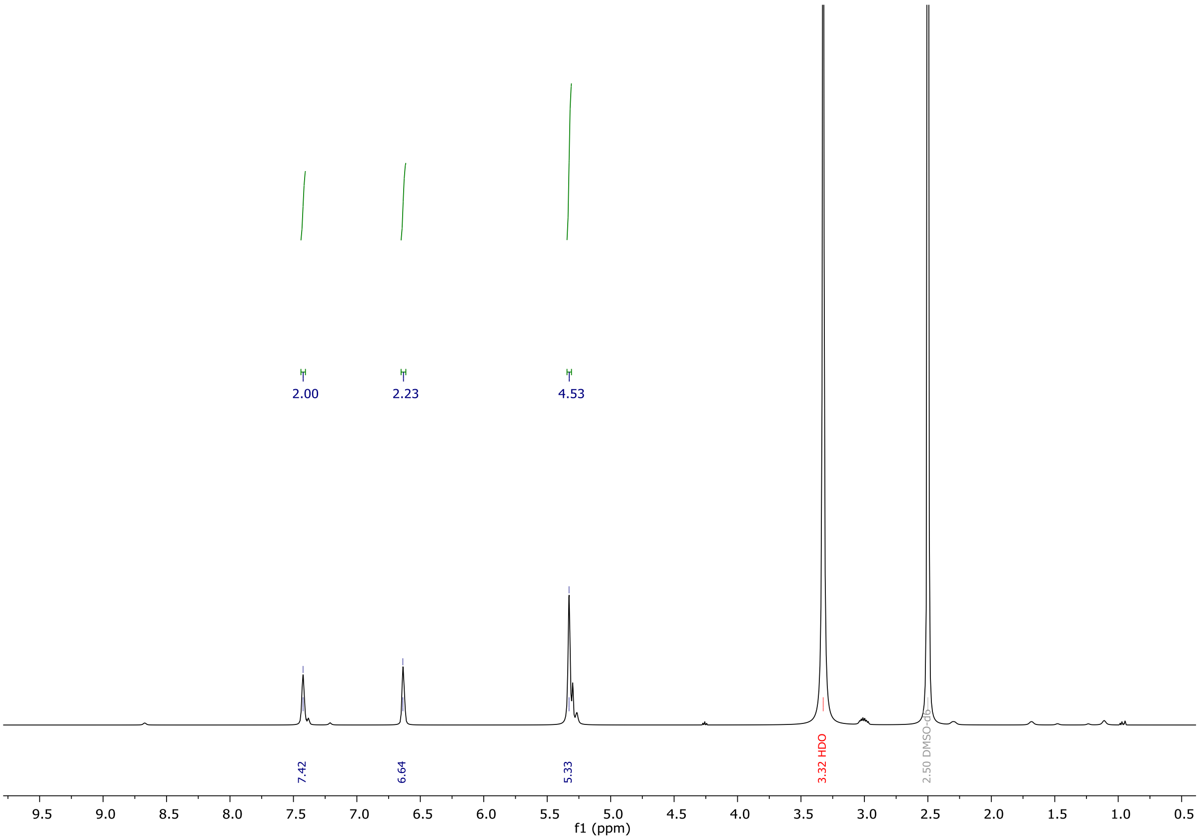

^{1}H NMR (500 MHz, DMSO-d_6)

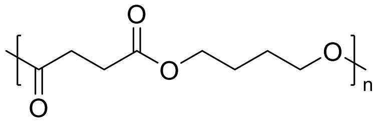

5a

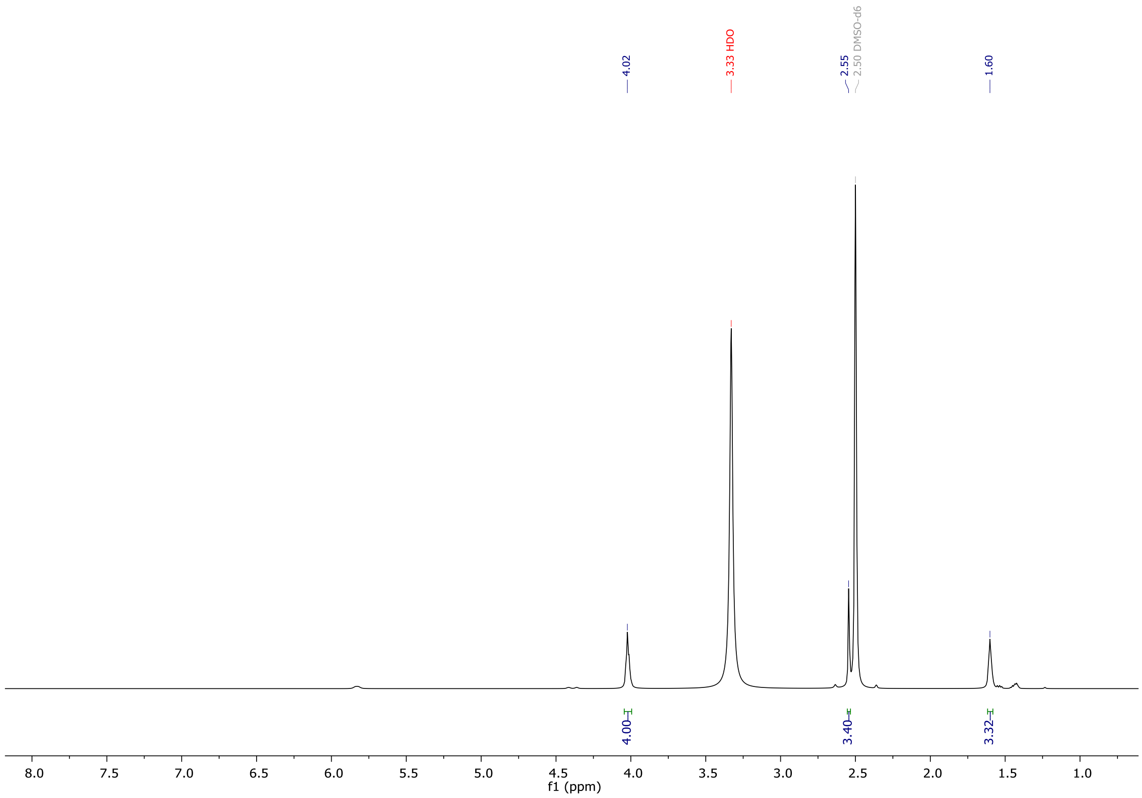

[1]**H NMR** (500 MHz, DMSO-*d*$_6$)

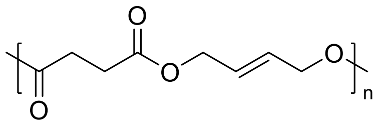

5c

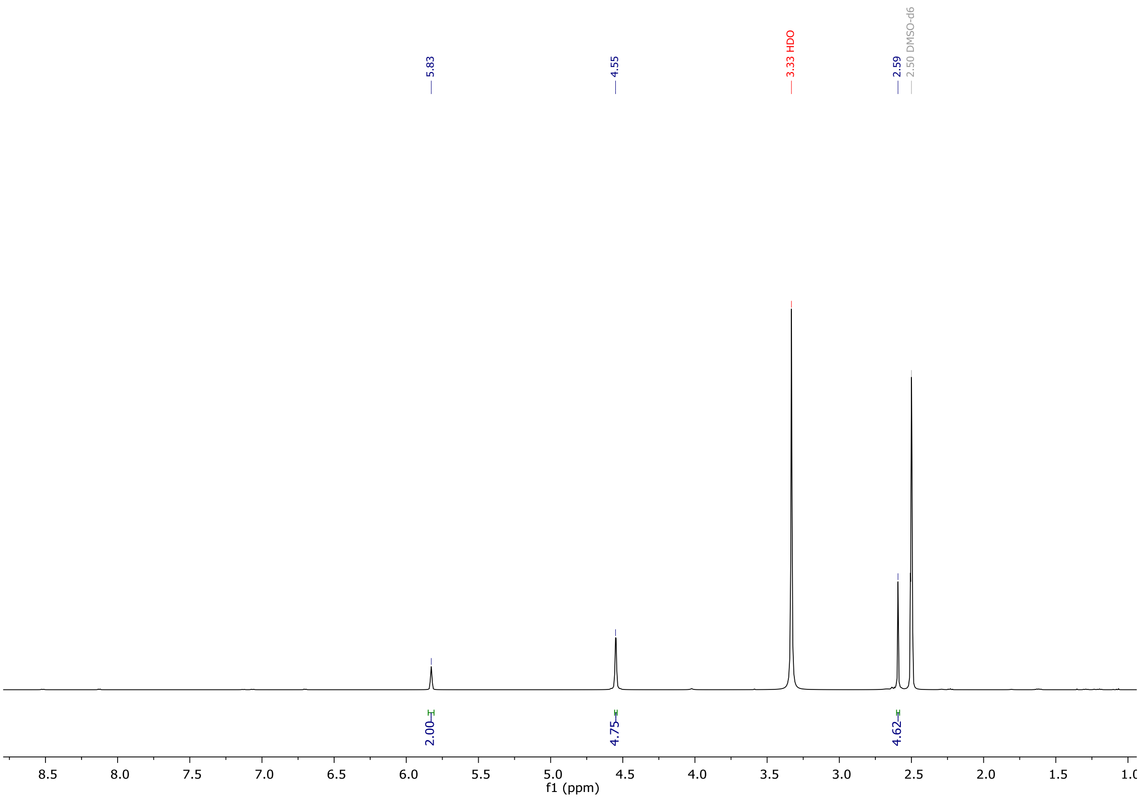

^{1}H NMR (500 MHz, DMSO-d_6)

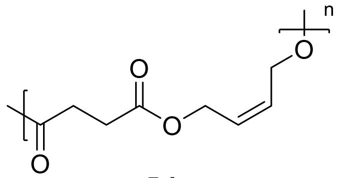

5d

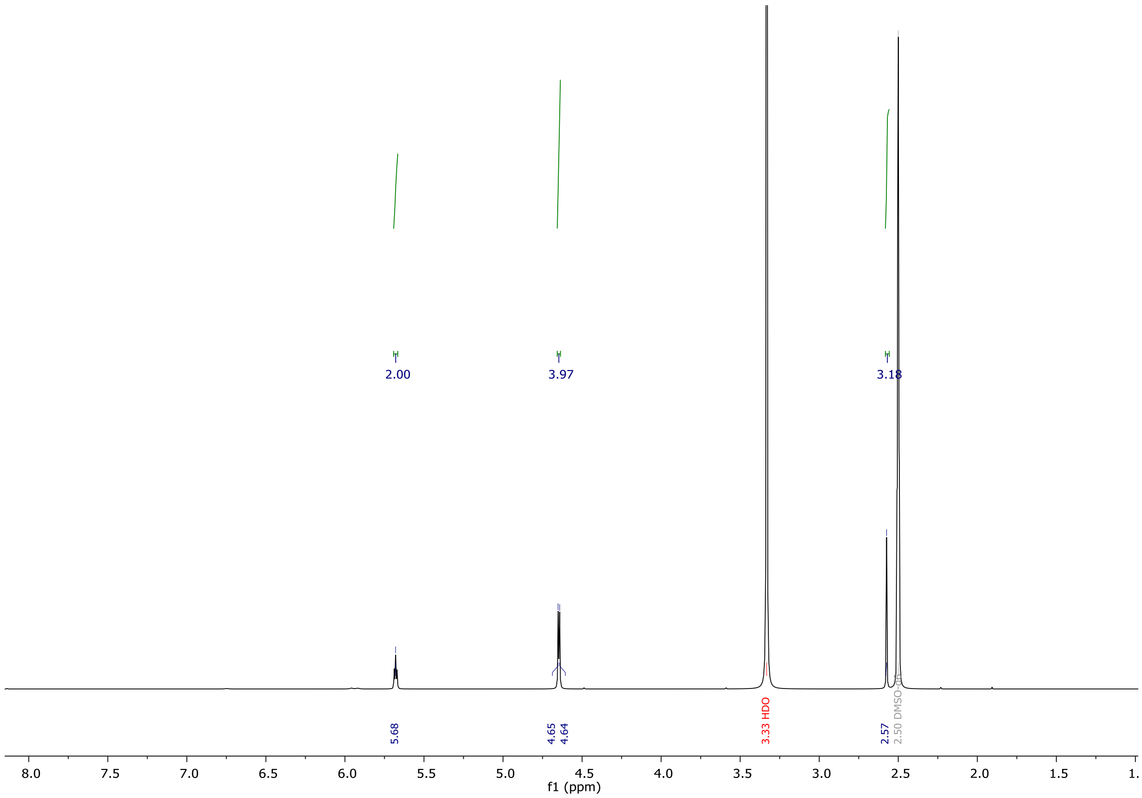

^{1}H NMR (500 MHz, DMSO-d_6)

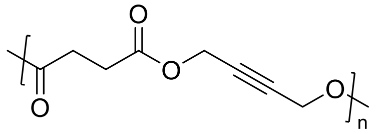

5e

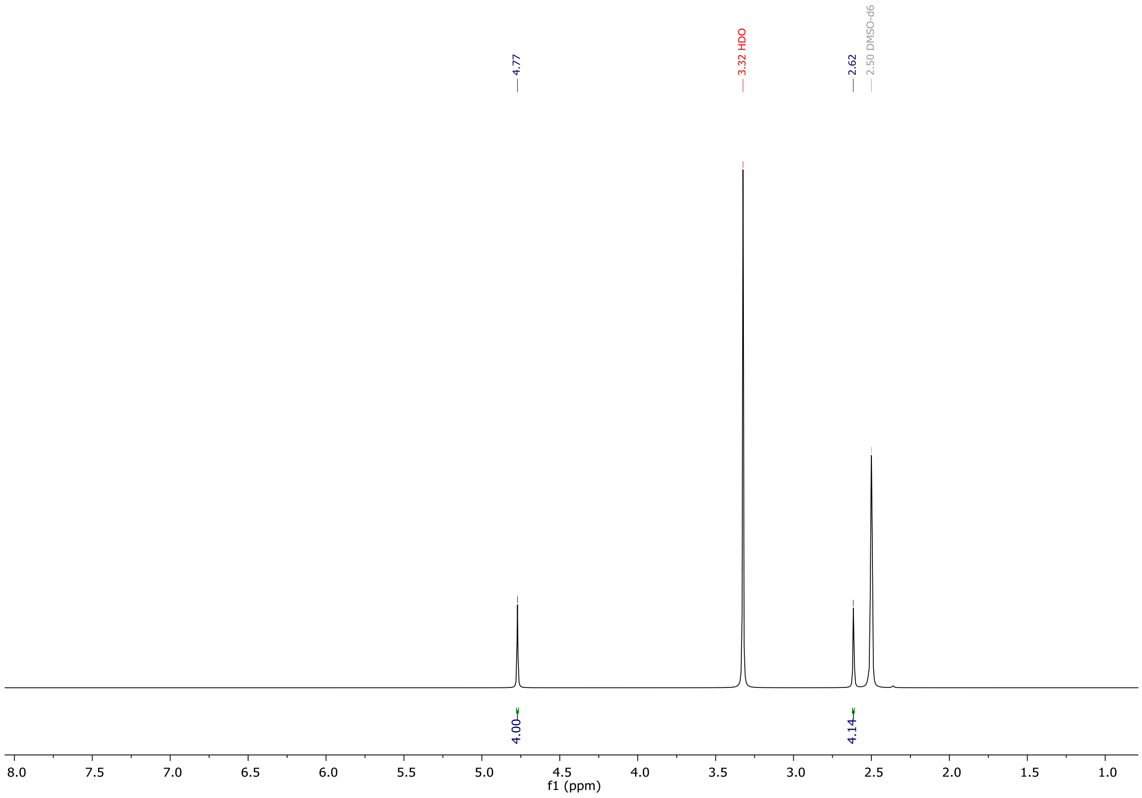

^{1}H NMR (500 MHz, DMSO-d_6)

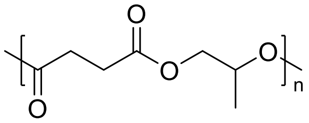

5f

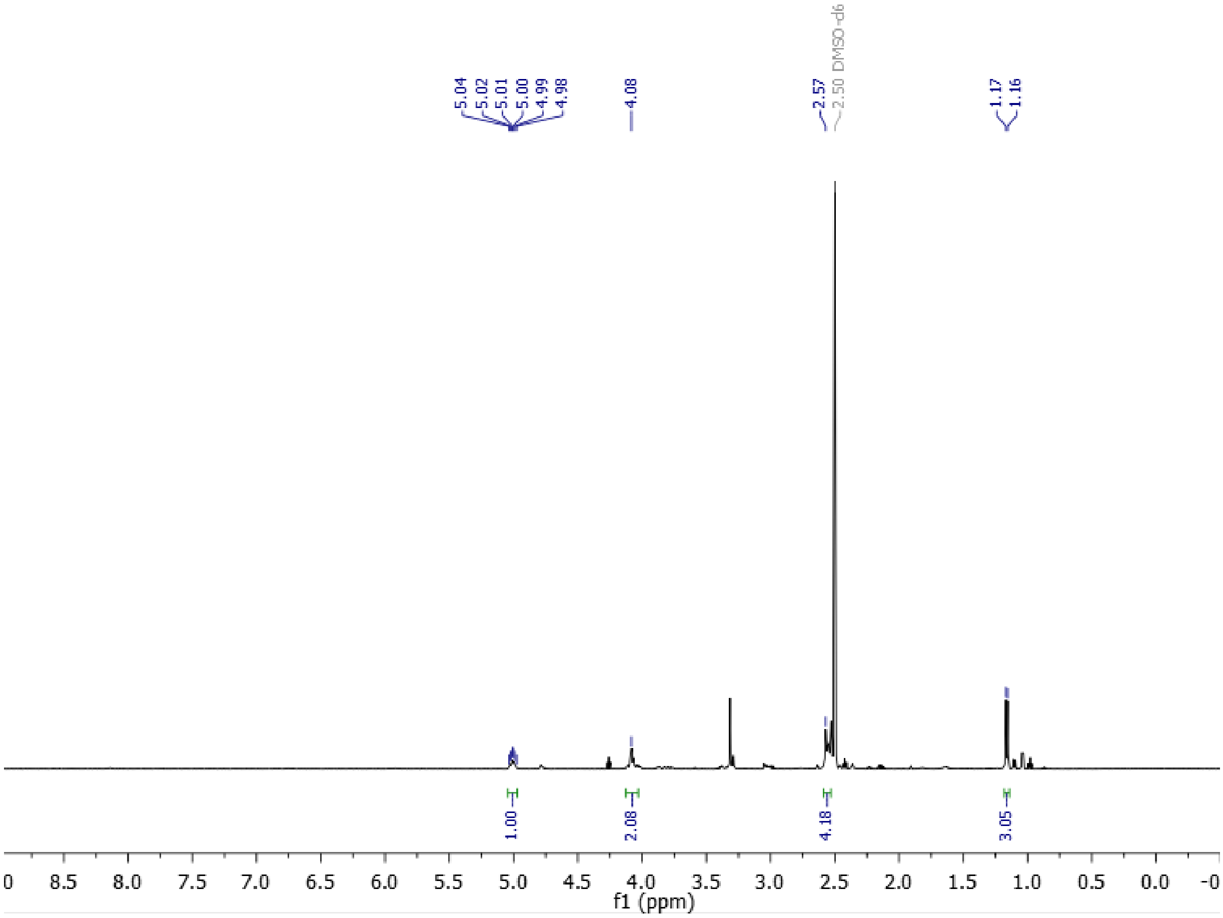

^{1}H NMR (500 MHz, DMSO-d_6)

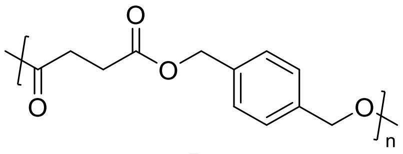

5g

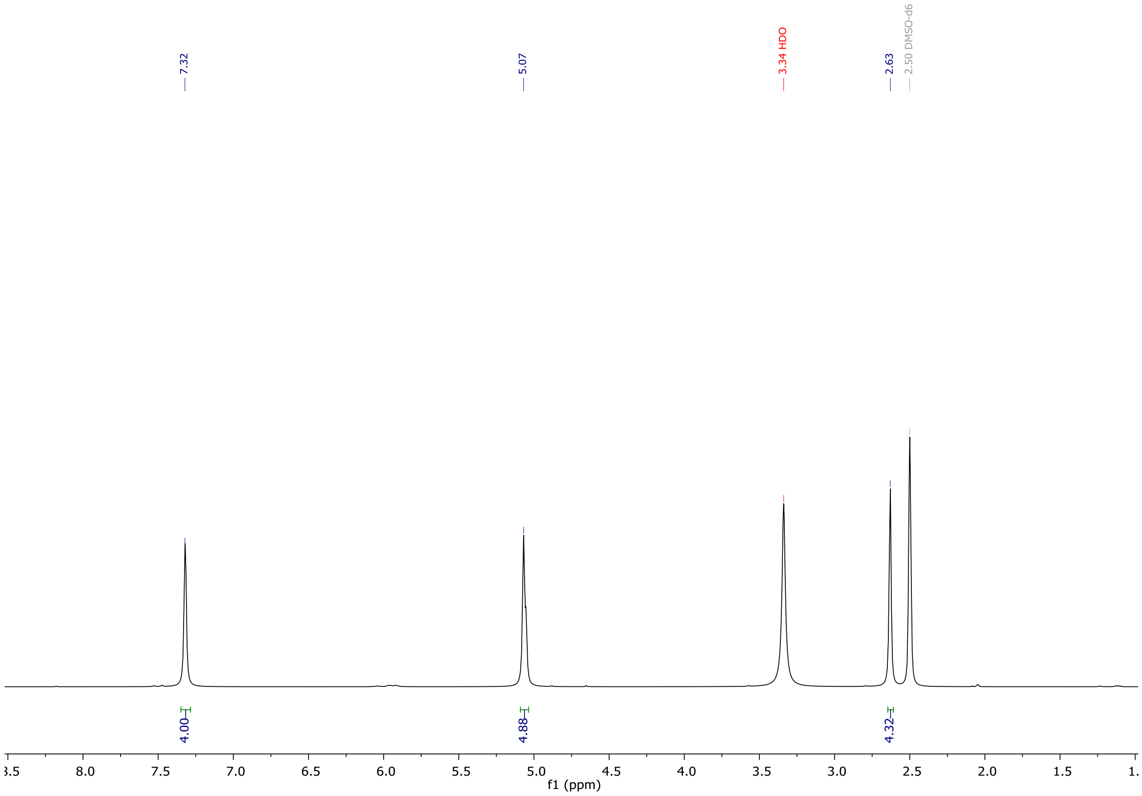

^{1}H NMR (500 MHz, DMSO-d_6)

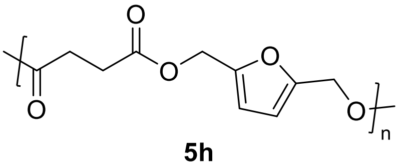

5h

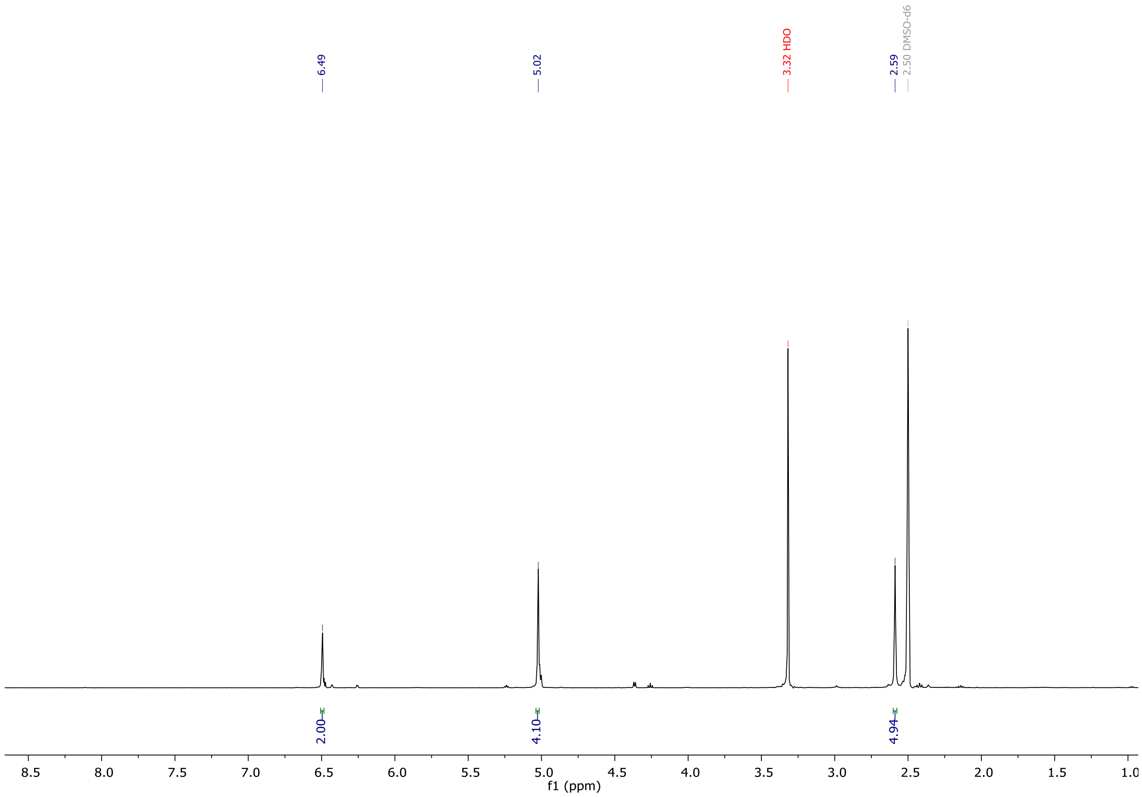

^{1}H NMR (500 MHz, DMSO-d_6)

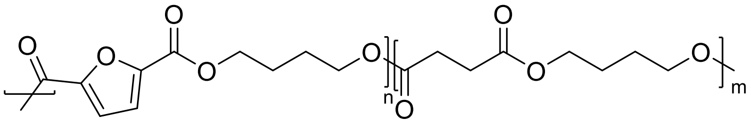

6a

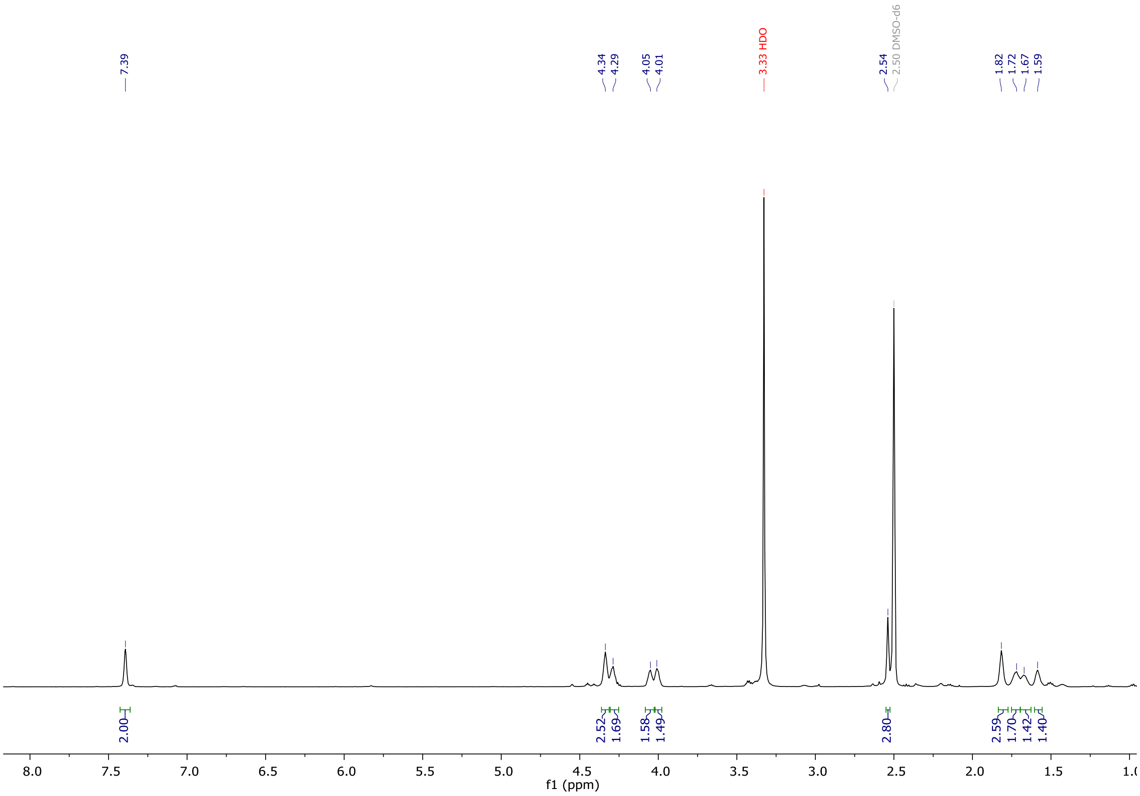

^{1}H NMR (500 MHz, DMSO-d_6)

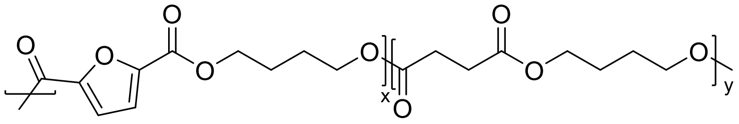

6a'

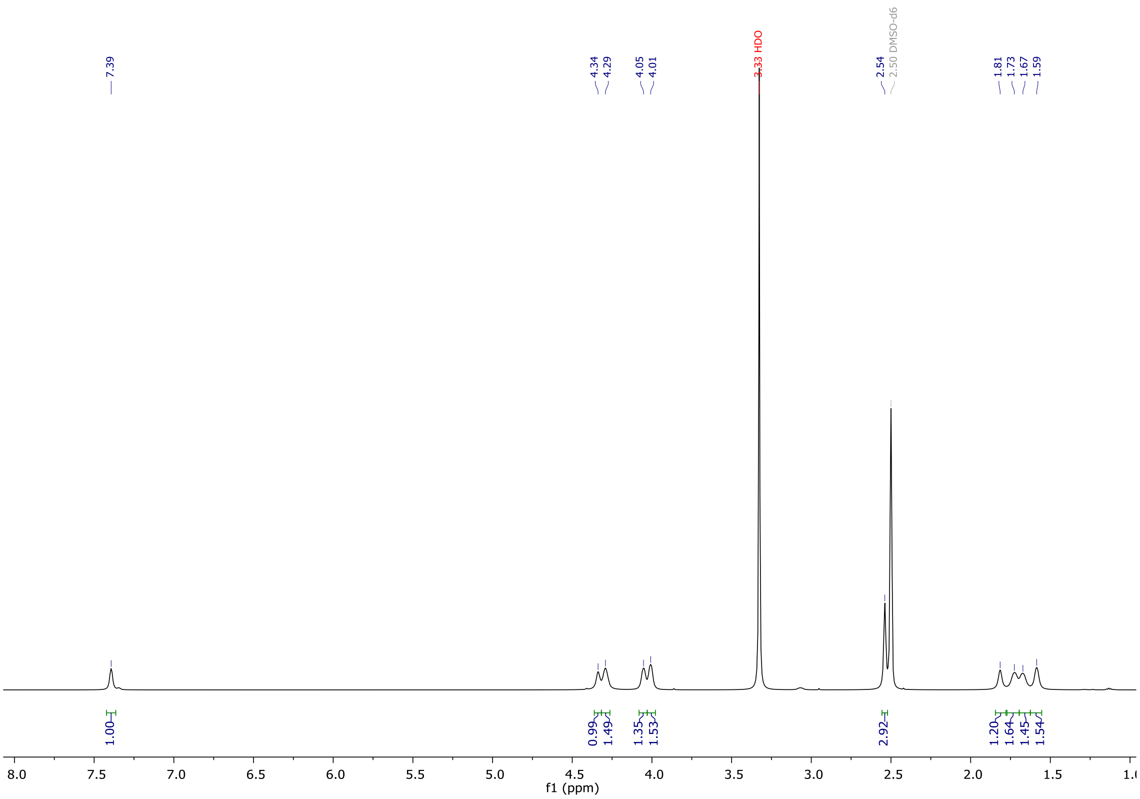

¹H NMR (500 MHz, DMSO-d_6)

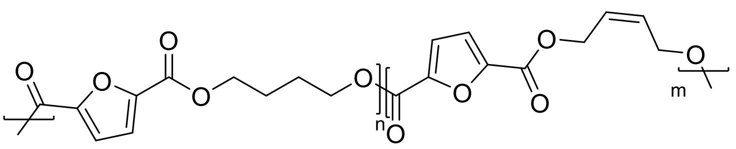

7a

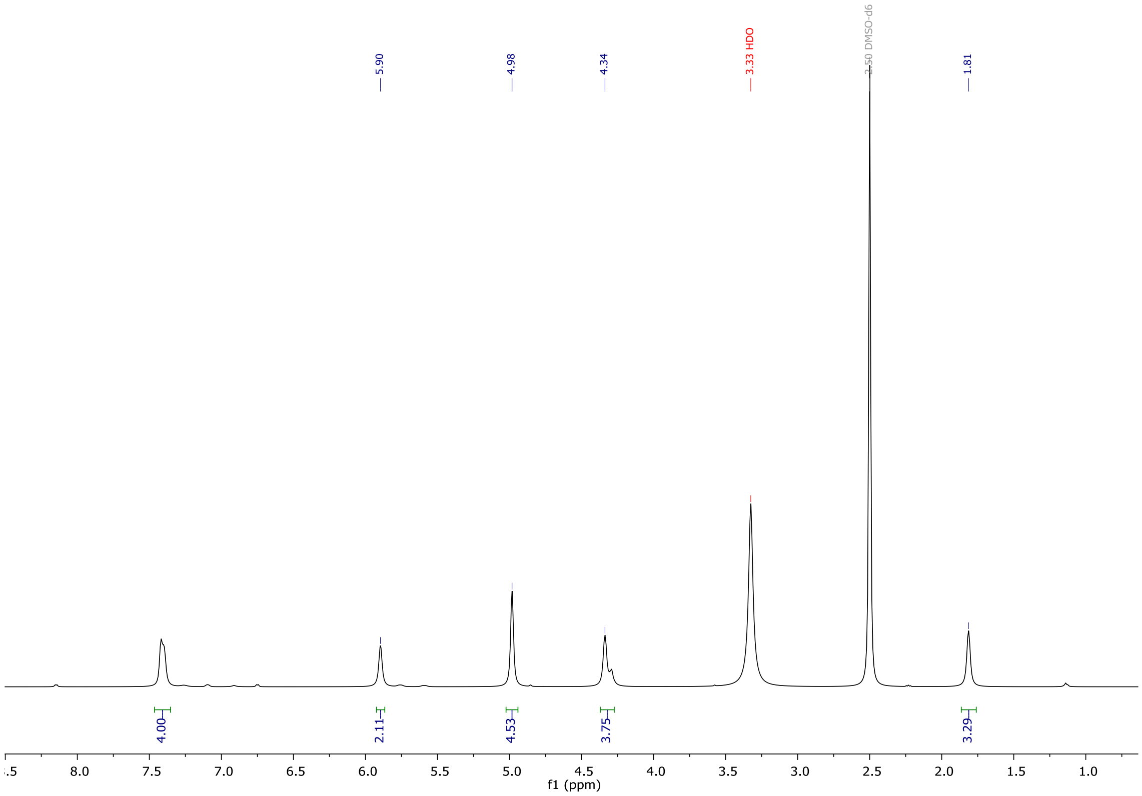

^{1}H NMR (500 MHz, DMSO-d_6)

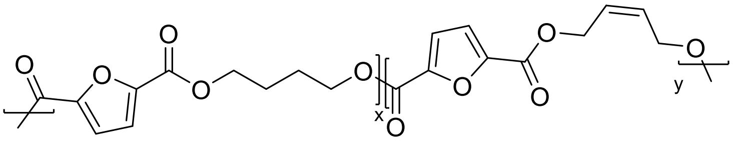

7a'

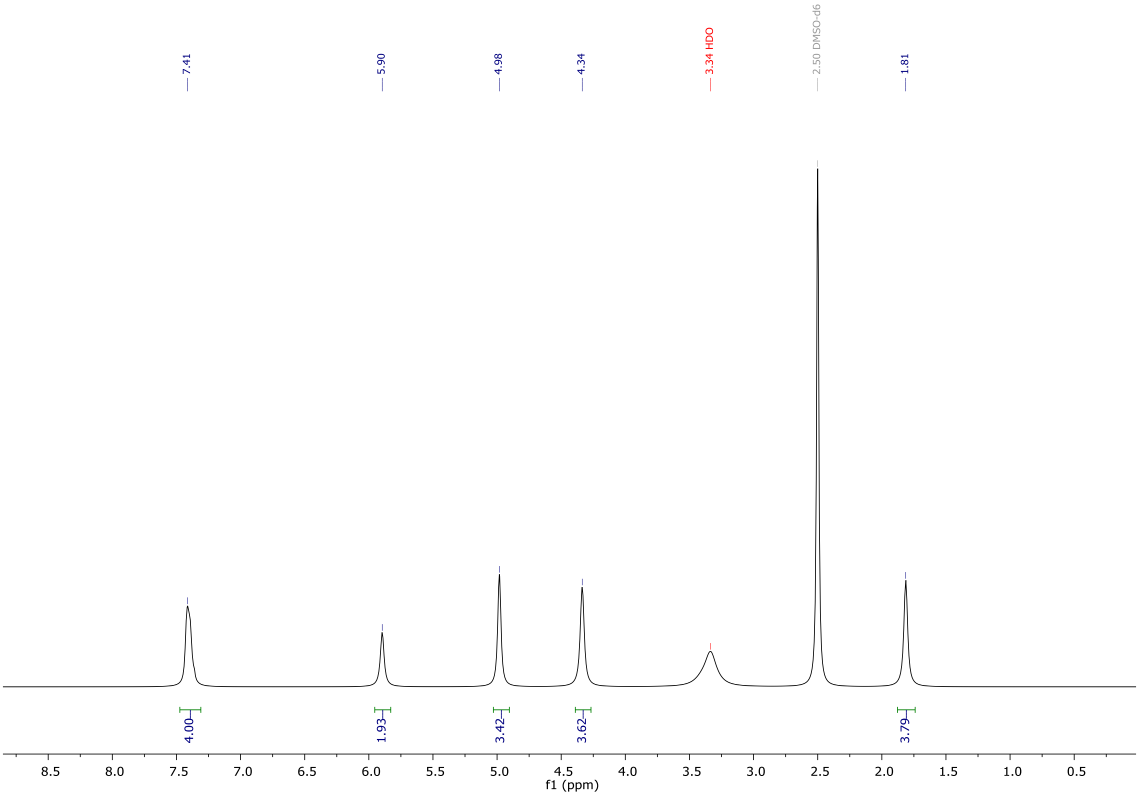

^{1}H NMR (500 MHz, DMSO-d_6)

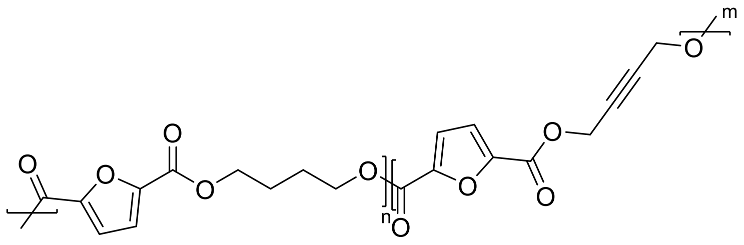

7b

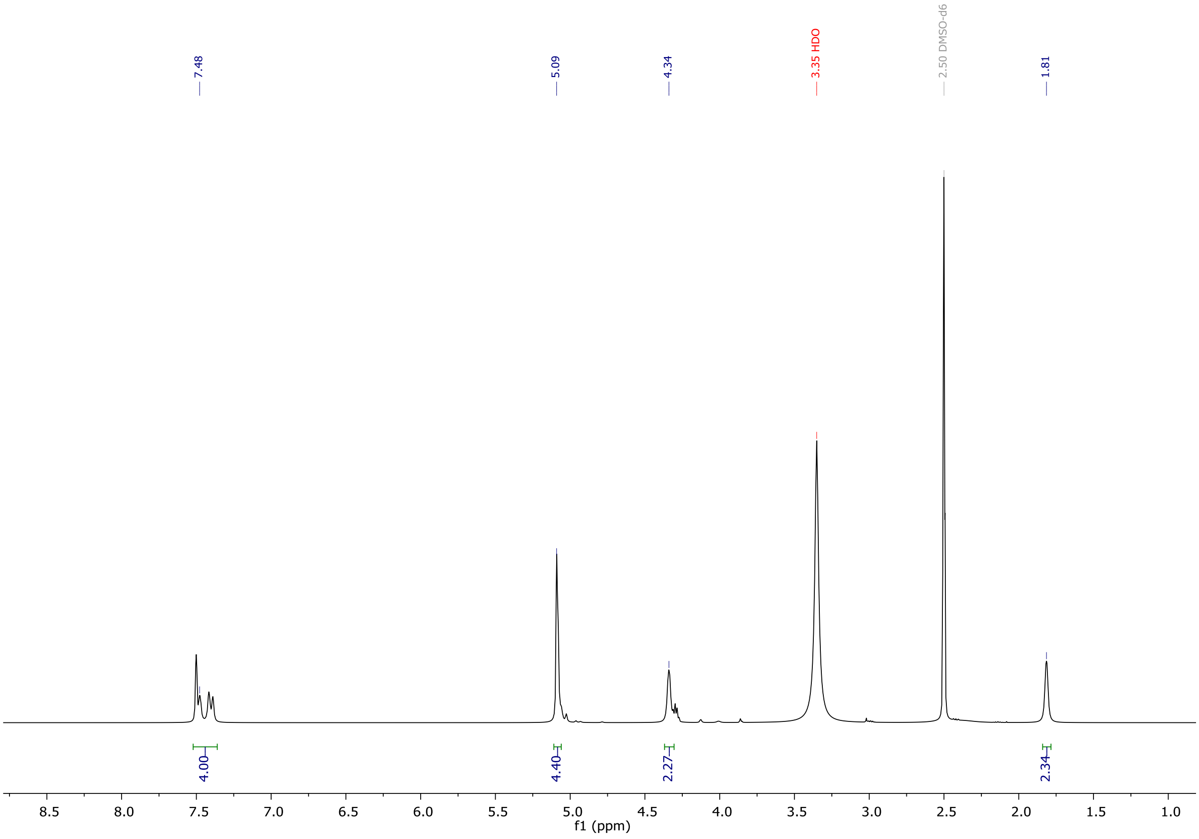

^{1}H NMR (500 MHz, DMSO-d_6)

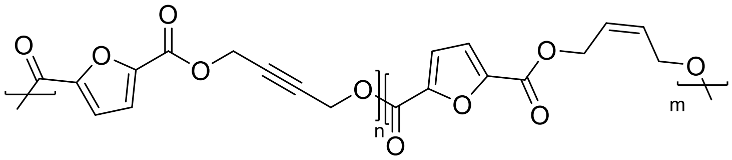

7c

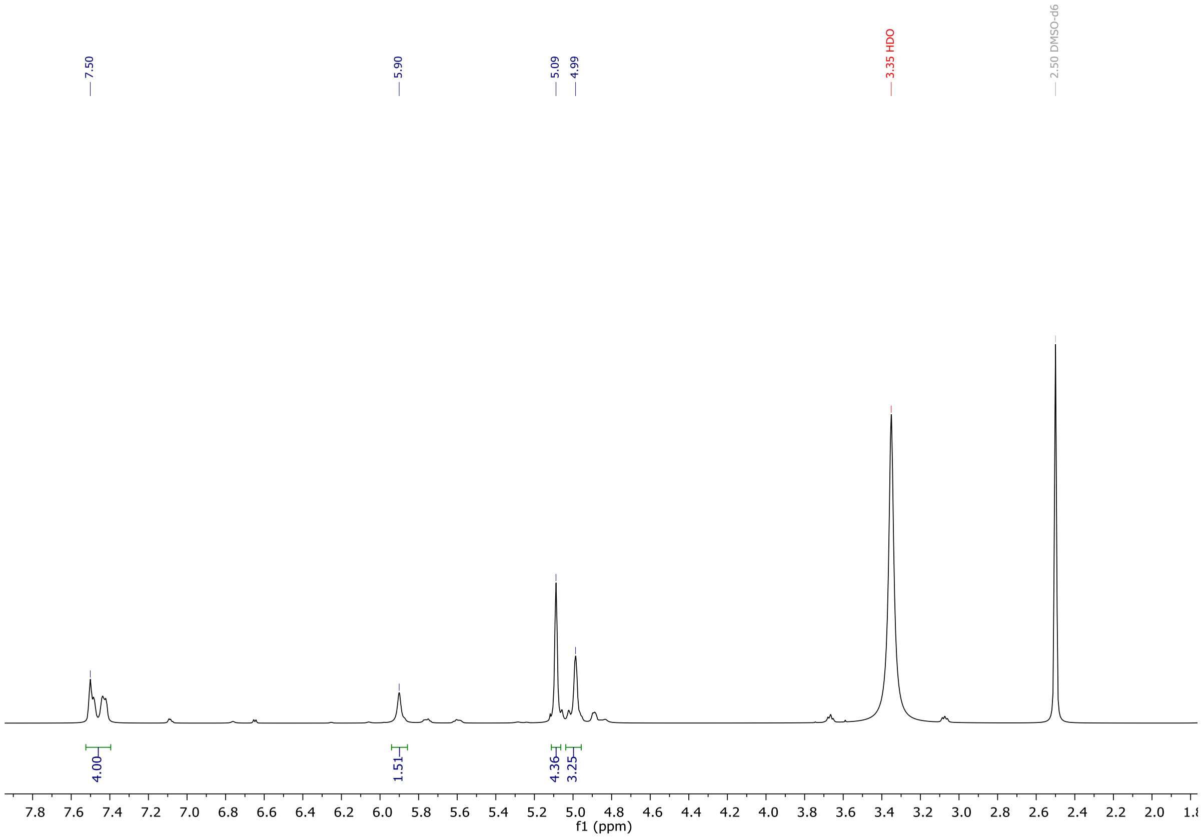

DSC: Individual thermograms

Legend: DSC thermograms showing first heating (blue), second heating (red), and cooling (green).

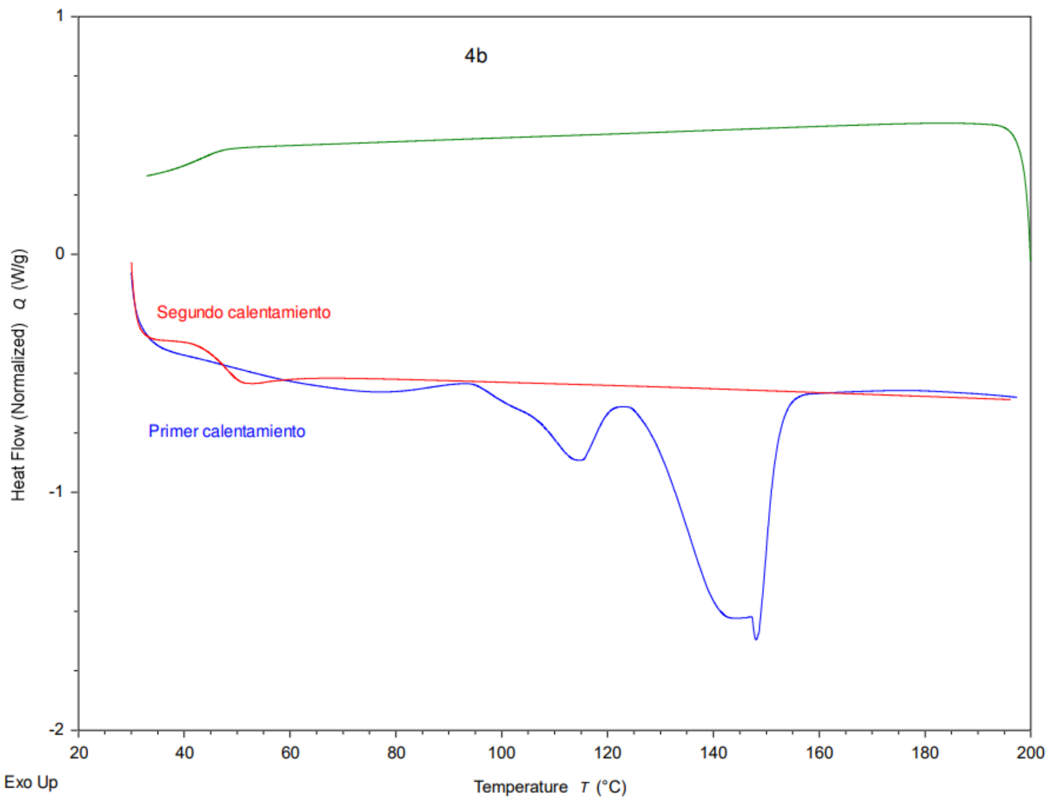

Figure S1. DSC scan of **4b**.

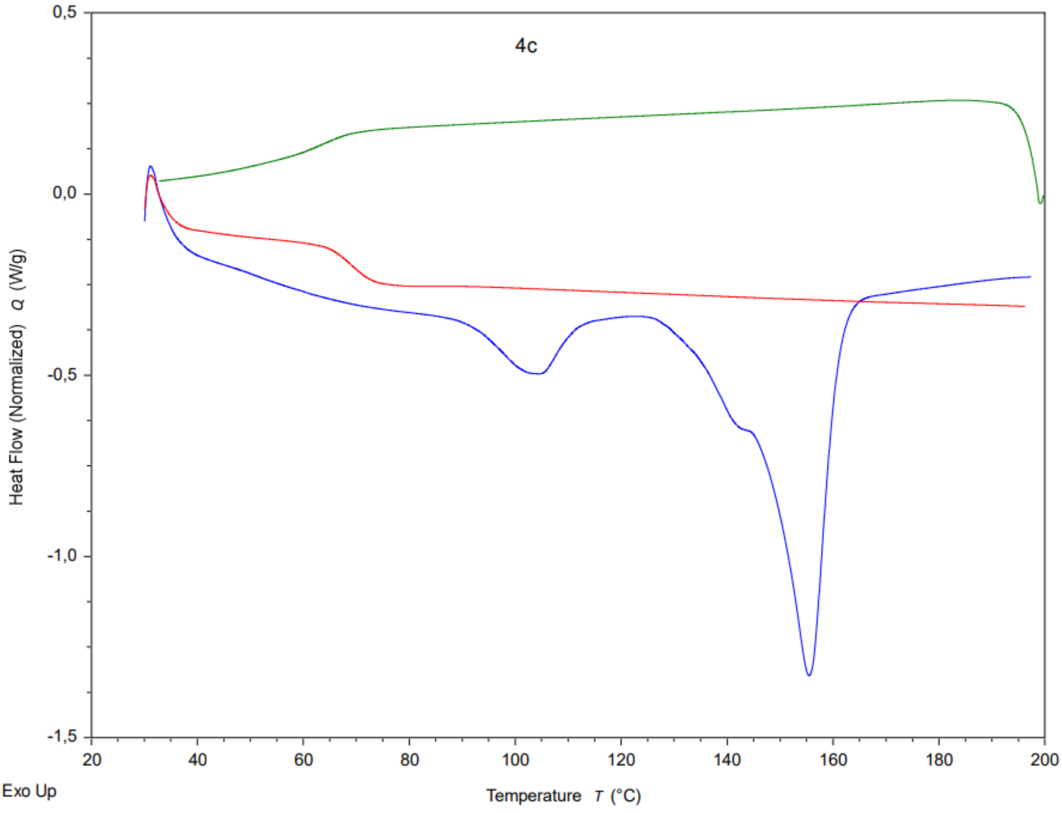

Figure S2. DSC scan of **4c**.

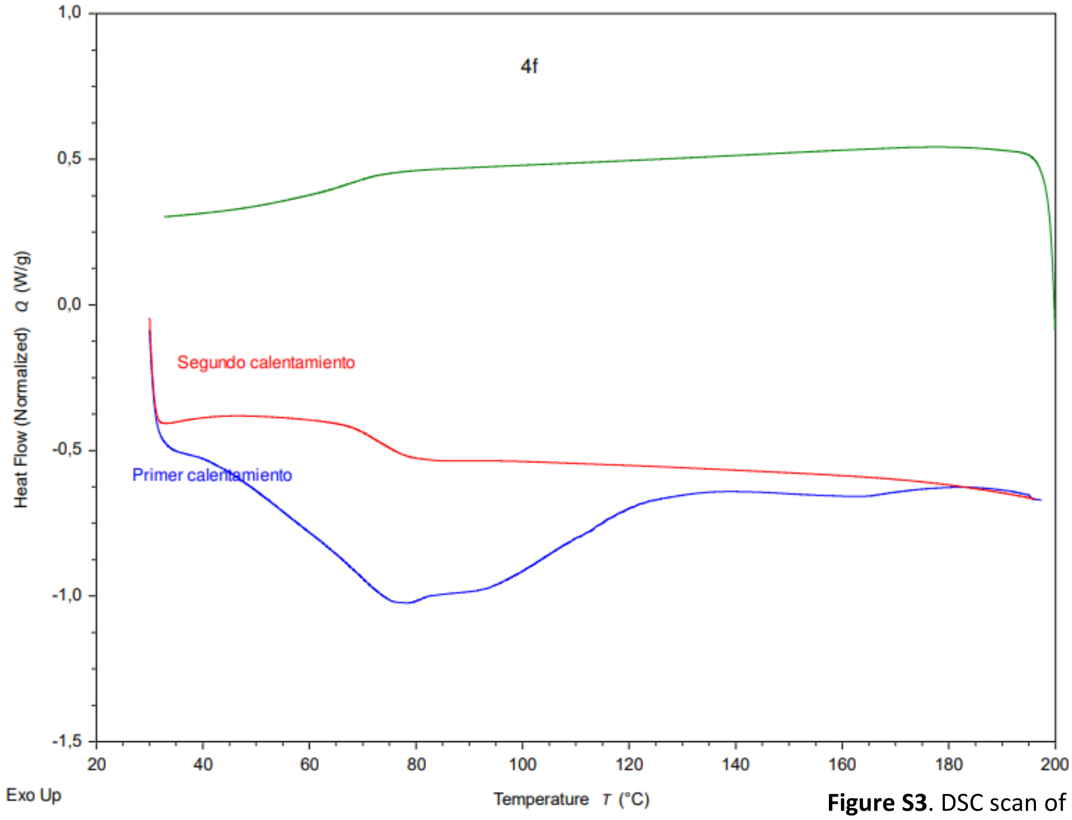

Figure S3. DSC scan of **4f**.

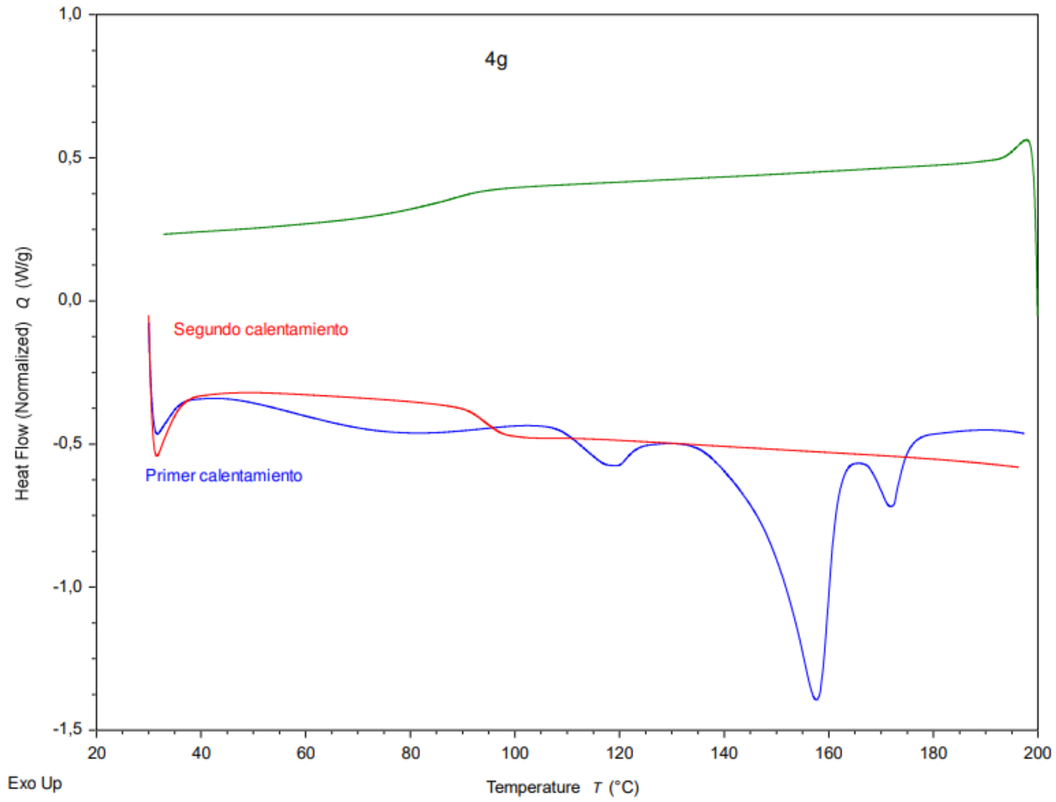

Figure S4. DSC scan of **4g**.

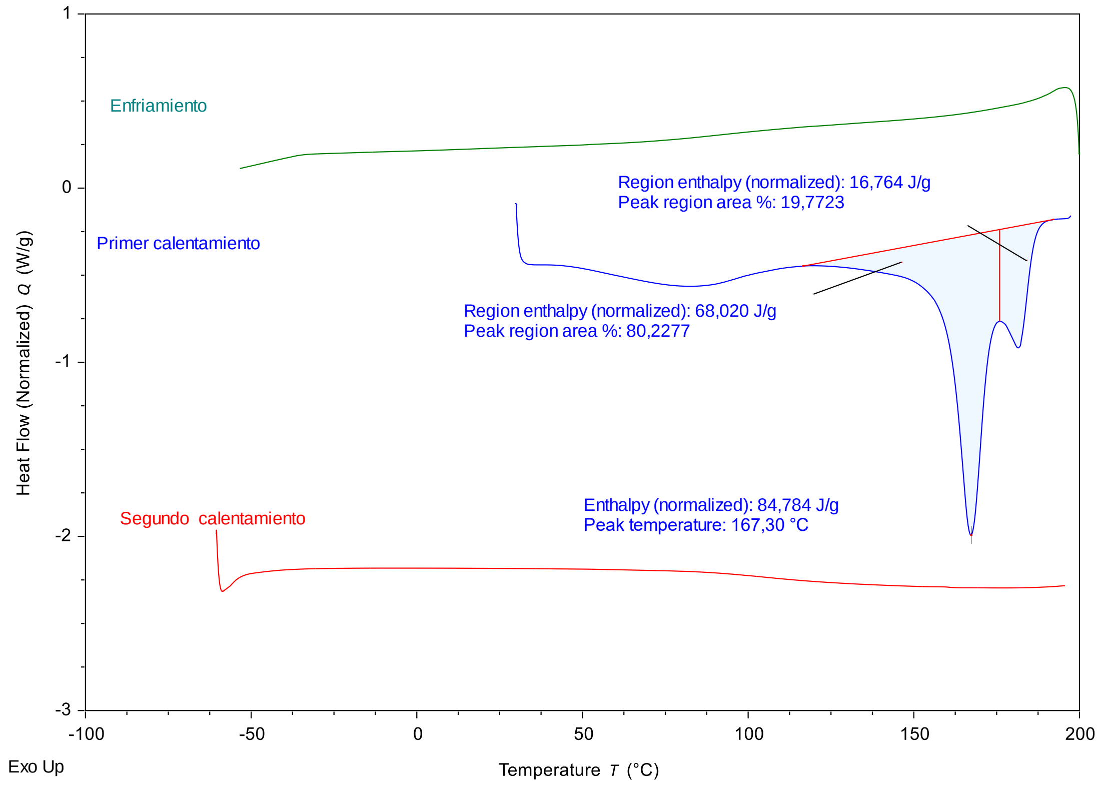

Figure S5. DSC scan of **4h**.

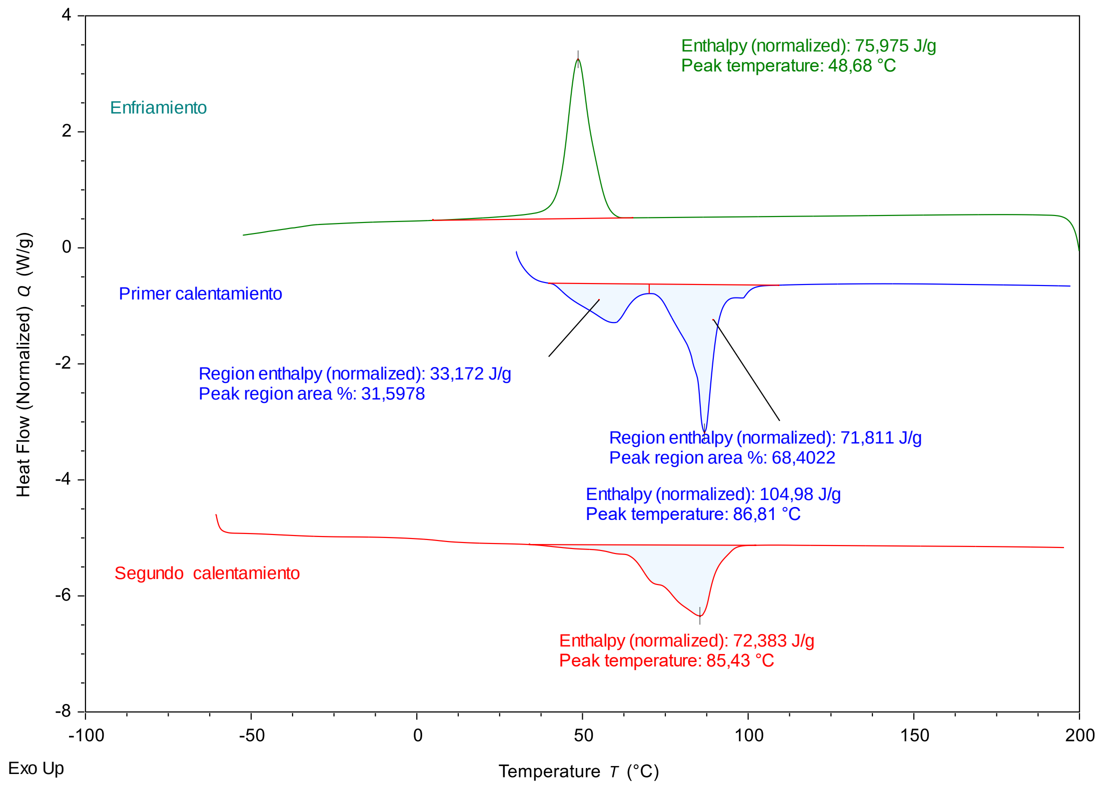

Figure S6. DSC scan of **5a**.

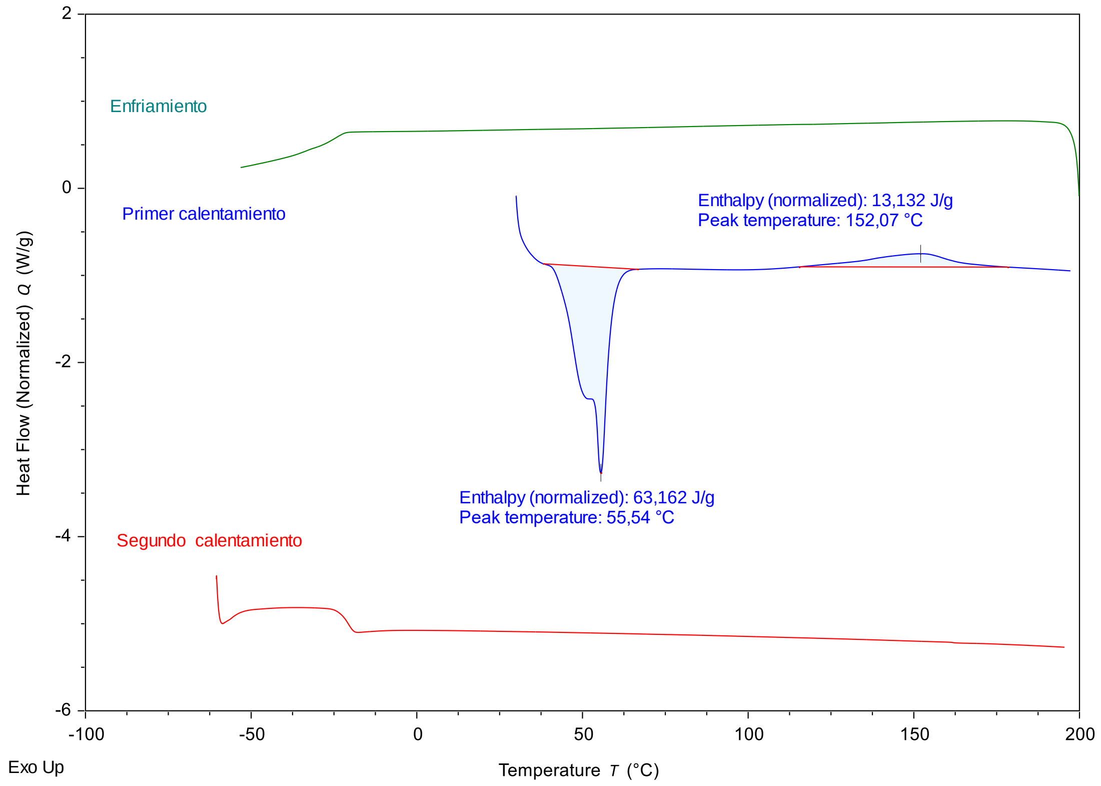

Figure S7. DSC scan of **5c**.

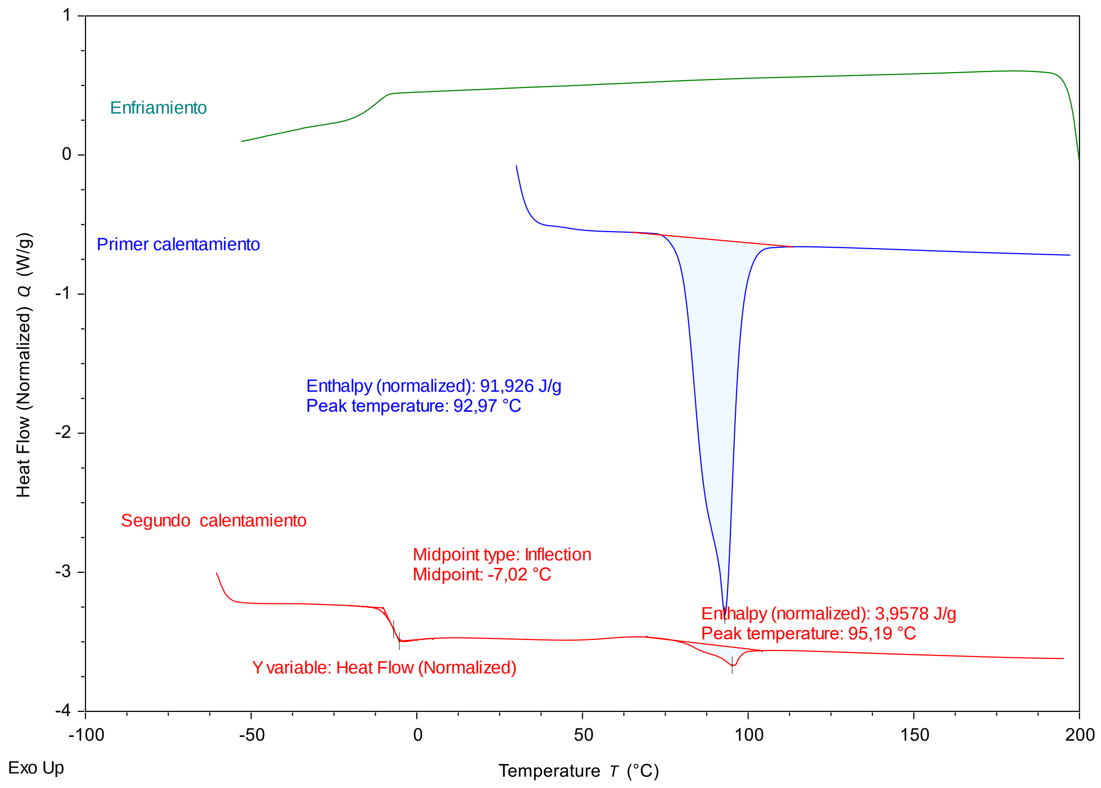

Figure S8. DSC scan of **5e**.

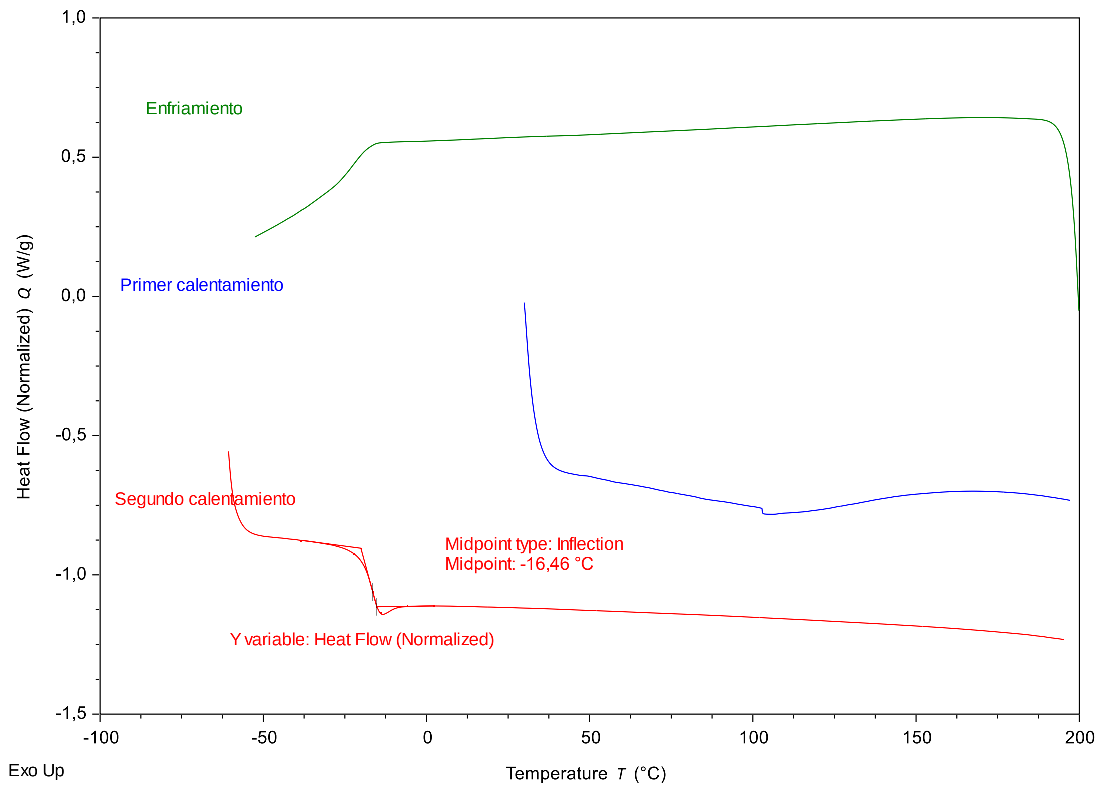

Figure S9. DSC scan of **5f**.

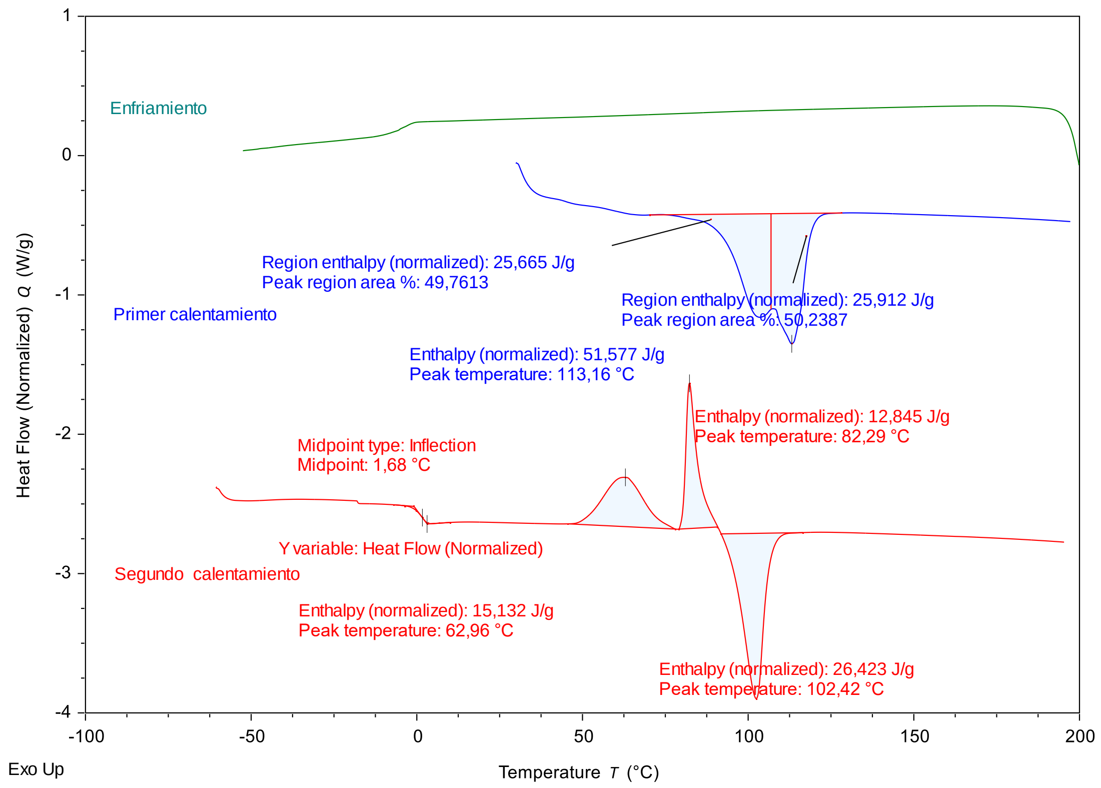

Figure S10. DSC scan of **5g**.

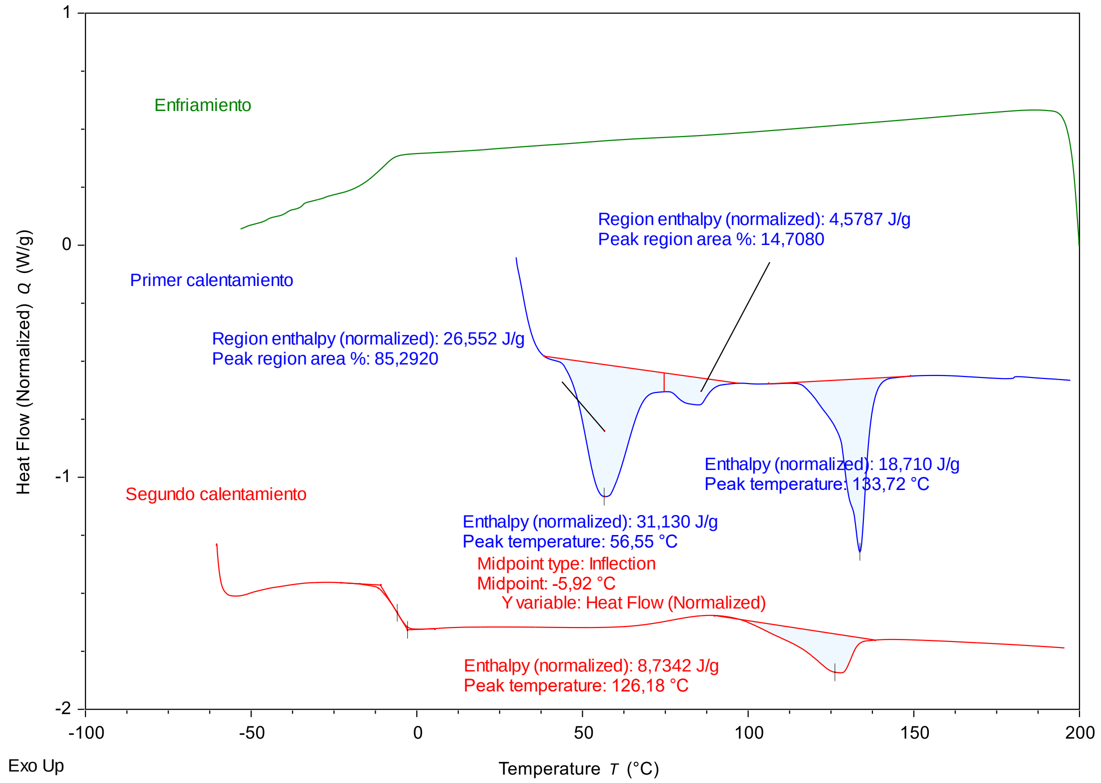

Figure S11. DSC scan of **6a**.

Thermogravimetric analysis: individual data

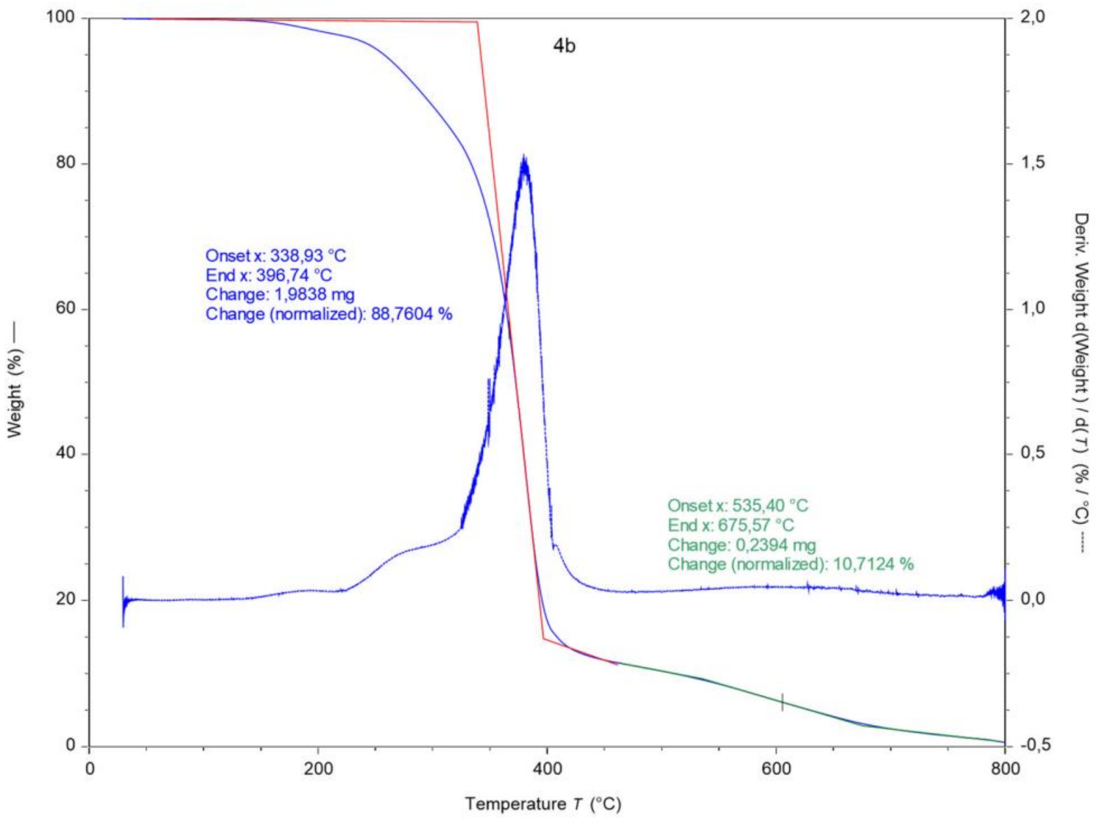

Figure S12. Thermogravimetric analysis of **4b**.

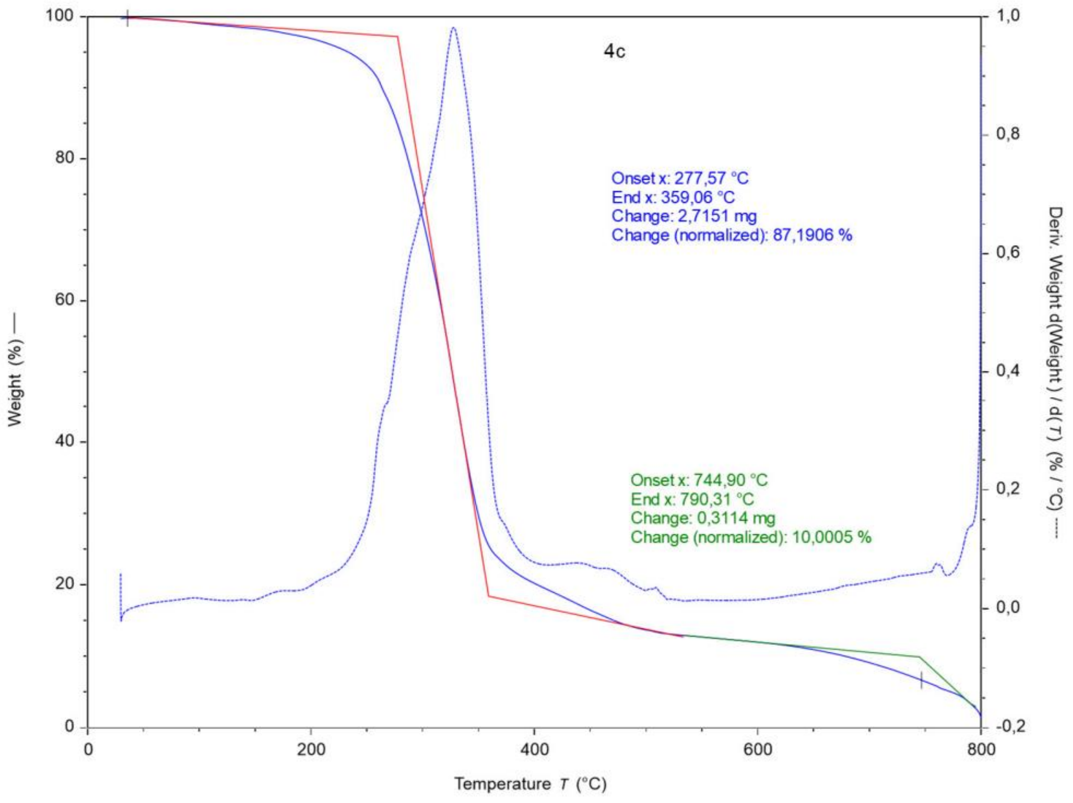

Figure S13. Thermogravimetric analysis of **4c**.

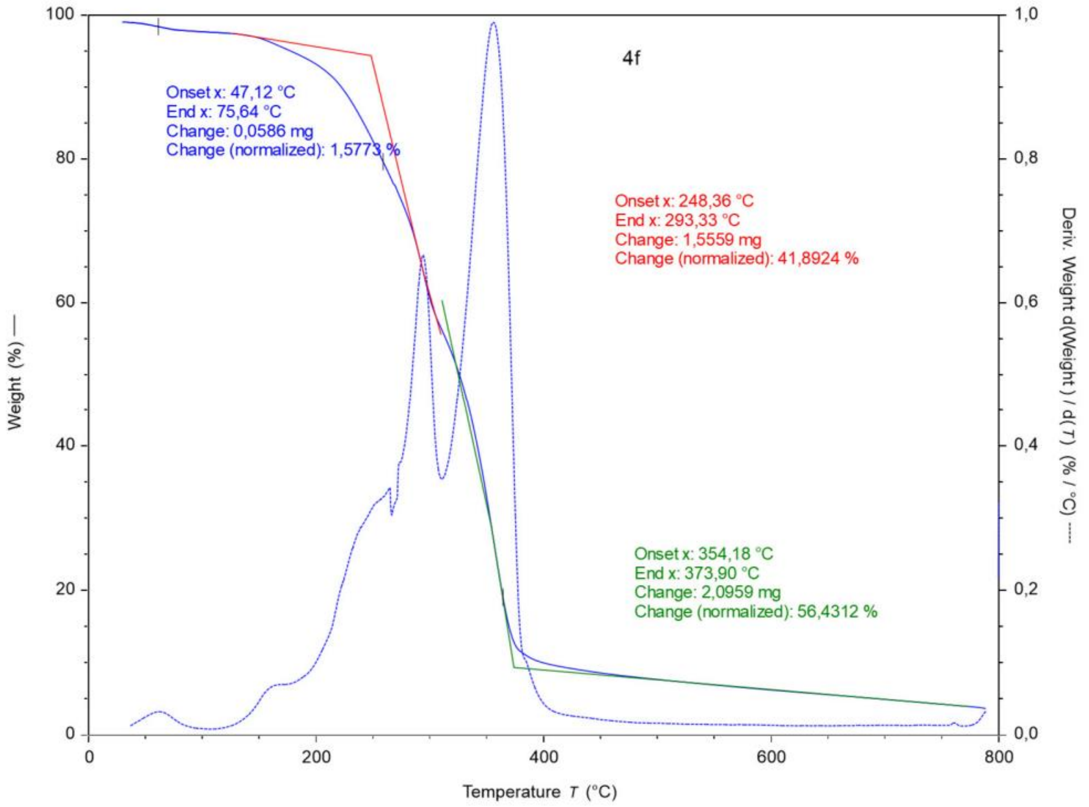

Figure S14. Thermogravimetric analysis of **4f**.

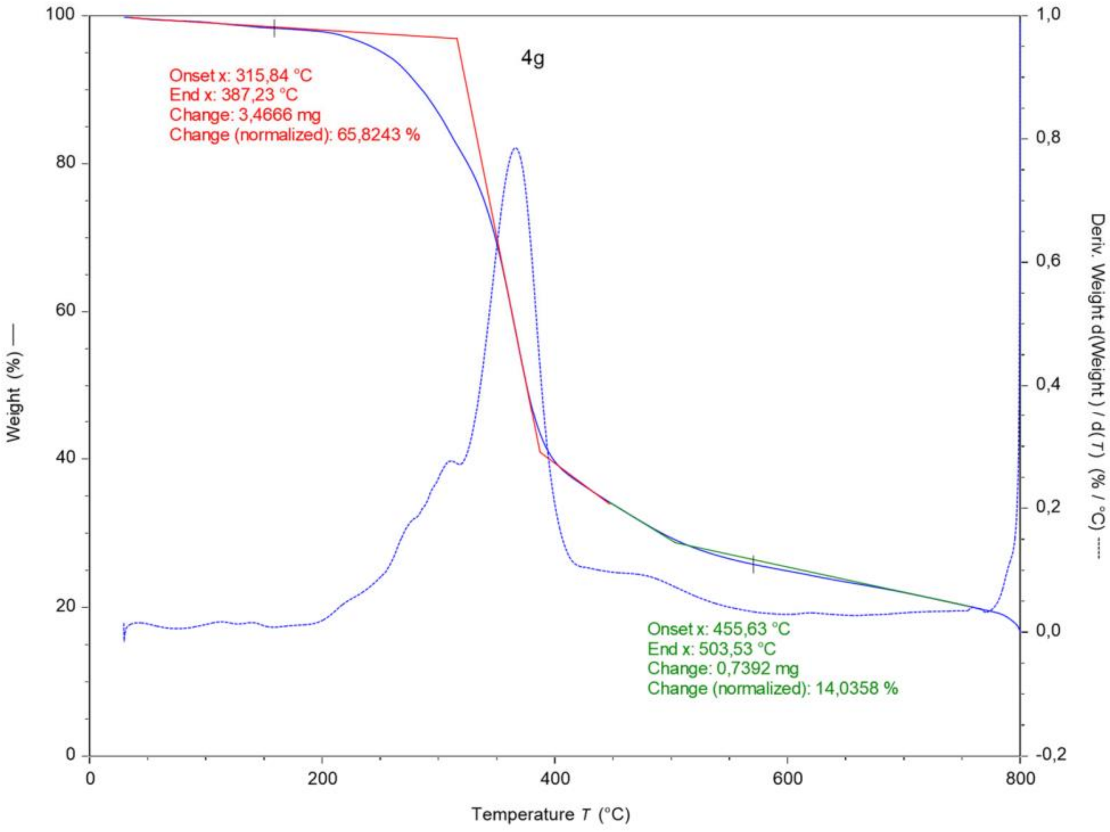

Figure S15. Thermogravimetric analysis of **4g**.

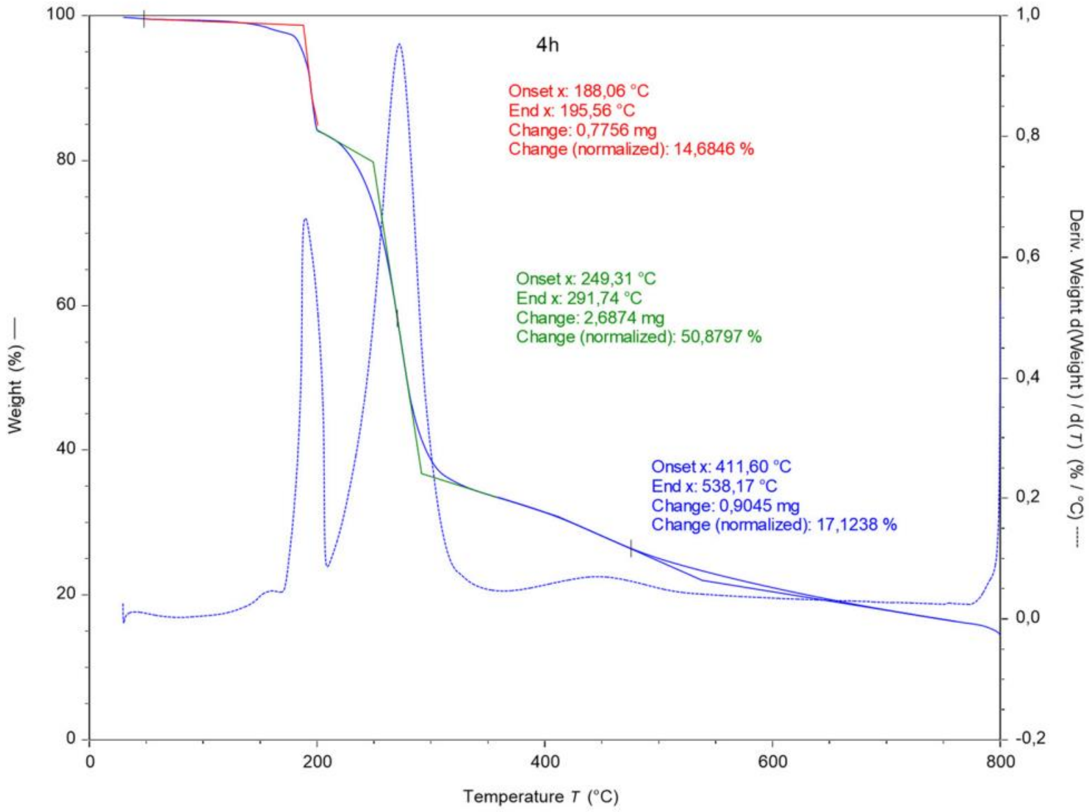

Figure S16. Thermogravimetric analysis of **4h**.

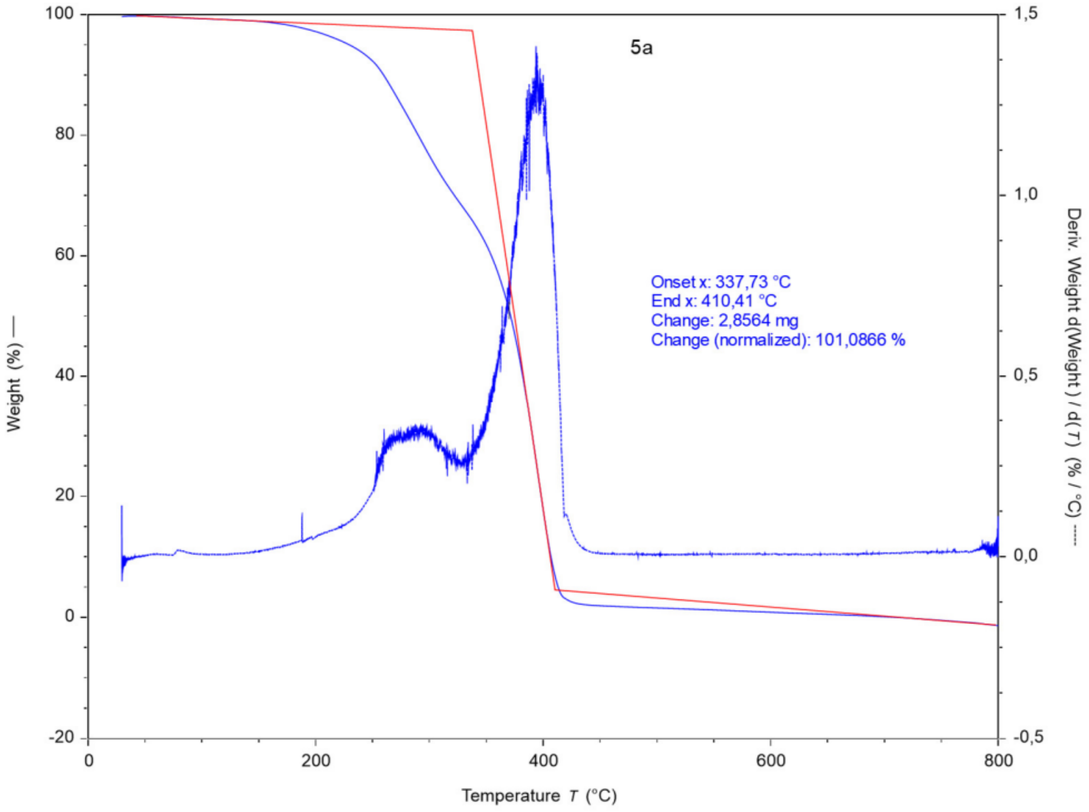

Figure S17. Thermogravimetric analysis of **5a**.

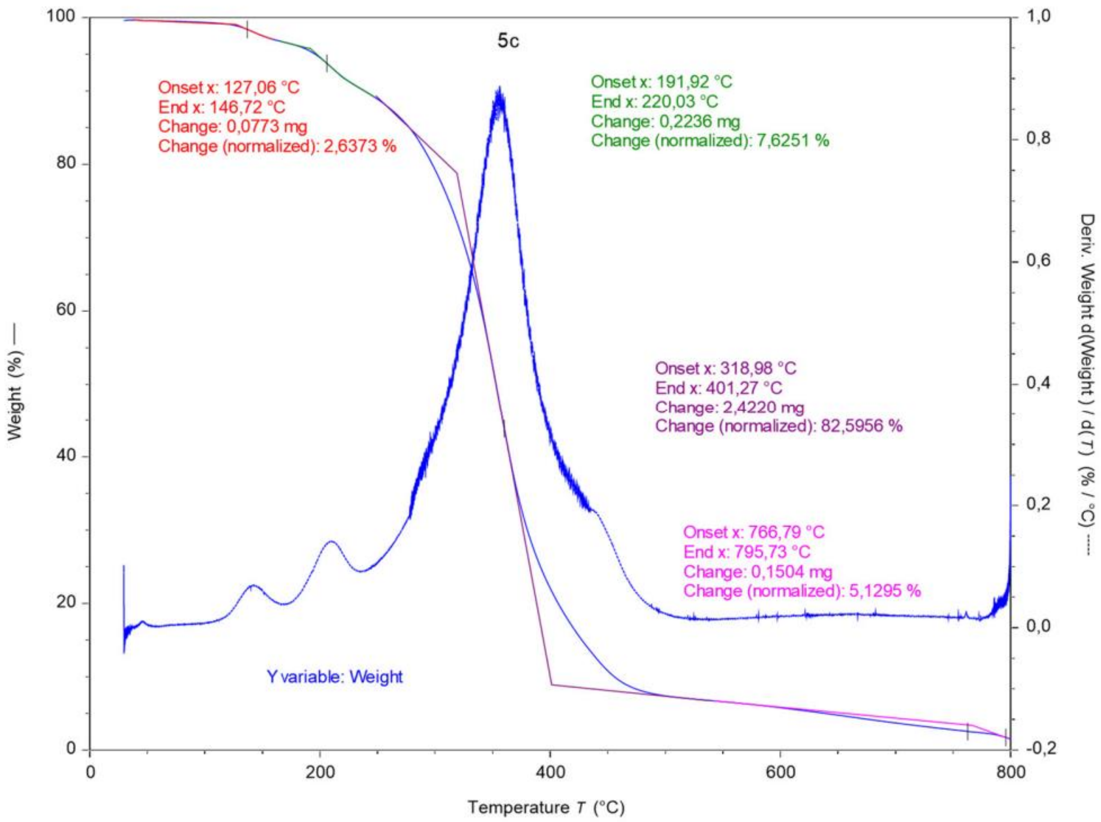

Figure S18. Thermogravimetric analysis of **5c**.

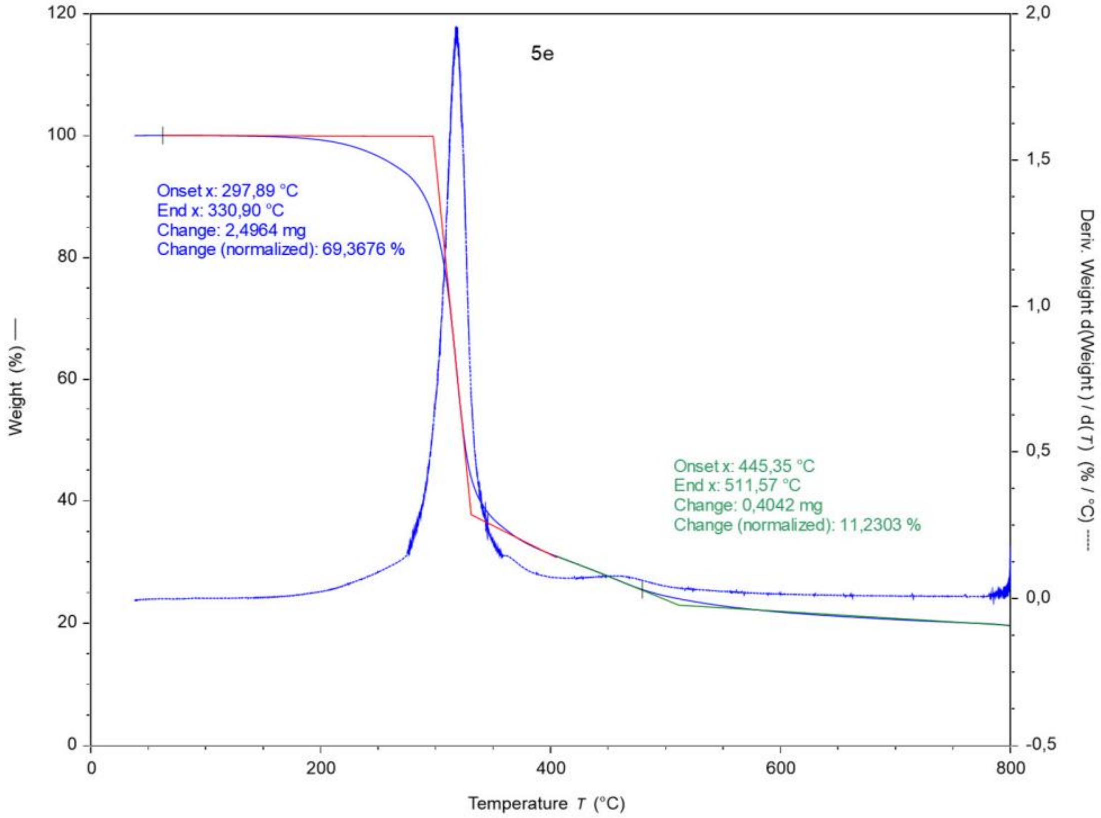

Figure S19. Thermogravimetric analysis of **5e**.

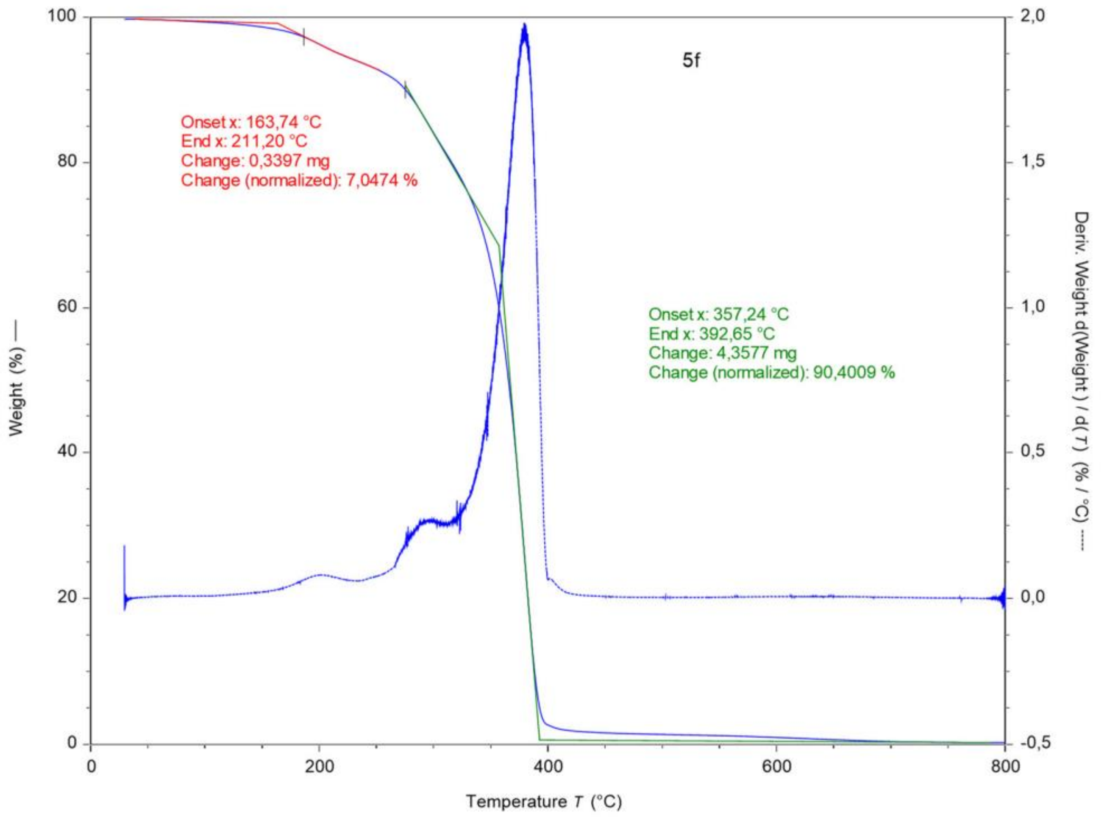

Figure S20. Thermogravimetric analysis of **5f**.

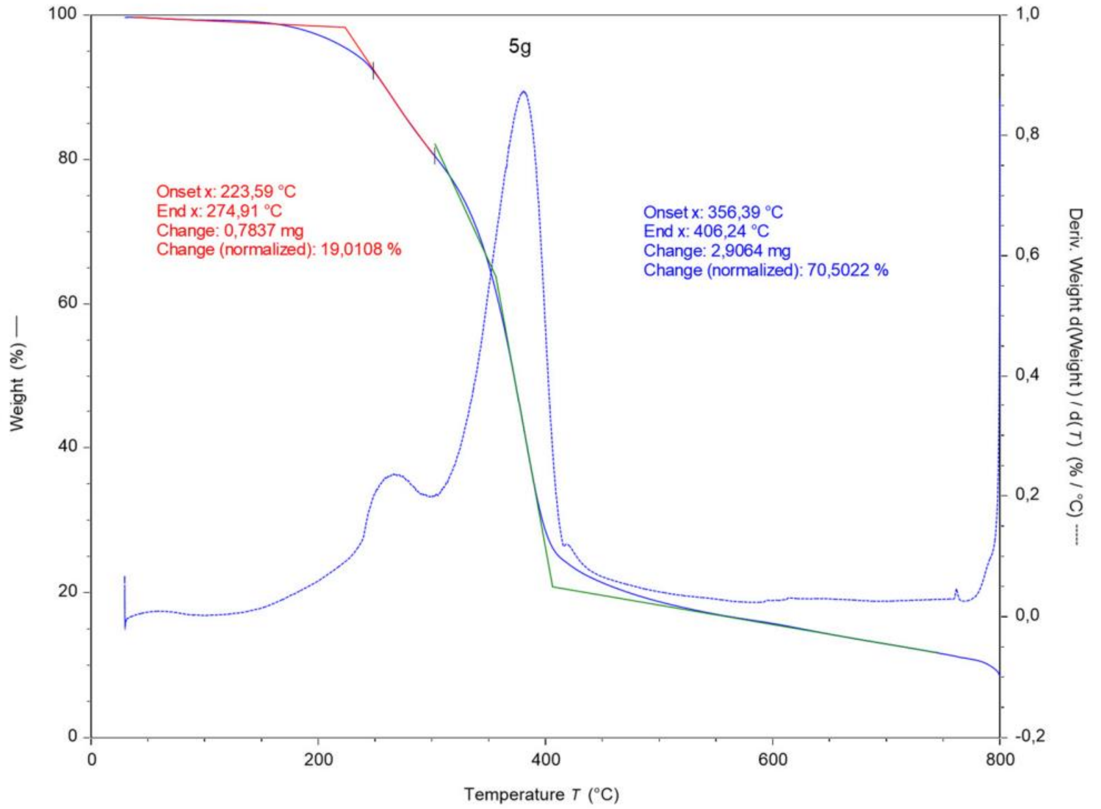

Figure S21. Thermogravimetric analysis of **5g**.

MALDI-TOF: Individual spectra

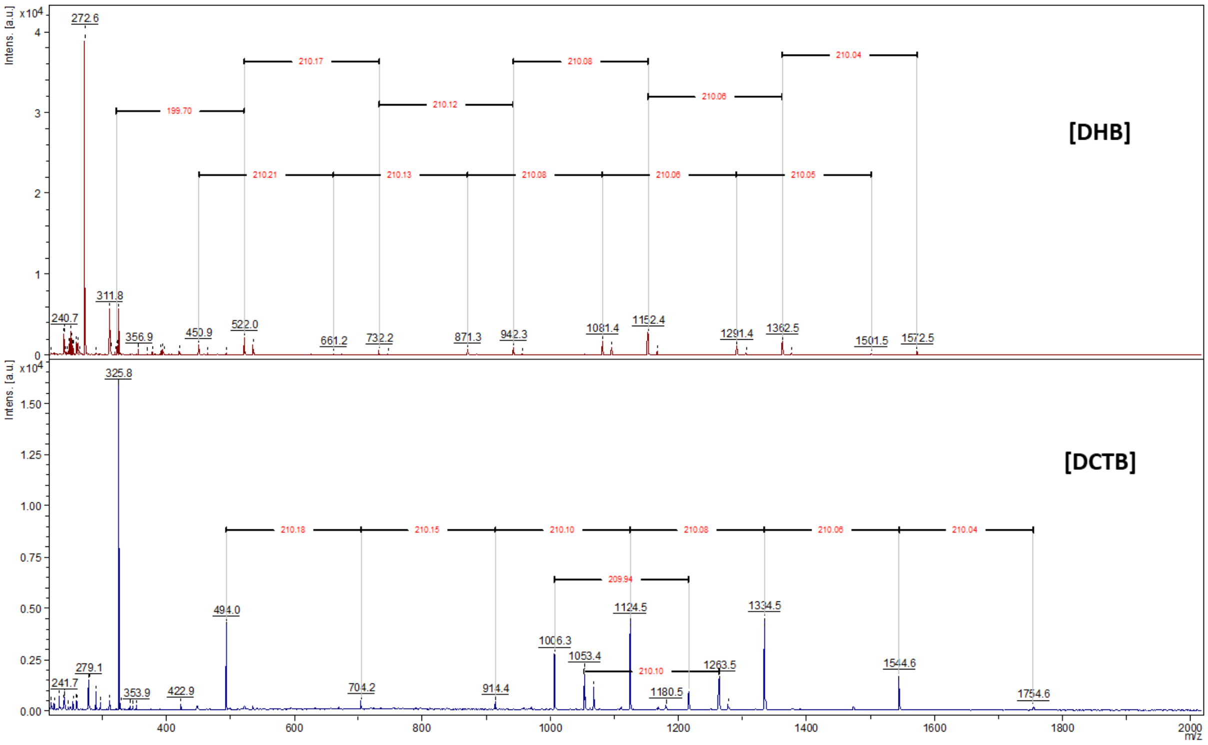

Figure S22. MALDI-TOF mass spectrum of **4a**.

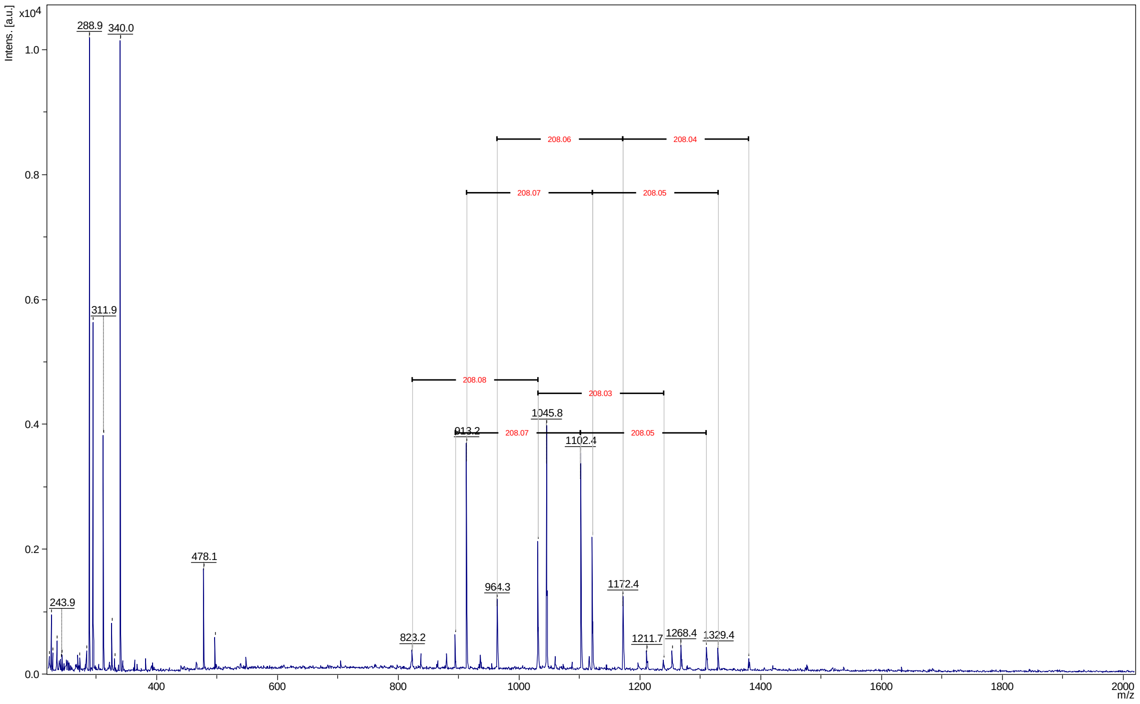

Figure S23. MALDI-TOF mass spectrum of **4d** (DCTB martrix).

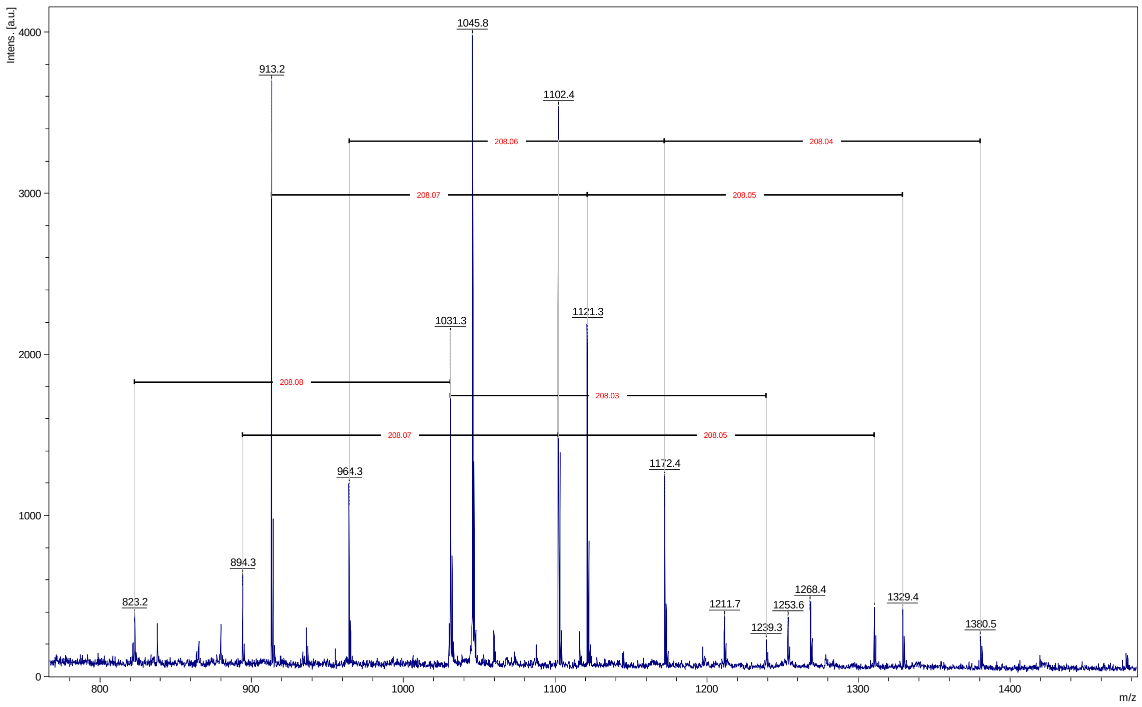

Figure S24. MALDI-TOF mass spectrum amplified of **4d**.

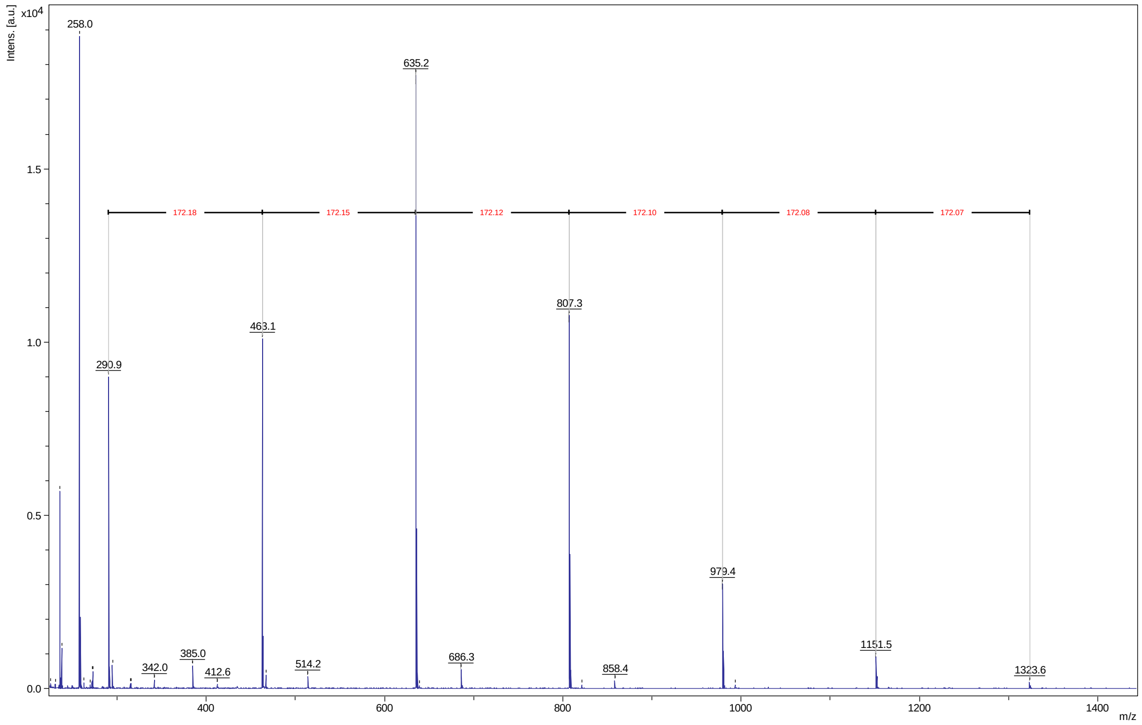

Figure S25. MALDI-TOF mass spectrum of **5a** (DCTB matrix).

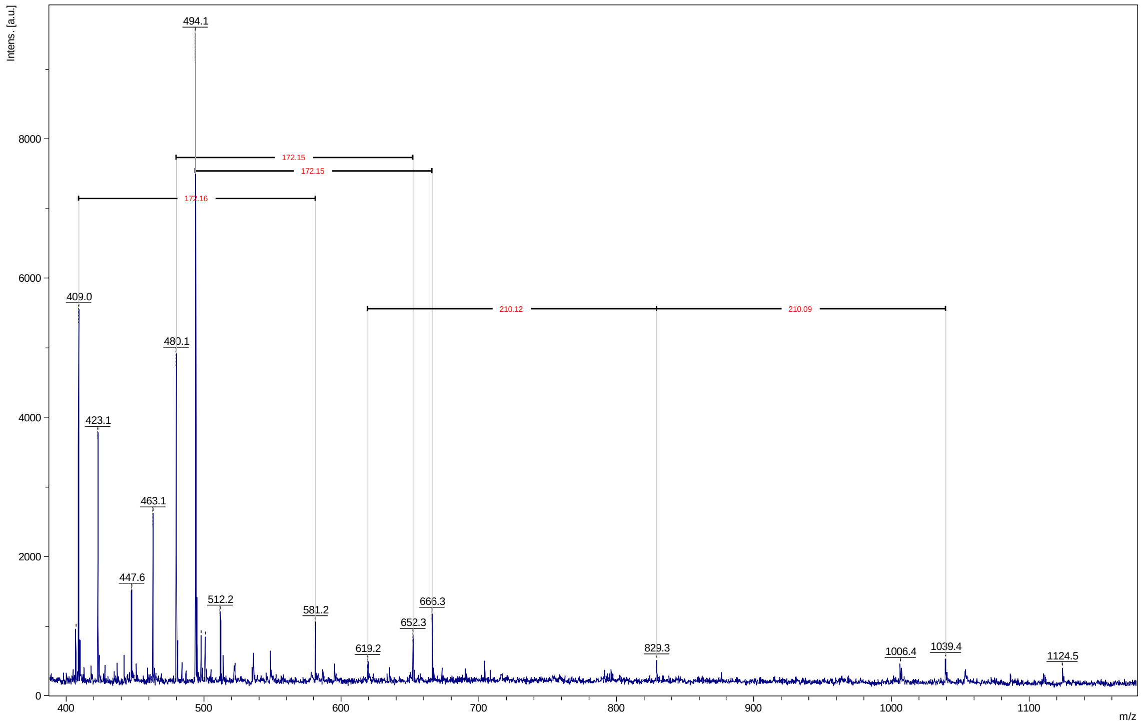

Figure S26. MALDI-TOF mass spectrum of **6a** (DCTB matrix).

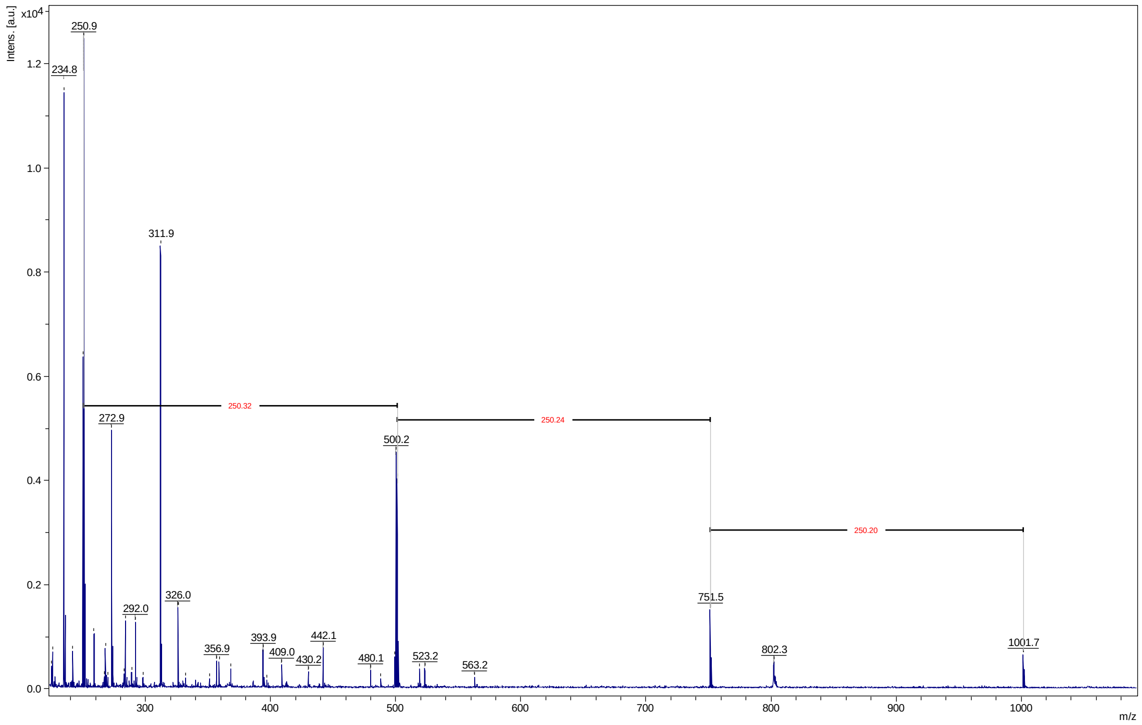

Figure S27. MALDI-TOF mass spectrum of **6a'** (DCTB matrix).

GPC Chromatograms

GPC data summary of polymer samples

The following **Table S2** summarizes the GPC data for the polyester samples analysed in this study.

Table S2. GPC data of the polyesters **4**, **5** and **6**.

Compound	M_n	M_w	M_P	M_z	M_{z+1}	Polydispersity
**4a**	943	1824	1429	3310	5456	1.93449
**4b**	984	2040	1241	3835	6251	2.073008
**4c**	647	1425	1025	2948	5183	2.202404
**4g**	961	2188	1199	4211	6649	2.276449
**4h**	742	1620	1588	2880	4339	2.18287
**5a**	1027	1489	1288	2151	3038	1.450999
**5c**	1799	5140	5916	9920	14329	2.857045
**5e**	1740	3394	3224	6036	8998	1.950845
**5g**	1753	3682	3571	6429	9476	2.100817
**5h**	3356	8588	8521	15129	21763	2.559072
**6a**	1555	3315	2952	5723	8402	2.131877
**6a'**	2629	5720	5634	9709	13670	2.175243

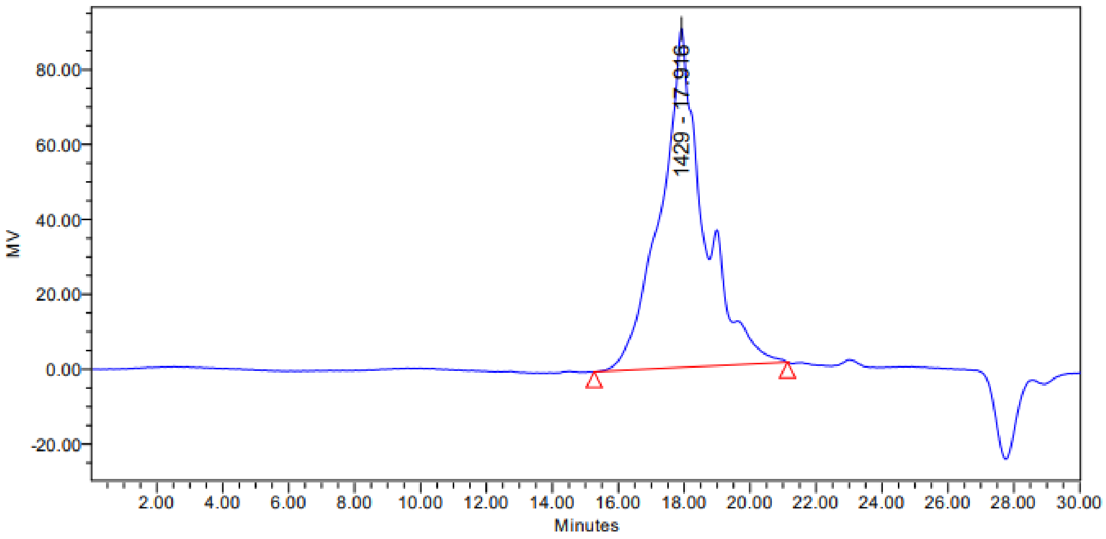

Figure S28. GPC elugram (RI signal, chloroform) of sample **4a** using polystyrene standards (M_n = 943 g/mol, $Đ$ = 1.93). Elution peak observed at a retention time of 17.92 minutes.

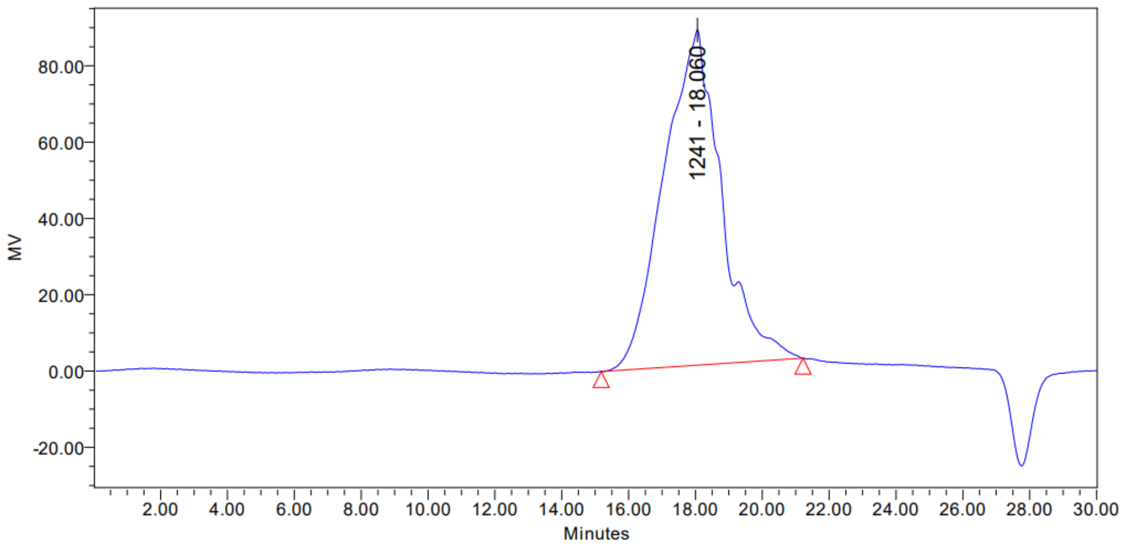

Figure S29. GPC elugram (RI signal, chloroform) of sample **4b** using polystyrene standards (M_n = 984 g/mol, *Đ* = 2.07). Elution peak observed at a retention time of 18.06 minutes.

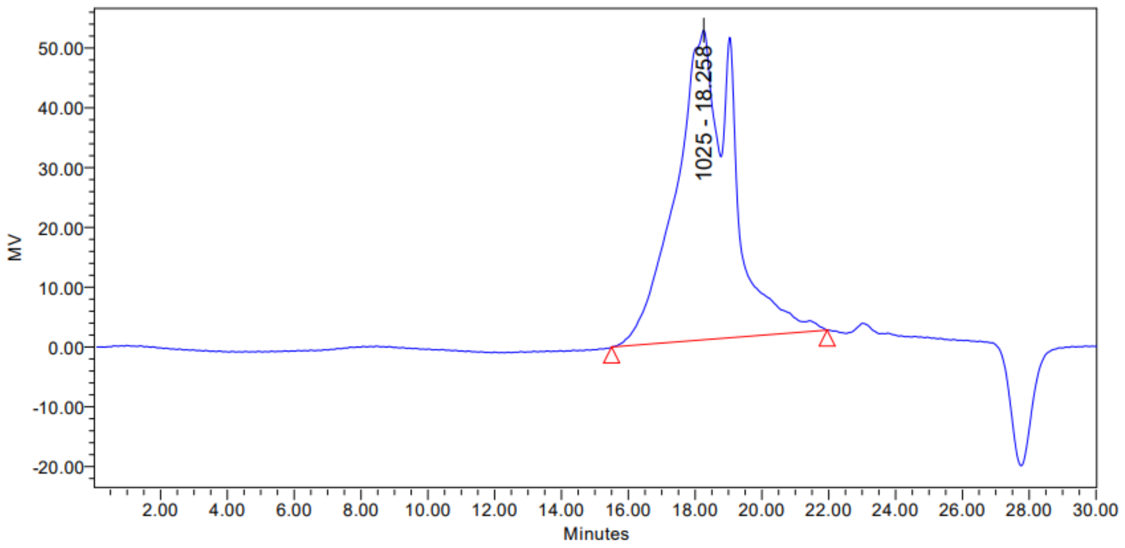

Figure S30. GPC elugram (RI signal, chloroform) of sample **4c** using polystyrene standards (M_n = 647 g/mol, *Đ* = 2.20). Elution peak observed at a retention time of 18.26 minutes.

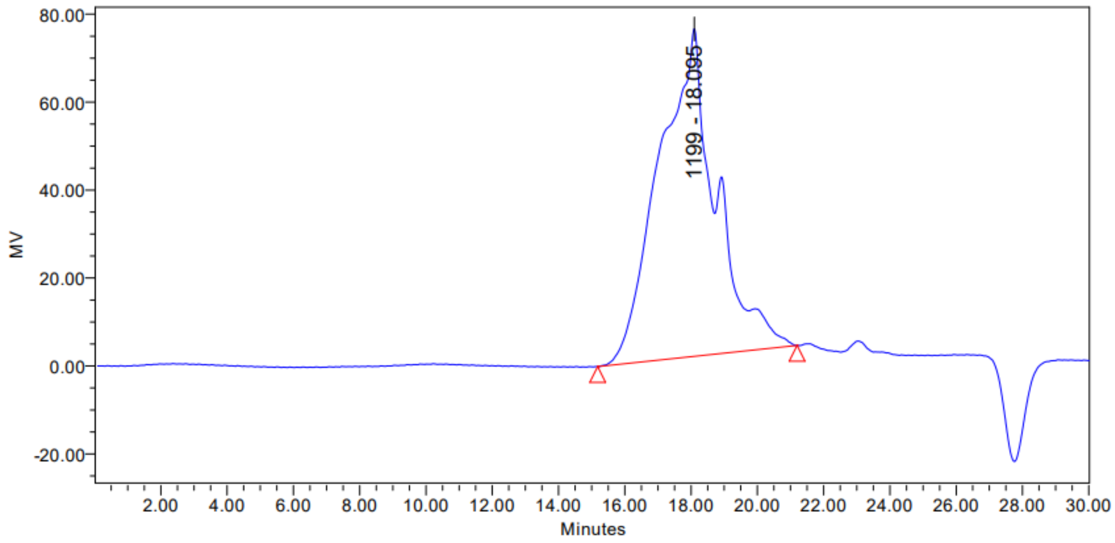

Figure S31. GPC elugram (RI signal, chloroform) of sample **4g** using polystyrene standards (M_n = 961 g/mol, *Đ* = 2.28). Elution peak observed at a retention time of 18.10 minutes.

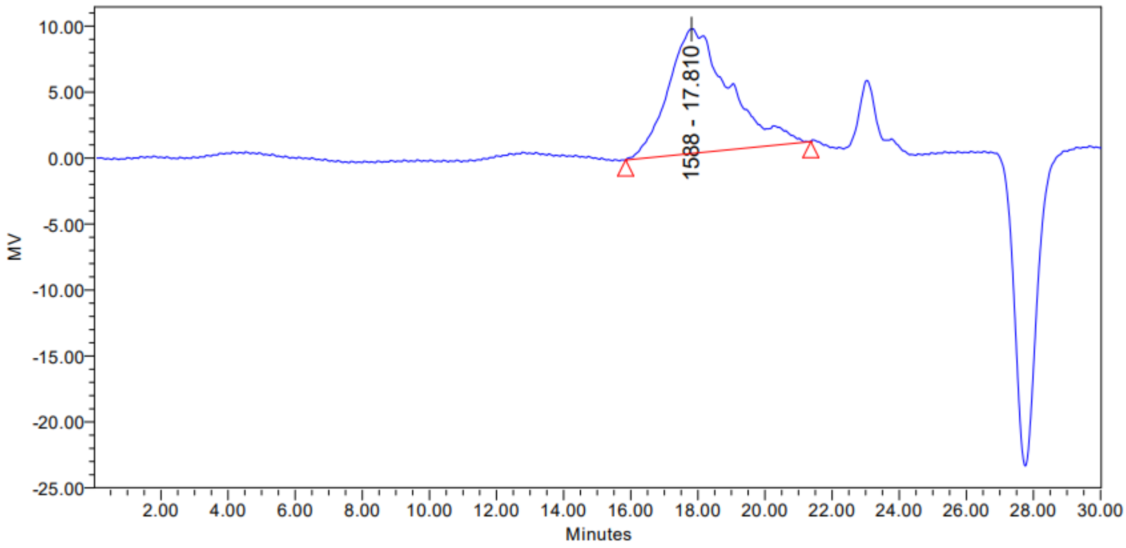

Figure S32. GPC elugram (RI signal, chloroform) of sample **4h** using polystyrene standards (M_n = 742 g/mol, *Đ* = 2.18). Elution peak observed at a retention time of 17.81 minutes.

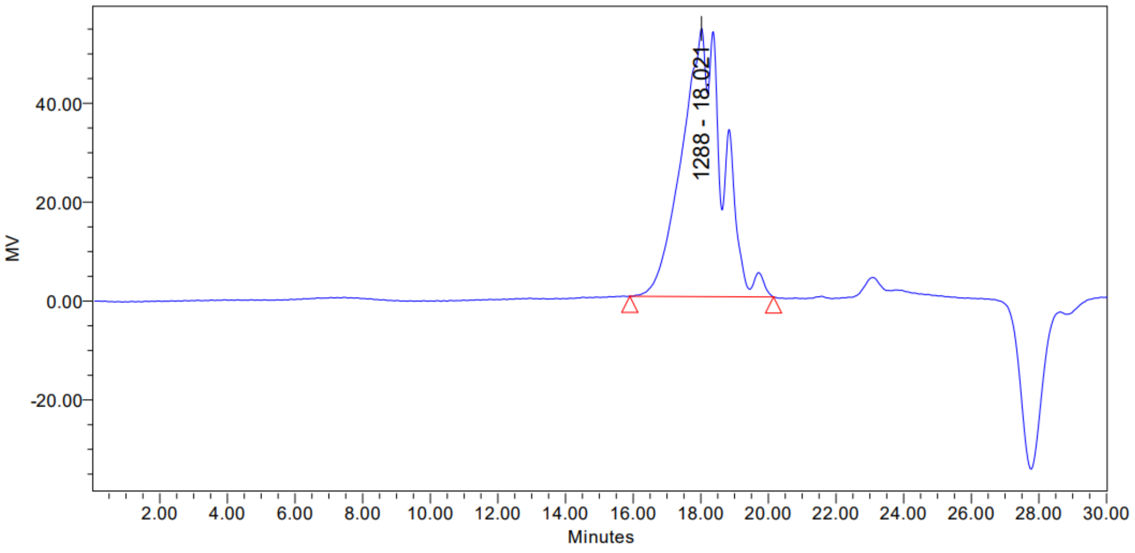

Figure S33. GPC elugram (RI signal, chloroform) of sample **5a** using polystyrene standards (M_n = 1027 g/mol, $Đ$ = 1.45). Elution peak observed at a retention time of 18.02 minutes.

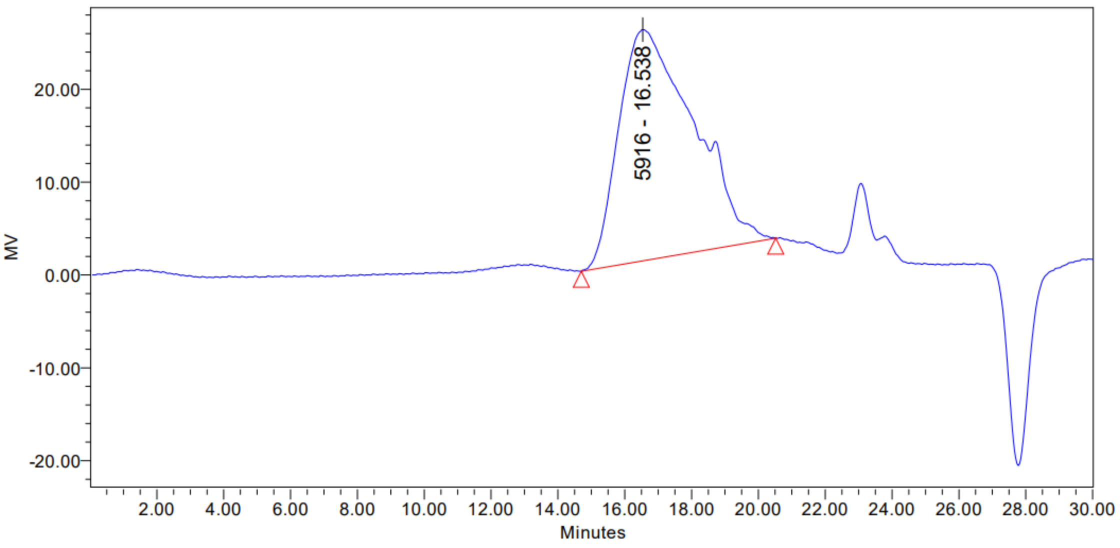

Figure S34. GPC elugram (RI signal, chloroform) of sample **5c** using polystyrene standards (M_n = 1799 g/mol, $Đ$ = 2.86). Elution peak observed at a retention time of 16.54 minutes.

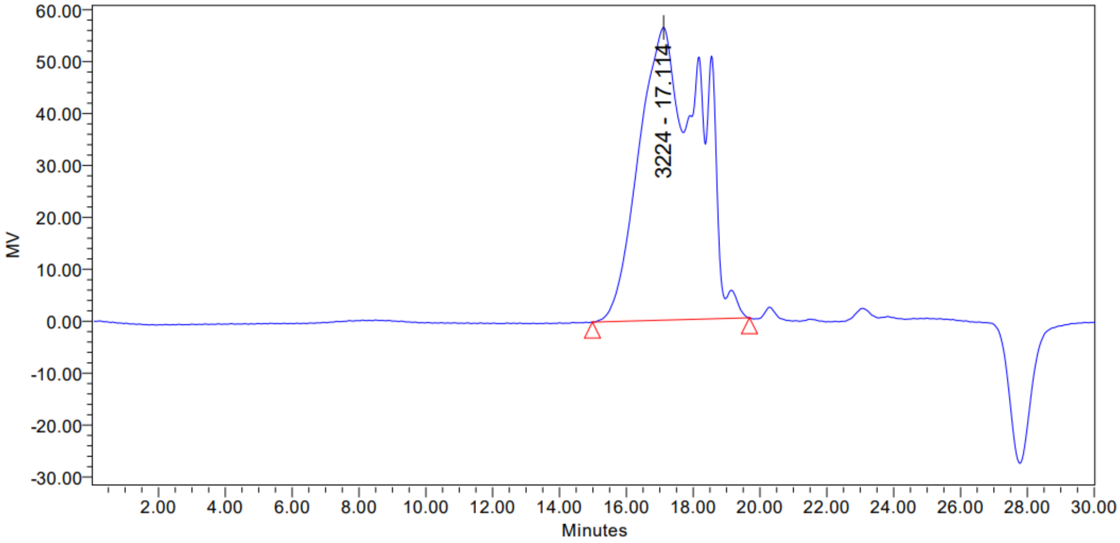

Figure S35. GPC elugram (RI signal, chloroform) of sample **5e** using polystyrene standards (M_n = 1740 g/mol, *Đ* = 1.95). Elution peak observed at a retention time of 17.11 minutes.

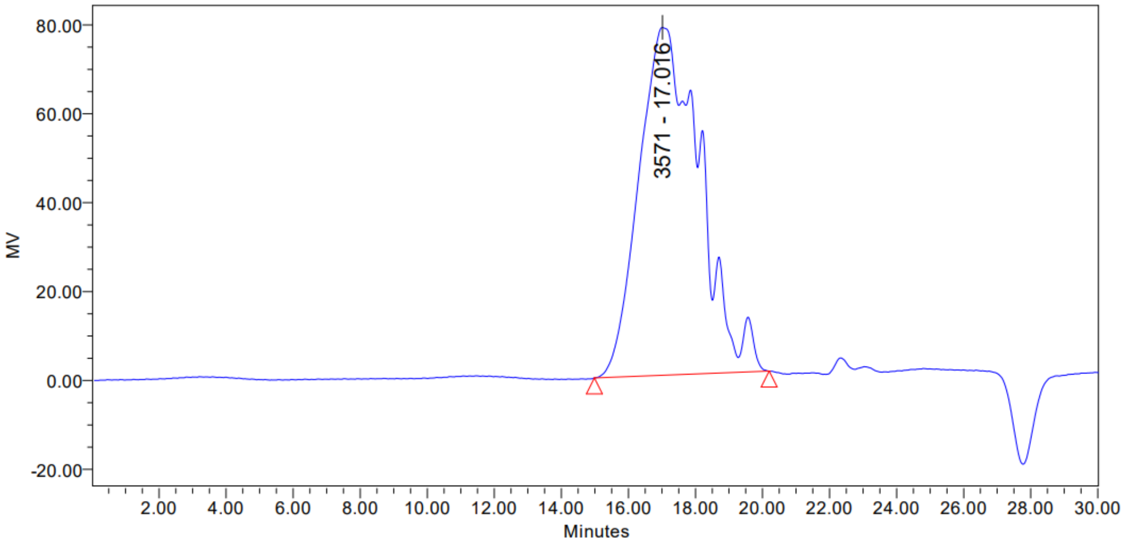

Figure S36. GPC elugram (RI signal, chloroform) of sample **5g** using polystyrene standards (M_n = 1753 g/mol, *Đ* = 2.10). Elution peak observed at a retention time of 17.02 minutes.

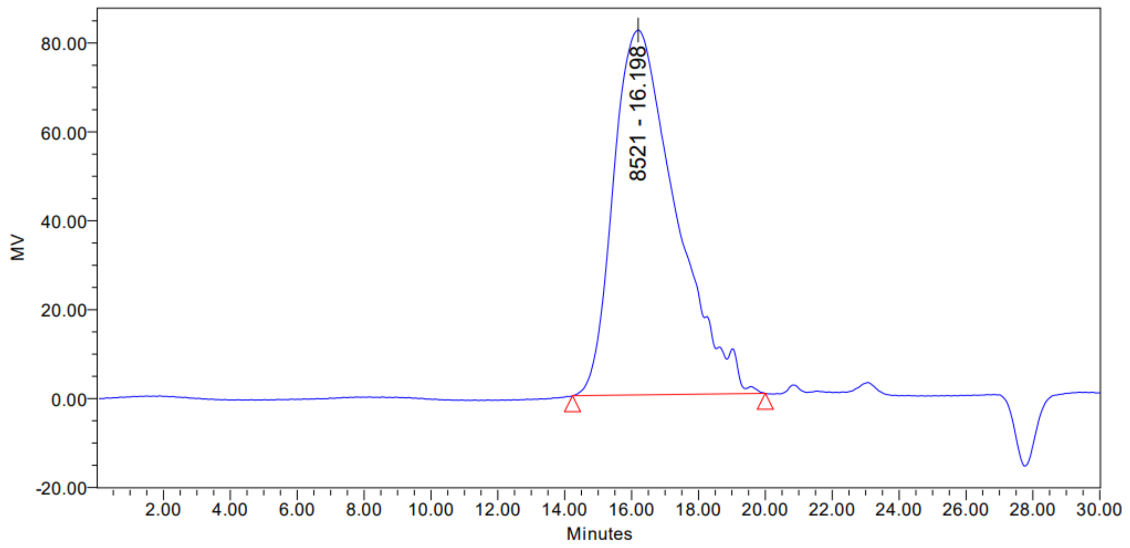

Figure S37. GPC elugram (RI signal, chloroform) of sample **5h** using polystyrene standards (M_n = 3356 g/mol, $Đ$ = 2.56). Elution peak observed at a retention time of 16.20 minutes.

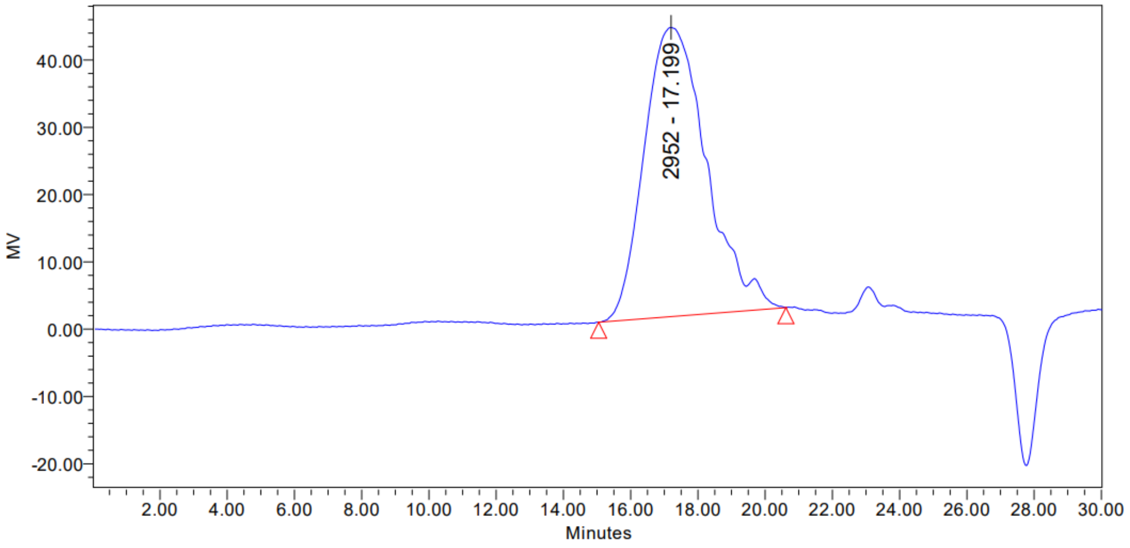

Figure S38. GPC elugram (RI signal, chloroform) of sample **6a** using polystyrene standards (M_n = 1555 g/mol, $Đ$ = 2.13). Elution peak observed at a retention time of 17.20 minutes.

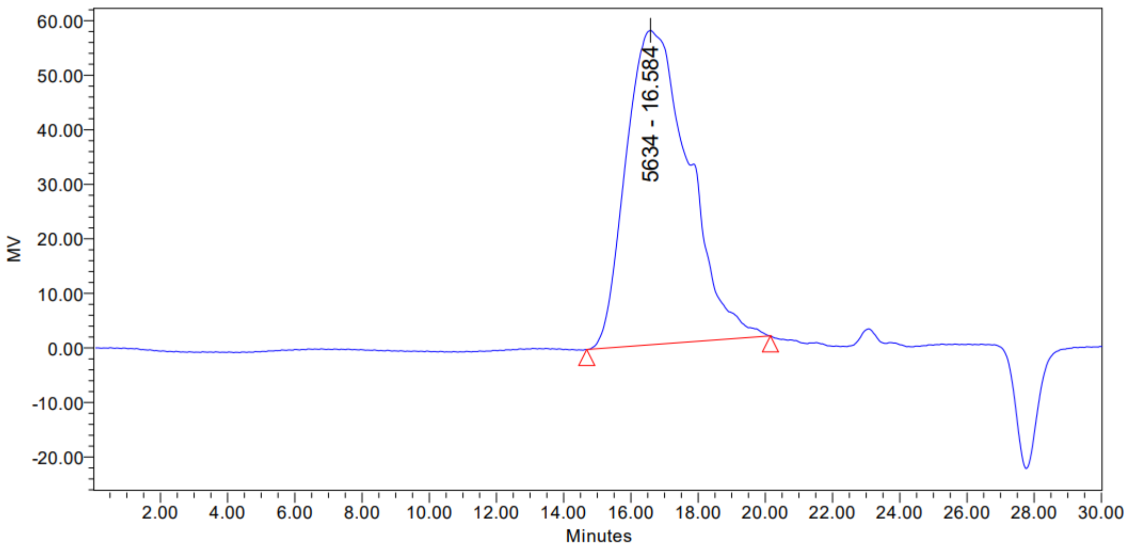

Figure S39. GPC elugram (RI signal, chloroform) of sample **6a'** using polystyrene standards (M_n = 2629 g/mol, $Đ$ = 2.18). Elution peak observed at a retention time of 16.58 minutes.
